# Comparison of Long Noncoding RNA and mRNA Expression Profiles in Mesenchymal Stem Cells Derived from Human Periodontal Ligament and Bone Marrow

**DOI:** 10.1155/2014/317853

**Published:** 2014-03-27

**Authors:** Rui Dong, Juan Du, Liping Wang, Jinsong Wang, Gang Ding, Songlin Wang, Zhipeng Fan

**Affiliations:** ^1^Laboratory of Molecular Signaling and Stem Cells Therapy, Beijing Key Laboratory of Tooth Regeneration and Function Reconstruction, Capital Medical University School of Stomatology, Beijing 100050, China; ^2^Molecular Laboratory for Gene Therapy and Tooth Regeneration, Beijing Key Laboratory of Tooth Regeneration and Function Reconstruction, Capital Medical University School of Stomatology, Beijing 100050, China; ^3^Department of Biochemistry and Molecular Biology, Capital Medical University School of Basic Medical Sciences, Beijing 100069, China; ^4^Department of Stomatology, Yidu Central Hospital, Weifang Medical University, No. 4138 Linglong Mountain South Road, Qinzhou 262500, China

## Abstract

Mesenchymal stem cells (MSCs) in different anatomic locations possess diverse biological activities. Maintaining the pluripotent state and differentiation depend on the expression and regulation of thousands of genes, but it remains unclear which molecular mechanisms underlie MSC diversity. Thus, potential MSC applications are restricted. Long noncoding RNAs (lncRNAs) are implicated in the complex molecular circuitry of cellular processes. We investigated differences in lncRNA and mRNA expression profiles between bone marrow stem cells (BMSCs) and periodontal ligament stem cells (PDLSCs) with lncRNA microarray assays and bioinformatics analysis. In PDLSCs, numerous lncRNAs were significantly upregulated (*n* = 457) or downregulated (*n* = 513) compared to BMSCs. Furthermore, 1,578 mRNAs were differentially expressed. These genes implicated cellular pathways that may be associated with MSC characteristics, including apoptosis, MAPK, cell cycle, and Wnt signaling pathway. Signal-net analysis indicated that phospholipase C beta 4, filamin B beta, calcium/calmodulin-dependent protein kinase II gamma, and the ionotropic glutamate receptor, AMPA 1, had the highest betweenness centrality among significant genes in the differential gene profile network. A comparison between the coding-noncoding gene coexpression networks of PDLSCs and BMSCs identified chemokine (C-X-C motif) ligand 12 as a core regulatory factor in MSC biology. These results provided insight into the mechanisms underlying MSC biology.

## 1. Introduction

Stem cells are undifferentiated cells that can either self-renew or differentiate to produce mature progeny cells [1, 2]. The two major categories are embryonic and adult stem cells. Adult stem cells are undifferentiated cells found in specialized tissues and organs of adults. Compared to embryonic stem cells, adult stem cells that exist in various organs of the body are easily accessible, and their use is less controversial in terms of ethics [3, 4]. Mesenchymal stem cells (MSCs) have been identified as mesoderm-derived stromal cells that can differentiate into various mesoderm-type cell lineages. MSCs hold significant promise for tissue regeneration, due to their potential for self-renewal and multilineage differentiation [5–7]. Humans have abundant adult MSCs available for use in cell-based tissue engineering. MSCs from various tissues, including bone marrow, periosteum, skeletal muscle, and adipose tissue, have similar epitope profiles, but significant differences have been observed in MSC properties; that is, MSCs vary in their differentiation, proliferation, and migration potentials according to the tissue source [8–12]. Traditionally, bone-marrow-derived MSCs (BMSCs) have been studied for bone regeneration applications. BMSCs are a population of multipotent, nonhematopoietic marrow-derived cells that are easily expanded in culture and differentiate into cells with an osteogenic phenotype [13, 14]. BMSC transplantations have enhanced periodontal tissue regeneration and bone formation [15, 16]. Interestingly, Hu and colleagues investigated whether BMSCs might give rise to different types of epithelial cells, and they tested their potential for serving as a source of ameloblasts. Those results showed, for the first time, that BMSCs could be reprogrammed to become ameloblast-like cells [17]. Thus, BMSCs offered a novel approach for tooth-tissue engineering; they could be induced to become both mesenchymal and epithelial cells in tooth applications [17]. However, scientists disagree on whether BMSCs are ideal seeding cells for tooth engineering. Jing pointed out that the differentiation ability of BMSCs decreases significantly with increasing age of the donor [18]. In the past few decades, several new populations of MSCs have been isolated from dental and craniofacial tissues on the basis of their stem cell properties. These new populations included stem cells derived from the periodontal ligament (PDLSCs), from dental pulp, and from apical papilla, among others [19–24]. When transplanted into animals, these dental tissue-derived stem cells could generate bone/dentin-like mineralized tissue, and they were capable of repairing tooth defects and regenerating periodontal tissue [21, 25, 26]. In contrast to BMSCs, these cells were easily accessible, and they were more intimately associated with dental tissues [3]. Although dental tissue-derived MSCs and BMSCs are regulated by similar factors and share a common protein expression profile, these populations differ significantly in their proliferative ability and developmental potentials* in vitro*. Furthermore, importantly, they differ in their ability to develop into distinct tissues representative of the microenvironments from which they were derived* in vivo*. For example, BMSCs formed only bone tissue in the mouse model when treated in the same manner as the dental tissue-derived stem cells [19, 27]. However, the chondrogenic and adipogenic potentials of dental tissue-derived MSCs appeared to be weaker than those of BMSCs [22, 28]. Conversely, the neurogenicity of dental tissue-derived stem cells may be more potent than that of BMSCs, probably due to their neural crest origin [22, 28].

From the time that dental stem cells were first identified, they have been spotlighted in the dental tissue engineering field. Recently, numerous investigators have attempted to use these cells for dental tissue regeneration and assess their potential in preclinical applications [26, 29]. However, little is known about the characteristics of dental stem cells and the molecular mechanism underlying their diverse biological activities; thus, their potential application is restricted. Clues on the molecules that control MSC biology can be obtained by comparing molecular expression in MSCs with different biological activities. The development of microarray methods for large-scale analyses of mRNA gene expression has made it possible to search systematically for key molecules [30, 31]. With the introduction of these genome-wide research techniques, various groups have attempted to describe and compare the gene expression patterns of specialized adult stem cells [32–34]. Long, noncoding RNAs (lncRNAs) are transcribed RNA molecules longer than 200 nucleotides. LncRNAs have been shown to have comprehensive functions in both normal development and disease states [35]. Many studies have revealed that lncRNAs exert important roles in biological processes, including roles in cell differentiation, transcription, imprinting, chromatin modification, and others [36, 37]. Specifically, previous studies have demonstrated that lncRNAs are extremely important for controlling cell or tissue differentiation [38–40].

In this study, we investigated differences in lncRNA and mRNA expression profiles between PDLSCs and BMSCs with microarray assays and bioinformatics analyses. Our results provided useful information for elucidating the different mechanisms that govern MSCs derived from different tissues.

## 2. Materials and Methods

### 2.1. Cell Culture

All research involving human stem cells complied with the International Society for Stem Cell Research “Guidelines for the Conduct of Human Embryonic Stem Cell Research.” We collected impacted, third molars with immature roots from 3 healthy male patients (18–20 years old) under approved guidelines set by the Beijing Stomatological Hospital, Capital Medical University, after obtaining informed patient consent. Molars were removed, disinfected with 75% ethanol, and then washed with PBS. PDLSCs were isolated from each sample, cultured, and identified as previously described [21]. Briefly, PDLSCs were separated from the periodontal ligament in the middle one-third of the root. Then, the tissue was digested in a solution of 3 mg/mL collagenase type I (Worthington-Biochem, USA) and 4 mg/mL dispase (Roche, Germany) for 1 h at 37°C. Single-cell suspensions were obtained by passing the cells through a 70 *μ*m strainer (Falcon, BD Labware, USA). Three separate PDLSC cultures were grown in a humidified, 5% CO_2_ incubator at 37°C in alpha-modified Eagle's medium (*α*-MEM; Invitrogen, California, USA) supplemented with 15% fetal bovine serum (FBS; Invitrogen), 2 mmol/L glutamine, 100 U/mL penicillin, and 100 *μ*g/mL streptomycin (Invitrogen).

BMSCs derived from 18–20-year-old males (*n* = 3) were obtained from Cyagen Biosciences (Guangzhou, China). Three separate BMSC cultures were grown in a humidified, 5% CO_2_ incubator at 37°C, in Dulbecco's MEM (Invitrogen), supplemented with 15% FBS (Invitrogen), 2 mmol/L glutamine, 100 U/mL penicillin, and 100 *μ*g/mL streptomycin (Invitrogen). The culture medium was changed every 3 days. All MSCs were used in subsequent experiments after 3–5 passages.

### 2.2. Microarray Detection

MSCs were grown to 90% confluence; then, the BMSCs (*n* = 3) and PDLSCs (*n* = 3) were briefly rinsed with PBS, lysed, and total RNA was isolated with Trizol reagents (Invitrogen). rRNA was removed from total RNA and purified RNA was amplified and transcribed to produce fluorescent cRNA. Reverse transcription was performed along the entire length of the transcripts, without the 3′ bias, with a random priming method. cDNA was labeled and hybridized to the GeneChip Human Gene 2.0 ST Array (Affymetrix), according to the manufacturer's protocol. After hybridization, washing, and staining, the chip was scanned according to the manufacturer's instructions. Microarray experiments were performed at Genminix Informatic Ltd. (Shanghai, China), a microarray service certified by Affymetrix.

### 2.3. Real-Time RT-PCR Analysis

Real-time, reverse transcription-PCR (RT-PCR) was used to verify the differential expression of genes that were detected on the microarray. Total RNA was isolated from MSCs with Trizol reagents (Invitrogen). For real-time RT-PCR, 2 *μ*g aliquots of RNA as template were combined with random hexamers and reverse transcriptase, according to the manufacturer's protocol (Invitrogen). Real-time PCR reactions were performed with the QuantiTect SYBR Green PCR kit (Qiagen, Germany) and an iCycler iQ Multicolor Real-Time PCR Detection System. The relative level of gene expression was calculated with the 2^−ΔΔCT^ method, as previously described [41]. Primers used for amplifying specific genes are shown in Table 1.

### 2.4. Bioinformatics Analysis

Differentially expressed genes were selected with the TwoClassDif method [9, 42, 43]. Gene ontology (GO) analysis was applied to analyze the main functions of differentially expressed genes. Gene ontology is the key functional classification method used at NCBI. GO can organize genes into hierarchical categories and uncover gene regulatory networks on the basis of biological processes and molecular functions [17, 44]. Based on the Kyoto Encyclopedia of Genes and Genomes (KEGG) database, significantly changed pathways were identified and connected in a pathway network (Path-net), where connections were based on the relationship between these pathways. This approach was previously used to summarize the pathway interactions among genes that were differentially expressed under the influence of disease, and it revealed why certain pathways were activated [45].

Based on the GO and KEGG pathway analyses, we established an interactions repository (Signal-net) derived from KEGG to show the core genes that played an important role in this MSC gene network [46, 47]. To determine the interactions among genes, we constructed a coding-noncoding gene coexpression network (CNC network), which has also been called a gene coexpression network. This CNC network was based on a correlation analysis that evaluated associations between differentially expressed lncRNAs and mRNAs [45]. We calculated the Pearson correlation for each pair of genes and used the most significantly correlated pairs to construct the network [48]. The purpose of network structure analysis was to locate core regulatory factors (genes). In the network, the core regulatory factors were those connected to large numbers of adjacent genes, and, thus, they exhibited the greatest degrees of connectivity. In considering different networks, we evaluated the core regulatory factors by the degree of difference they showed in their roles in the PDLSC and BMSC networks [49], which was measured with the variable Diffk (difference in normalized connectivities).

### 2.5. Statistics

All statistical calculations were performed with SPSS10 statistical software. Statistical analyses included comparisons with the* t*-test, Fisher's exact test, *χ* ^2^test, and the Pearson correlation, as appropriate; *P* values less than 0.05 were considered statistically significant.

## 3. Results

### 3.1. Comparison of lncRNA and mRNA Expression Profiles between PDLSCs and BMSCs

To reveal the molecular mechanisms underlying MSCs derived from different tissues, we screened the gene expression patterns in PDLSCs and BMSCs with the human GeneChip microarray method. Because we included only three samples in each group, we applied the RVM* t*-test, which can effectively raise the degrees of freedom in analyses of small sample sizes to filter the genes that were differentially expressed in PDLSCs and BMSCs. After determining significant differences and the false discovery rate (FDR) in the analysis, the differentially expressed genes were selected according to the *P* value threshold. Hierarchical clustering showed systematic variations in the expression of lncRNAs and mRNAs between PDLSCs and BMSCs. From the microarray data, a comparison of lncRNA expression levels between PDLSCs and BMSCs identified an average of 970 lncRNAs that were significantly differentially expressed (see Supplementary Table  1 in Supplementary Material available online at http://dx.doi.org/10.1155/2014/317853); of those, 457 were upregulated and 513 were downregulated in the PDLSCs compared to the BMSCs. In addition, a total of 1,578 mRNAs were differentially expressed in the PDLSCs and BMSCs (Supplementary Table  2); of those, 862 were upregulated and 716 were downregulated in the PDLSCs compared to the BMSCs.

**Figure 1:**
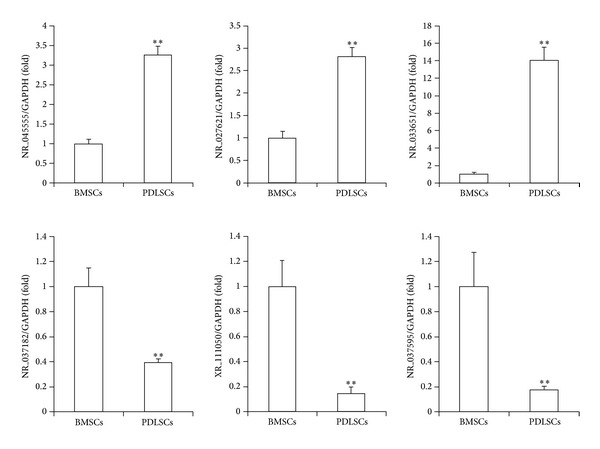
Real-time RT-PCR results show differential lncRNA expression levels in stem cells derived from bone marrow (BMSCs) or periodontal ligament tissue (PDLSCs). The lncRNAs coded as NR_045555, NR_027621, and NR_033651 showed increased expression in PDLSCs, and the lncRNAs coded as NR_037182, XR_111050, and NR_037595 showed decreased expression in PDLSCs compared to BMSCs. GAPDH was used as an internal control. Student's* t*-test was performed to determine statistical significance; all error bars represent s.d. (*n* = 3 tissue samples); **P < 0.01.

**Figure 2:**
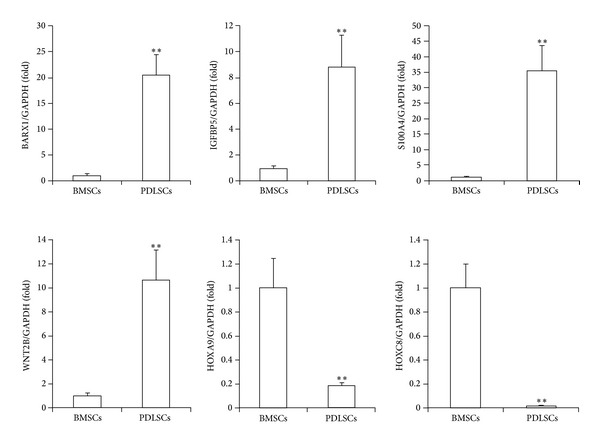
Real-time RT-PCR results show differential mRNA expression levels in stem cells derived from bone marrow (BMSCs) or periodontal ligament tissue (PDLSCs). The mRNAs BARX1, IGFBP5, S100A4, and WNT2B showed increased expression, and the mRNAs HOXA9 and HOXC8 showed decreased expression in PDLSCs compared to BMSCs. GAPDH was used as an internal control. Student's* t*-test was performed to determine statistical significance; all error bars represent s.d. (*n* = 3 tissue samples); **P < 0.01.

To confirm the reliability of the microarray data, we randomly selected six lncRNAs among the 970 differentially expressed lncRNAs and analyzed their expression with real-time RT-PCR. These data confirmed that, compared to BMSCs, PDLSCs showed increased expression of the lncRNAs coded as NR_045555, NR_027621, and NR_033651, and decreased expression of the lncRNAs coded as NR_037182, NR_037595, and XR_111050 (Figure 1). Similarly, we randomly selected six mRNAs among the 1,578 differentially expressed mRNAs and analyzed their expression with real-time RT-PCR. These data confirmed that the mRNAs BARX1, S100A4, WNT2B, and IGFBP5 were increased and that the mRNAs HOXA9 and HOXC8 were decreased in PDLSCs compared to BMSCs (Figure 2). The expression levels of these 12 genes were consistent with the microarray results; thus, these results confirmed the reliability of the microarray data.

### 3.2. Bioinformatics Analysis of BMSC and PDLSC Microarray Data

Next, we performed a bioinformatics analysis to discover the key factors that controlled MSC functions. First, a GO analysis was applied to analyze the main functions of the differentially expressed genes according to gene ontology, which is the key functional classification used by NCBI. According to the threshold, the analysis determined which GOs were significantly differently regulated between PDLSCs and BMSCs with a *P* value and FDR < 0.05. The negative logarithm of the *P* value (-LgP) was used to represent the correlation between gene expression and the relevant biological process. The GO analysis identified 166 genes that were significantly upregulated and 104 that were downregulated among all differentially expressed genes in PDLSCs (data not shown). The results clearly showed which important functions were involved with the differentially expressed genes. The top five upregulated GO functions (upGOs) were related to the response to the mitotic cell cycle, the *M* phase of the mitotic cell cycle, mitotic prometaphase, the cell cycle checkpoint, and mitotic sister chromatid segregation (Supplementary Figure  1). The top five downregulated GO functions (downGOs) were related to the anterior/posterior pattern, embryonic skeletal system morphogenesis, signal transduction, cochlea morphogenesis, and blood vessel remodeling (Supplementary Figure  2).

**Figure 3:**
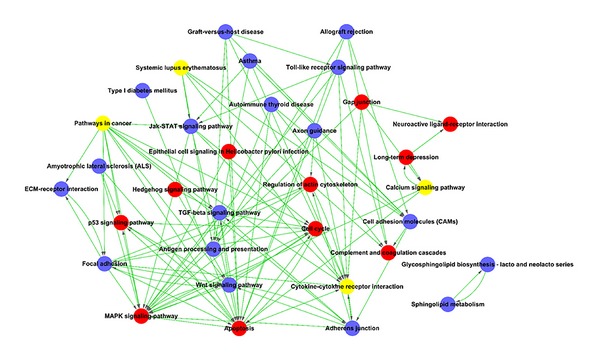
The interaction network of significant pathways (Path-net) in stem cells. Pathways that were significantly different between PDLSCs and BMSCs were connected in a Path-net diagram to show the relationships between these pathways. The role of each pathway in the network was measured by counting its connections to upstream and downstream pathways, known as in-degree (upstream connections), out-degree (downstream connections), or degree (all connections). A high degree pathway indicated that it regulated or was regulated by many other pathways, which implied an important role in the signaling network. The circles represent the pathways; blue represents downregulated pathways, red represents upregulated pathways, and yellow represents up- and downregulated pathways. The lines indicate interactions between pathways.

Based on the KEGG database, we identified the pathways that mediated the functions of the differentially expressed genes. We identified a total of 67 pathways that showed significant differences due to differential gene expression; changes in 31 pathways involved upregulated genes and changes in 36 pathways involved downregulated genes (Supplementary Figures  3 and  4). We performed Path-net analysis to generate an interaction network that included these significantly changed pathways (Figure 3). The top 3 upregulated pathways were apoptosis, MAPK, and cell cycle signaling. The top 3 downregulated pathways were focal adhesion, Wnt, and adherens junction signaling. In addition, cytokine-cytokine receptor interactions and pathways related to cancer were up-/downregulated. These data suggested that these pathways may play key roles in the different core epigenetic mechanisms of PDLSCs and BMSCs.

**Figure 4:**
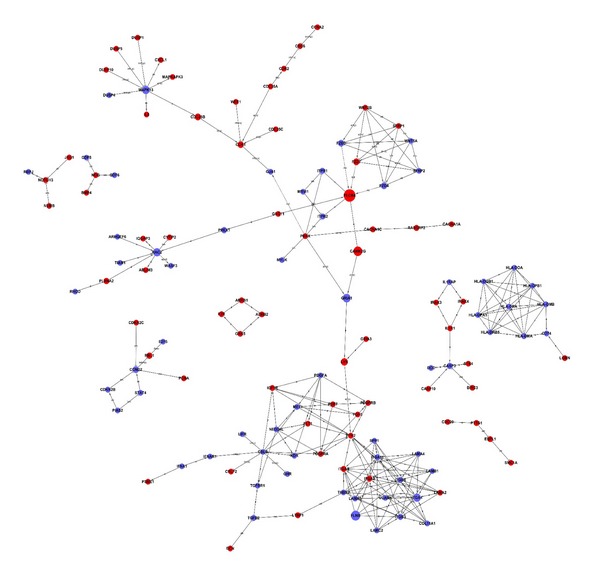
The interaction network of differentially expressed genes (Signal-net). The circles represent important functional genes in PDLSCs (red: upregulated genes; blue: downregulated genes); the circle size represents the degree of interaction (betweenness centrality), and lines indicate the interactions.

We performed a Signal-net analysis to further investigate the global network, based on the significantly regulated GOs and pathways. With Signal-net, we screened important candidate genes involved in the differences between PDLSCs and BMSCs (Figure 4). In the Signal-net analysis, the genes are characterized by measuring their “betweenness centrality,” the number of times a node is located in the shortest path between 2 other nodes. This measure reflects the importance of a node in a graphic network relative to other nodes. The four most important differentially expressed genes were identified in the network (Supplementary Table  3); these were phospholipase C beta 4 (PLC*β*4), filamin B beta (FLNB), calcium/calmodulin-dependent protein kinase II gamma (CAMK2G), and the ionotropic glutamate receptor, AMPA 1 (GRIA1).

Finally, we used a coding-noncoding gene coexpression (CNC) network to evaluate the interactions among genes and identify the core regulatory genes in the network. Based on our previous results, we built CNC networks to identify the interactions among the differentially expressed lncRNAs and mRNAs in PDLSCs and BMSCs [45]. We used 65 lncRNAs and 208 mRNAs to build the CNC network for PDLSCs and 75 lncRNAs and 187 mRNAs to build the network for BMSCs. In the CNC networks, each mRNA could correlate with one to tens of lncRNAs and vice versa. We used the CNC networks to implicate the interregulation of lncRNAs and mRNAs in the different molecular mechanisms of PDLSCs and BMSCs (Supplementary Figures  5 and  6). In the CNC network of PDLSCs, 17 genes showed a degree ≥ 59 and a clustering coefficient ≥ 0.6. This indicated that these genes, including 4 lncRNAs and 13 mRNAs (Table 2), played important roles in the network. In the CNC network of BMSCs, 20 mRNAs showed a degree ≥ 29 and a clustering coefficient ≥ 0.7. This indicated that (Table 3) these genes played important roles in the network. According to the Diffk values (|Diffk| ≥ 0.75) for these networks, 16 genes (Table 4), including 2 lncRNAs and 14 mRNAs, showed different connectivities between PDLSCs and BMSCs, indicating that their roles were different in core pathways that governed MSC functions. The top three mRNAs were chemokine (C-X-C motif) ligand 12 (CXCL12), integrin alpha 2 (ITGA2, CD49B), and cell division cycle 20 homolog (CDC20), which were upregulated. The two lncRNAs identified (|Diffk| ≥ 0.75) were FR020479 and FR191603; the former was downregulated and the latter was upregulated.

## 4. Discussion

The presence of different MSCs in dental and craniofacial tissues has encouraged clinical studies to investigate tissue regeneration in orofacial and periodontal regions [50, 51]. In the past few decades, MSC-mediated tissue regeneration has made surprising progress [25, 26, 52]. However, bone marrow has remained the principal source of MSCs for most preclinical and clinical applications. Interestingly, the MSCs from different anatomic locations possess diverse biological activities [8–12]. The challenge lies in identifying the specific genes that are associated with distinct MSC functions. To that end, in the present study, we identified lncRNAs and mRNAs that were differentially expressed in PDLSCs and BMSCs.

We identified 970 differentially expressed lncRNAs and 1,578 differentially expressed mRNAs in BMSCs and dental tissue-derived MSCs. This information may be useful for further studies on gene functions and regulation mechanisms in MSCs. Furthermore, we found that several of the upregulated genes in PDLSCs may be associated with PDLSC characteristics. For instance,* BARX1*, a transcription factor expressed in the mesenchyme of molar primordia, is involved in the regulation of tooth morphogenesis, in the development of tooth and craniofacial mesenchyme that originates from the neural crest [53–55], and possibly, in the regulation of MSC differentiation.

To identify the key factors that regulated MSC functions, we applied bioinformatics analyses to classify the microarray data. The GO analysis revealed specific functional pathways that were enriched in the genes responsible for the divergent features of PDLSCs and BMSCs. These differentially expressed genes were subsequently organized into hierarchical categories based on pertinent biological processes. A high degree pathway interacted with a high number of other pathways, which implied an important role in cell biological features. Further pathway analyses indicated that apoptosis, MAPK, cytokine-cytokine receptor interaction, focal adhesion, pathways in cancer, Wnt, cell cycle, and adherens junctions signaling pathways were involved in the diverse biological activities of PDLSCs and BMSCs. It is well known that these pathways play an important role in regulating cellular apoptosis, survival, and differentiation.

To identify important genes involved in the different epigenetic mechanisms of PDLSCs and BMSCs, we performed Signal-net analysis on the significantly regulated GOs and pathways. This analysis revealed that PLC*β*4, FLNB, CAMK2G, and GRIA1 exhibited the most betweenness centrality. PLC*β*4 and CAMK2G were upregulated in PDLSCs. It was reported that PLC*β*4 was highly expressed in the retina and the cerebellum, where calcium plays an important role in the transduction of extracellular signals [56–58]. Moreover, CAMK2G is activated by intracellular calcium/calmodulin [59]. Thus, the Signal-net analysis results suggested that these genes were important in calcium-sensitive signaling cascades that regulate cell function. In addition, FLNB regulates intracellular communication and signaling by linking the protein actin to the cell membrane. This activity allows direct communication between the cell membrane and the cytoskeletal network, which provides a means to control and guide proper skeletal development [60, 61].

The CNC network comparisons indicated that CXCL12 was a core regulatory factor, which may be involved in the diverse biological activities of PDLSCs and BMSCs. CXCL12, also known as stromal cell-derived factor-1, stimulates migration by rearranging the actin cytoskeleton, increasing focal adhesion, and stimulating matrix metalloproteinase production in MSCs [62, 63]. Thus, CXCL12 can recruit MSC to participate in the regeneration of injured tissues [64]. Presumably, MSC migration is mediated through an intracellular pathway, for example, the MAPK/ERK signaling pathways [62]. Our results were consistent with previous reports and may also be applicable to the differentiation mechanisms previously described in MSCs.

Additionally, we identified some lncRNAs that were differentially expressed in PDLSCs and BMSCs, for example, FR020479 and FR191603. Previous studies demonstrated that lncRNAs may function by controlling the transcriptional regulation of neighboring coding genes [65, 66]. Identifying differentially expressed nearby coding mRNAs may enhance our understanding of the function of lncRNAs in MSCs. However, further studies must be performed to investigate that hypothesis.

## 5. Conclusion

This study provided comprehensive profiles of mRNA and lncRNA expression in PDLSCs and BMSCs, two tissue-derived MSCs. In addition, potential regulatory mechanisms were identified with bioinformatics analyses. Although more studies are required to demonstrate the precise role and mechanisms of these lncRNAs and mRNAs, the genomic data we identified with microarray analyses may increase our understanding of MSC biology.

## Supplementary Material

Expression profiles of lncRNA and mRNA between bone marrow stem cells (BMSCs) and periodontal ligament stem cells (PDLSCs) were investigated with lncRNA microarray assays and bioinformatics analysis. In PDLSCs, 970 lncRNAs that were significantly differentially expressed compared to BMSCs (Supplementary Table 1). Furthermore, 1,578 mRNAs were differentially expressed in the PDLSCs and BMSCs (Supplementary Table 2). For bioinformatics analysis, the results of the GO analysis showed which important functions were involved with the differentially expressed genes, including the top upregulated and downregulated GO functions (upGOs and downGOs) (Supplementary Figures 1 and 2). Then based on the KEGG database, there were identified 67 pathways that showed significant differences due to differential gene expression (Supplementary Figures 3 and 4), which play key roles in the different core epigenetic mechanisms of PDLSCs and BMSCs. To further investigate the global network, differentially expressed genes were identified by a Signal-net analysis (Supplementary Table 3). Finally, coding-noncoding gene coexpression (CNC) networks were used to implicate the interregulation of lncRNAs and mRNAs in the different molecular mechanisms of PDLSCs and BMSCs (Supplementary Figures 5 and 6).

## Figures and Tables

**Table 1:** Primer sequences used in the real-time RT-PCR validation of microarray analyses.

Target gene symbol	Primer sequences (5′-3′)	Target size (bp)	*T*_*m*_ (°C)
NR_045555-F	GTTGCAAGGAAACCTTTGGA	96	60
NR_045555-R	CTGCATGCTGTTGACCTTGT		
NR_027621-F	CTGCGTGGATTGCTACAAGA	102	60
NR_027621-R	CCTTCATAGGCCACCACACT		
XR_111050-F	ATGGCCAGTTCGTTTCTCAC		60
XR_111050-R	AAGACACGTCCTTGGTTTGG		
NR_037595-F	CCCTGTGCAAGAGCACATAA		60
NR_037595-R	TGCCAGCTCATACAAGATGC		
NR_033651-F	CCCCTTGGTATTCTCCCAAT		60
NR_033651-R	CAGCCTTTTGTTGGGTGTTT		
NR_037182-F	CTTCTGCAGGAGGAATCCAG		60
NR_037182-R	TCCCAGTTTTTGGTGACTCC		
GAPDH-F	CGGACCAATACGACCAAATCCG	83	60
GAPDH-R	AGCCACATCGCTCAGACACC		
HOXA9-F	CGGTTATGGCATTAAACCTGAACCG	67	60
HOXA9-R	GTGAGTGTCAAGCGTGGGACAG		
HOXC8-F	CGGTAAGTTCCAAGGTCTGATACCG	99	60
HOXC8-R	CGTCTCCCAGCCTCATGTTTC		
WNT2B-F	CTTTCCTTTGCACCAGCTTC	52	60
WNT2B-R	TACCCTTCCTCTTGCACACC		
BARX1-F	CGCTTCGAGAAGCAGAAGTA	111	60
BARX1-R	CTTCATCCTCCGATTCTGGT		
IGFBP5-F	GCACCTGAGATGAGACAGGA	139	60
IGFBP5-R	TGTAGAATCCTTTGCGGTCA		
S100A4-F	GTACTTGGTGTCCACCTTCCACAAGTAC		60
S100A4-R	CCGGGTCAGCAGCTCCTTTAG		

**Table 2:** Seventeen genes identified in the PDLSC CNC network with high degrees of connectivity and clustering coefficients (degree ≥59, clustering coefficient ≥0.6).

Gene symbol	Description	Clustering coefficient	Degree	Style	Type
FLNB	Filamin B, beta	0.67332309	63	Down	mRNA
PTTG1	Pituitary tumor-transforming factor-1	0.72021858	61	Up	mRNA
GNG11	Guanine nucleotide binding protein (G protein), gamma 11	0.72021858	61	Up	mRNA
IGF1R	Insulin-like growth factor-1 receptor	0.67431694	61	Up	mRNA
ITGA2	Integrin, alpha 2 (CD49B, alpha 2 subunit of VLA-2 receptor)	0.66994536	61	Up	mRNA
ENPP1	Ectonucleotide pyrophosphatase/phosphodiesterase 1	0.73107345	60	Down	mRNA
CDC20	Cell division cycle 20 homolog (*S. cerevisiae*)	0.70734463	60	Up	mRNA
COL11A1	Collagen, type XI, alpha 1	0.70734463	60	Down	mRNA
DBF4	DBF4 homolog (*S. cerevisiae) *	0.70734463	60	Up	mRNA
NR_040093	gi∣338968843∣ref∣NR_040093.1∣	0.76446523	59	Down	lncRNA
XR_112964	gi∣310115154∣ref∣XR_112964.1∣	0.74868498	59	Down	lncRNA
XR_108725	gi∣310119896∣ref∣XR_108725.1∣	0.74868498	59	Down	lncRNA
XR_110624	gi∣310118206∣ref∣XR_110624.1∣	0.74868498	59	Down	lncRNA
CCNB2	Cyclin B2	0.68322618	59	Up	mRNA
GSTM5	Glutathione S-transferase mu 5	0.68322618	59	Up	mRNA
HLA-DMA	Major histocompatibility complex, class II, DM alpha	0.68322618	59	Down	mRNA
WASF3	WAS protein family, member 3	0.68264173	59	Down	mRNA

**Table 3:** Twenty genes identified in the BMSC CNC network with high degrees of connectivity and clustering coefficients (degree ≥29, clustering coefficient ≥0.7).

Gene symbol	Description	Clustering coefficient	Degree	Style	Type
CXCL12	Chemokine (C-X-C motif) ligand 12	0.75568182	33	Up	mRNA
PRIM1	Primase, DNA, polypeptide 1 (49 kDa)	0.87931034	29	Up	mRNA
LIFR	Leukemia inhibitory factor receptor alpha	0.87931034	29	Down	mRNA
MAD2L1	MAD2 mitotic arrest deficient-like 1 (yeast)	0.87931034	29	Up	mRNA
TGFBR1	Transforming growth factor, beta receptor 1	0.87931034	29	Down	mRNA
PARP1	Poly(ADP-ribose) polymerase 1	0.87931034	29	Up	mRNA
FGF5	Fibroblast growth factor-5	0.87931034	29	Up	mRNA
CCNE2	Cyclin E2	0.87931034	29	Up	mRNA
TTK	TTK protein kinase	0.87931034	29	Up	mRNA
RBL1	Retinoblastoma-like 1 (p107)	0.87931034	29	Up	mRNA
POLE2	Polymerase (DNA directed), epsilon 2 (p59 subunit)	0.87931034	29	Up	mRNA
CDK1	Cyclin-dependent kinase 1	0.87931034	29	Up	mRNA
MCM3	Minichromosome maintenance complex component 3	0.87931034	29	Up	mRNA
CDK2	Cyclin-dependent kinase 2	0.87931034	29	Up	mRNA
BMPR1B	Bone morphogenetic protein receptor, type IB	0.87931034	29	Down	mRNA
HIST1H2BO	Histone cluster 1, H2bo	0.80295567	29	Up	mRNA
F10	Coagulation factor X	0.80295567	29	Up	mRNA
BDKRB1	Bradykinin receptor B1	0.80295567	29	Up	mRNA
GSTM5	Glutathione S-transferase mu 5	0.80295567	29	Up	mRNA
PRPH2	Peripherin 2 (retinal degeneration, slow)	0.80295567	29	Down	mRNA

**Table 4:** Sixteen genes with different pathway connectivities (identified with Diffk) in PDLSCs and BMSCs (∣Diffk∣  ≥  0.75).

Gene symbol	Description	Style	Type	∣Diffk∣
CXCL12	Chemokine (C-X-C motif) ligand 12	Up	mRNA	1
ITGA2	Integrin, alpha 2 (CD49B, alpha 2 subunit of VLA-2 receptor)	Up	mRNA	0.968254
CDC20	Cell division cycle 20 homolog (*S. cerevisiae*)	Up	mRNA	0.952381
WASF3	WAS protein family, member 3	Down	mRNA	0.9365079
CAMK4	Calcium/calmodulin-dependent protein kinase IV	Up	mRNA	0.8571429
SEMA3C	Sema domain, immunoglobulin domain (Ig), short basic domain, secreted, (semaphorin) 3C	Down	mRNA	0.8571429
CCNA2	Cyclin A2	Up	mRNA	0.8253968
POLA1	Polymerase (DNA directed), alpha 1, catalytic subunit	Up	mRNA	0.8253968
FR020479	AB209345, AC006512, U47924	Down	lncRNA	0.7878788
FR191603	AJ609445, AK128061, AP001273	Up	lncRNA	0.7532468
SLK	STE20-like kinase	Up	mRNA	0.7460317
PDGFA	Platelet-derived growth factor alpha polypeptide	Down	mRNA	0.7388167
PTK2	PTK2 protein tyrosine kinase 2	Up	mRNA	0.7142857
ENPP1	Ectonucleotide pyrophosphatase/phosphodiesterase 1	Down	mRNA	0.7099567
PRKCE	Protein kinase C, epsilon	Up	mRNA	0.7056277
BDKRB1	Bradykinin receptor B1	Up	mRNA	0.7041847
