# Supplementary material for: Comparison of Long Noncoding RNA and mRNA Expression Profiles in Mesenchymal Stem Cells Derived from Human Periodontal Ligament and Bone Marrow
Source: Biomed Res Int. 2014 Mar 27;2014:317853. doi: 10.1155/2014/317853 (PMC3985196; doi:10.1155/2014/317853)
Supplement: Supplementary file 1 — Expression profiles of lncRNA and mRNA between bone marrow stem cells (BMSCs) and periodontal ligament stem cells (PDLSCs) were investigated with lncRNA microarray assays and bioinformatics analysis. In PDLSCs, 970 lncRNAs that were significantly differentially expressed compared to BMSCs (Supplementary Table 1). Furthermore, 1,578 mRNAs were differentially expressed in the PDLSCs and BMSCs (Supplementary Table 2). For bioinformatics analysis, the results of the GO analysis showed which important functions were involved with the differentially expressed genes, including the top upregulated and downregulated GO functions (upGOs and downGOs) (Supplementary Figures 1 and 2). Then based on the KEGG database, there were identified 67 pathways that showed significant differences due to differential gene expression (Supplementary Figures 3 and 4), which play key roles in the different core epigenetic mechanisms of PDLSCs and BMSCs. To further investigate the global network, differentially expressed genes were identified by a Signal-net analysis (Supplementary Table 3). Finally, coding-noncoding gene coexpression (CNC) networks were used to implicate the interregulation of lncRNAs and mRNAs in the different molecular mechanisms of PDLSCs and BMSCs (Supplementary Figures 5 and 6). [file 317853.f1.pdf]

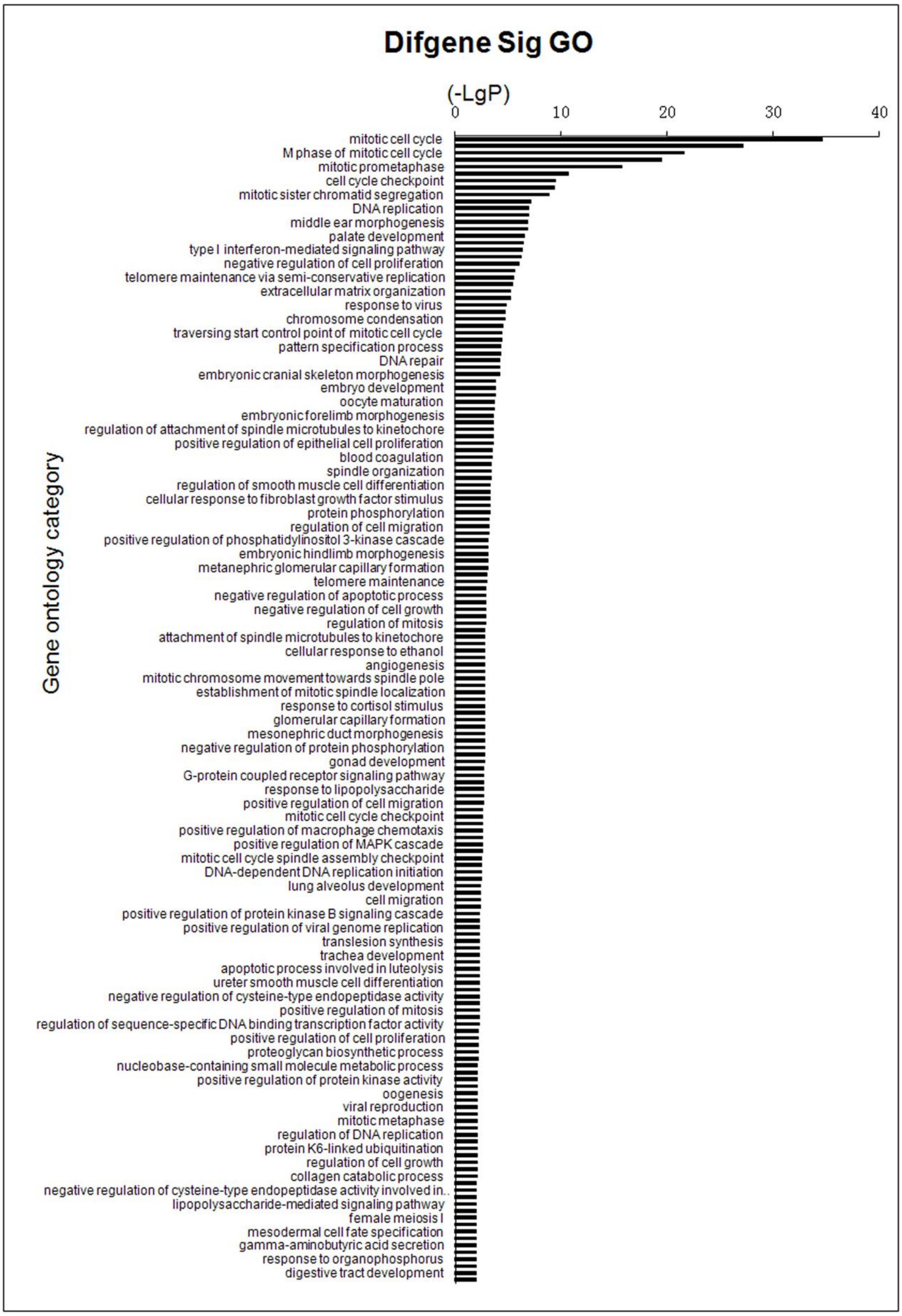

Supplementary Fig. 1. Significant upGOs of differentially expressed genes in PDLSCs compared with BMSCs. The y axis shows the GO category and the x axis shows the -LgP. A larger -LgP indicated a smaller P value for the difference between PDLSCs and BMSCs.

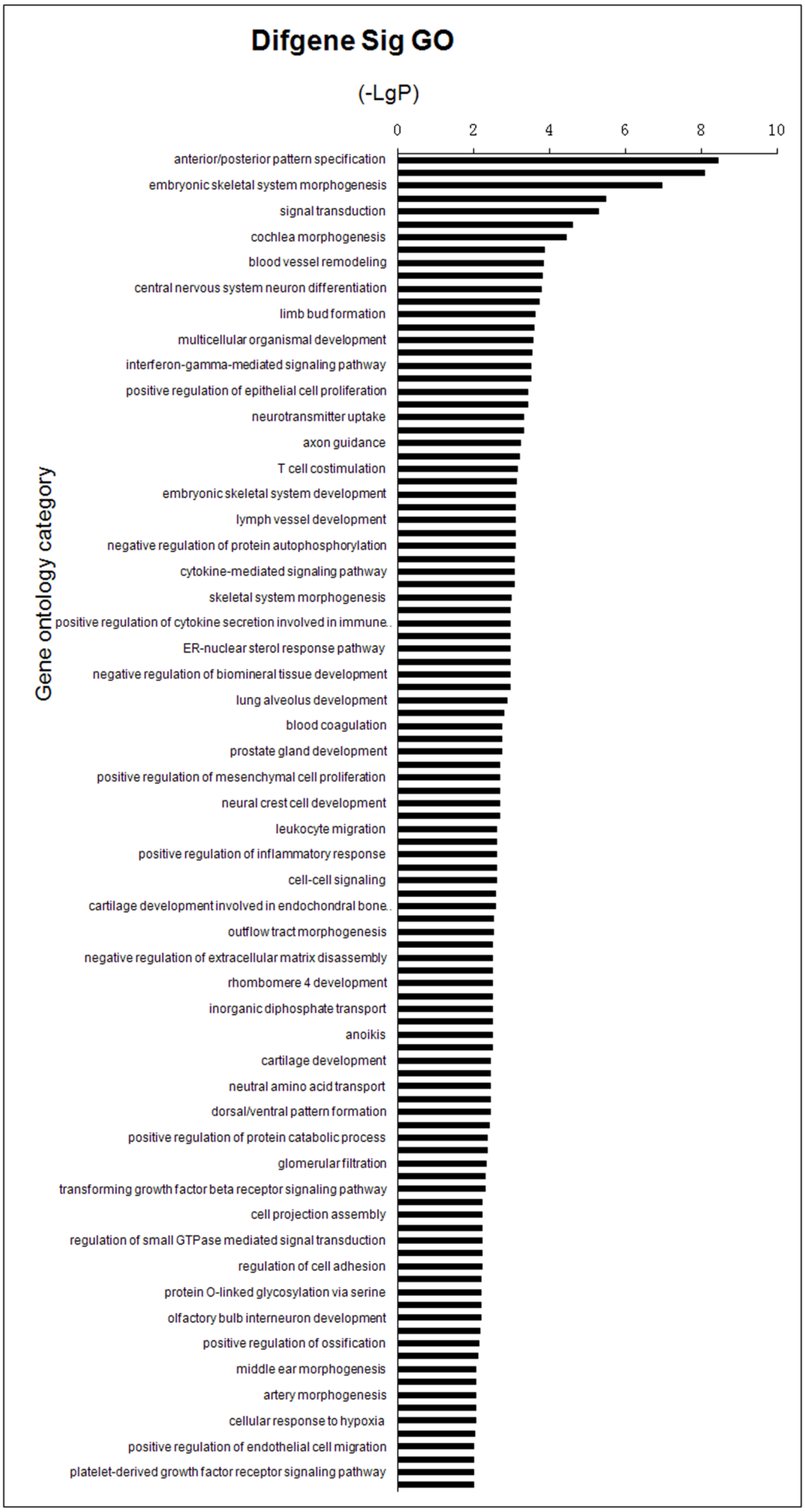

Supplementary Fig. 2. Significant downGOs of differentially expressed genes in PDLSCs compared with BMSCs. The y axis shows the GO category and the x axis shows the -LgP. A larger -LgP indicated a smaller P value for the difference between PDLSCs and BMSCs.

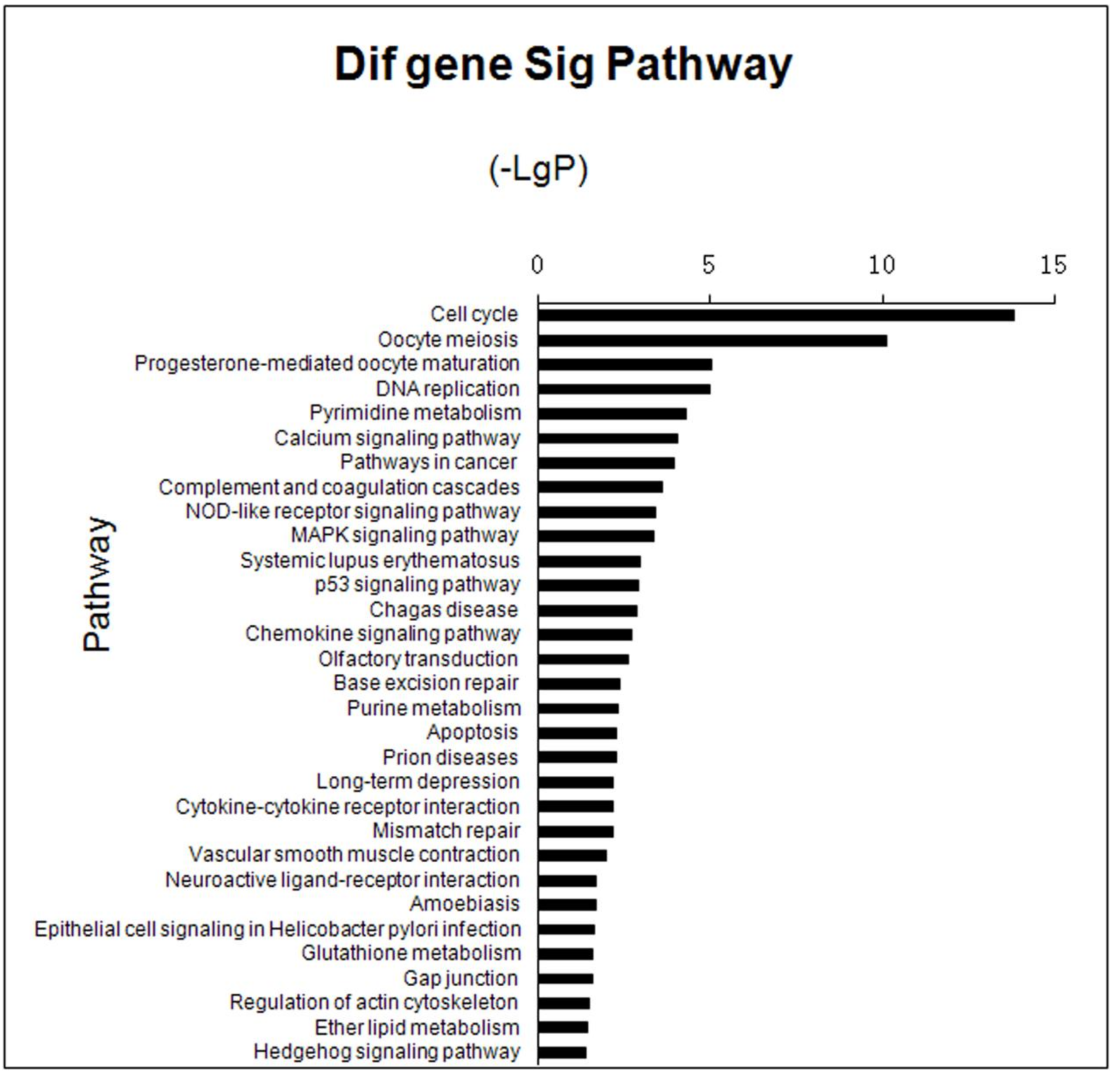

Supplementary Fig. 3. Significantly upregulated pathways in PDLSCs compared with BMSCs. Based on the KEGG database, pathways with $P<0.05$ and $FDR<0.05$ were identified as pathways with significantly different behaviors in the two MSC types. The y axis shows significantly different pathways and the x axis shows the -LgP. A larger -LgP indicated a smaller P value for the difference between PDLSCs and BMSCs.

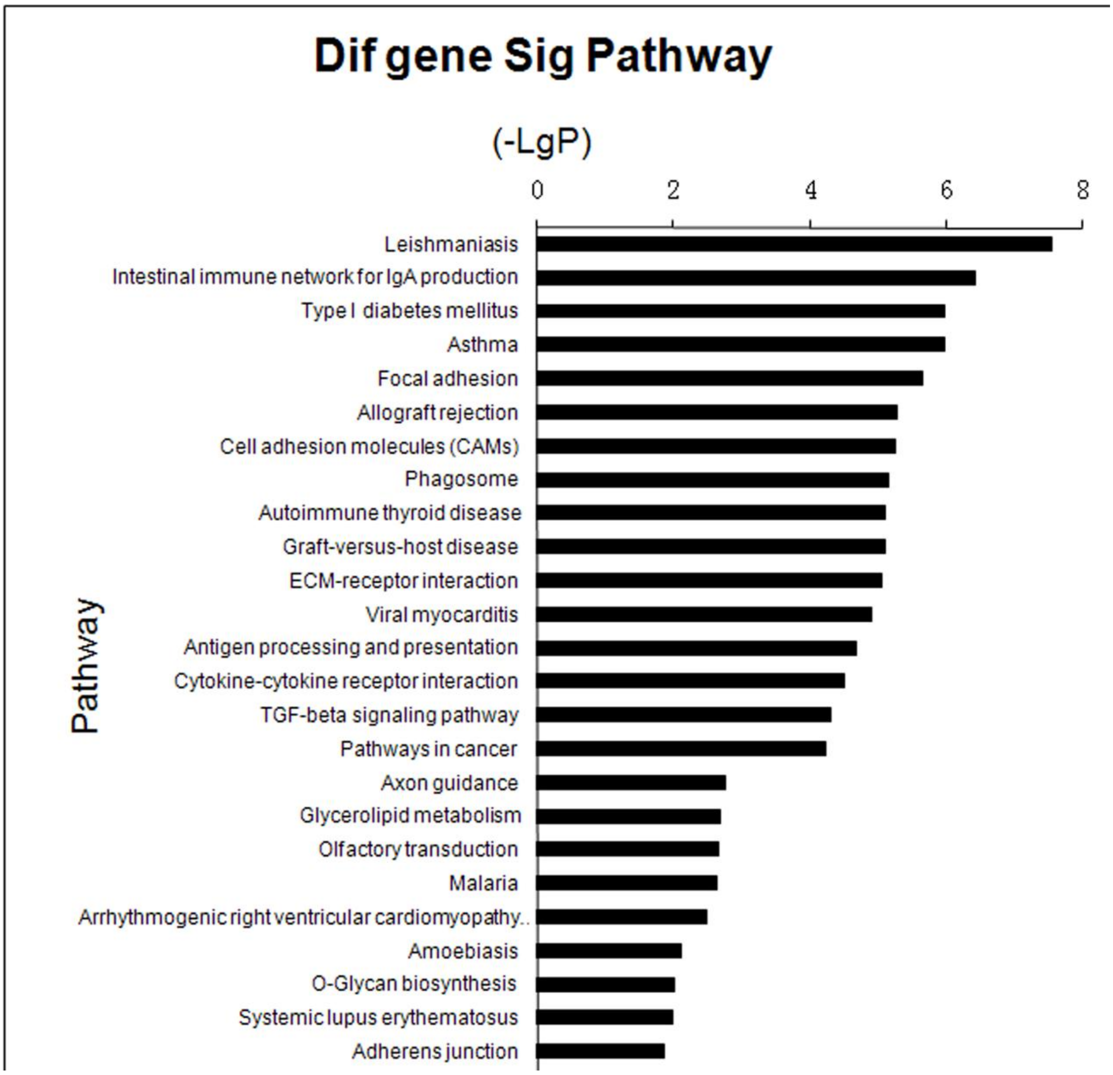

Supplementary Fig.4. Significantly downregulated pathways in PDLSCs compared with BMSCs. Based on the KEGG database, pathways with $P<0.05$ and $FDR<0.05$ were identified as pathways with significantly different behaviors in the two MSC types. The y axis shows significantly changed pathways and the x axis shows the -LgP. A larger -LgP indicated a smaller P value for the difference between PDLSCs and BMSCs.

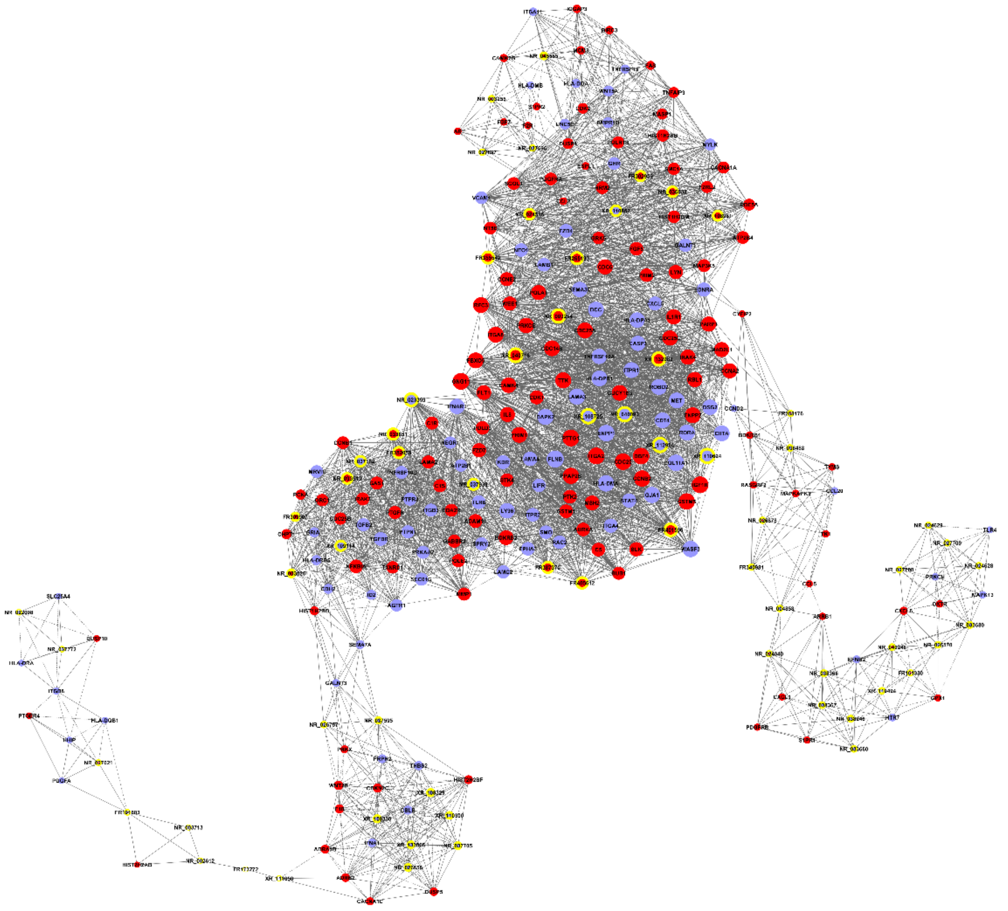

Supplemental Fig. 5. Gene co-expression network in PDLSCs (CNC). Circles represent up-regulated (red) genes and down-regulated (blue) genes in PDLSCs. The lines represent the regulatory relationships between genes (solid lines represent positive correlations, dotted lines represent negative correlations). The circle size represents the degree of centrality.

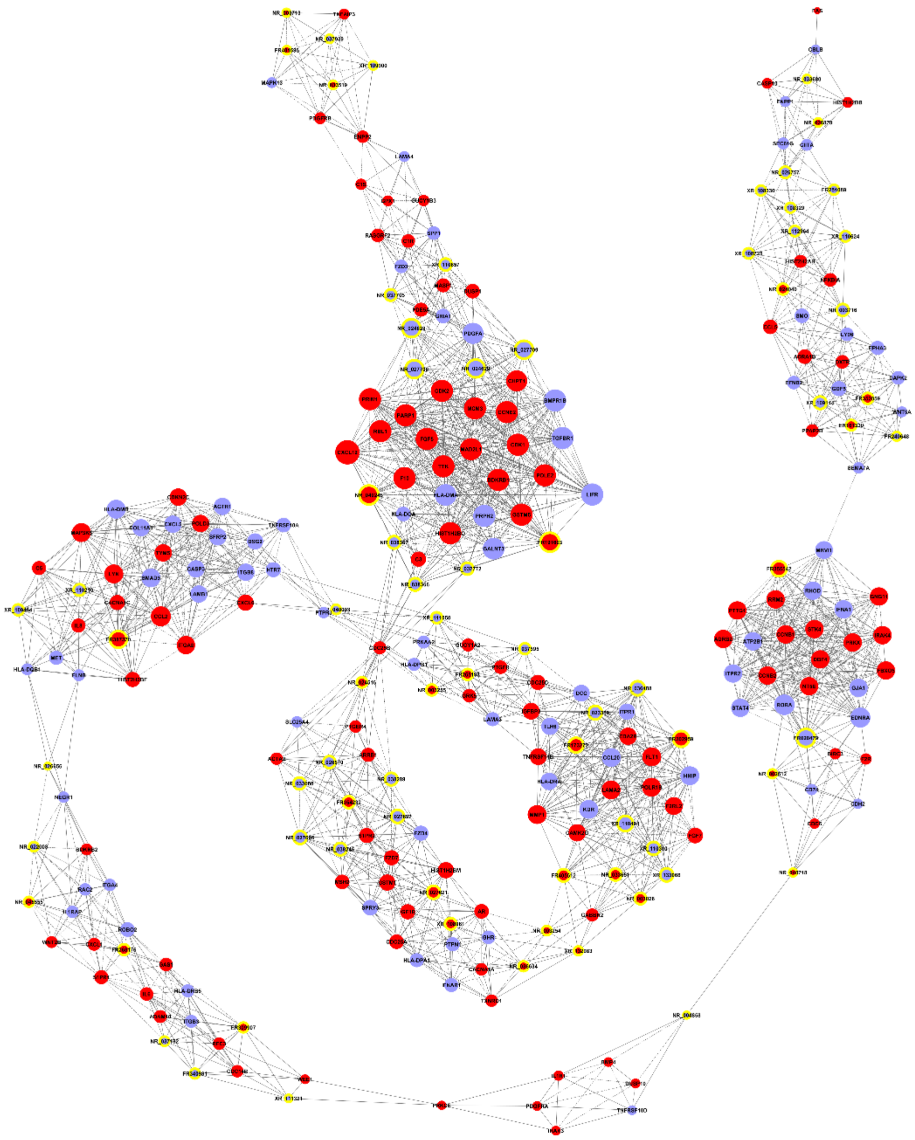

Supplemental Fig. 6. Gene co-expression network in BMSCs (CNC). Circles represent up-regulated (red) genes and down-regulated (blue) genes in BMSCs). The lines represent the regulatory relationships between genes (solid lines represent positive correlations, dotted lines represent negative correlations). The circle size represents the degree of centrality.

Supplementary table 1.differentially expressed lncRNA in BMSCs and PDLSCs

**ProbeSet**	**p-value**	**FDR**	**PDLSCs/BMSCs**	**style**	**database_ID/chromosome_locati**
NC00589FR	1.00E-07	1.00E-07	33.56	up	FR401596
ASK01163NTNR	1.00E-07	1.00E-07	23.38	up	NR_003254
ASK05428REF_PAX9_AS001	1.00E-07	1.00E-07	11.28	up	chr14:37146567-37146943
TI23418REF_MSX1_AS001	1.00E-07	1.00E-07	7.36	up	chr4:4863421-4863582
TI16343REF_FBLN1_AS008	6.00E-07	6.34E-05	6.62	up	chr22:45946874-45946968
ASK03099REF	4.50E-06	0.00024	5.48	up	ENST00000487335
ASK00935NTNR	1.90E-06	0.00015	5.16	up	NR_045555
TI16101REF_TNS1_AS006	2.40E-06	0.00016	4.75	up	chr2:218667055-218667168
ASK01183NTNR	1.00E-07	1.00E-07	4.7	up	NR_024040
ASK07241REF_RASGRF2_AS00	2.00E-07	2.78E-05	4.57	up	chr5:80409226-80409719
ASK05258REF_XAF1_AS002	1.00E-07	1.00E-07	4.54	up	chr17:6676712-6678909
ASK05799REF_FBN2_AS001	1.00E-07	1.65E-05	4.3	up	chr5:127594099-127594489
TI20852REF_FST_AS001	2.10E-06	0.00016	4.27	up	chr5:52782287-52782418
ASK00744NTNR	5.00E-07	5.50E-05	4.08	up	NR_033651
TI16101REF_TNS1_AS026	9.60E-06	0.0004	3.96	up	chr2:218665510-218665611
TI19423REF_RASGRF2_AS001	2.00E-07	2.78E-05	3.77	up	chr5:80522339-80522497
ASK04300REF	8.80E-06	0.0004	3.74	up	ENST00000513588
TI16108REF_PIEZO2_AS003	2.00E-07	2.78E-05	3.7	up	chr18:10784823-10784959
TI16108REF_PIEZO2_AS021	7.00E-07	6.60E-05	3.29	up	chr18:10770160-10770292
ASK04298REF	1.14E-05	0.00045	3.08	up	ENST00000505968
TI24039REF_TRIM14_AS001	1.40E-06	0.00011	3.06	up	chr9:100846340-100846447
TI16099REF_KAZN_AS006	5.40E-05	0.00123	2.87	up	chr1:15393201-15393360
ASK06987REF_CTSK_AS001	3.20E-06	0.00018	2.85	up	chr1:150768837-150769080
ASK05728REF_PDGFRA_AS001	2.40E-06	0.00016	2.84	up	chr4:55163473-55163767
TI16218REF_SEC31A_AS019	0.00011	0.00209	2.59	up	chr4:83817920-83818014
TI20059REF_SIX1_AS002	0.000309	0.0041	2.58	up	chr14:61114896-61115003
TI16101REF_TNS1_AS019	1.52E-05	0.00056	2.57	up	chr2:218665096-218665213
ASK05232REF_SYNGR2_AS002	2.27E-05	0.00071	2.53	up	chr17:76167841-76168029
TI16610REF_ELFN1_AS009	6.50E-06	0.00032	2.49	up	chr7:1786695-1786796
TI16517REF_TBC1D8_AS001	3.55E-05	0.00095	2.48	up	chr2:101648947-101649194
TI19633REF_MR1_AS002	1.67E-05	0.00059	2.45	up	chr1:181024988-181025086
TI17807REF_LPCAT4_AS003	3.65E-05	0.00096	2.41	up	chr15:34653205-34653354
NC00521FR	3.88E-05	0.00099	2.41	up	FR354232
TI17419REF_SPATA13_AS001	0.000187	0.00291	2.4	up	chr13:24879606-24879708
TI16389REF_SPTBN1_AS003	1.53E-05	0.00056	2.38	up	chr2:54785399-54785497
TI16036REF_EIF2B5_AS041	0.000287	0.00387	2.37	up	chr3:183861429-183861694
TI17754REF_WNT5B_AS004	0.00168	0.0137	2.37	up	chr12:1751844-1751946
TI16307REF_SNX5_AS009	3.50E-05	0.00094	2.34	up	chr20:17923923-17924153
TI16762REF_FAM107B_AS006	7.03E-05	0.00151	2.33	up	chr10:14615006-14615097
TI20546REF_HJURP_AS002	0.000223	0.00321	2.33	up	chr2:234759105-234759263
TI17419REF_SPATA13_AS006	0.007152	0.0362	2.33	up	chr13:24880166-24880273
TI22054REF_PAX9_AS001	2.50E-05	0.00076	2.3	up	chr14:37132911-37133035
ASK02554REF	2.12E-05	0.00068	2.27	up	ENST00000480416
TI14647REF	0.001173	0.0105	2.26	up	ENST00000496878
TI16101REF_TNS1_AS012	5.60E-06	0.00028	2.25	up	chr2:218666131-218666254
TI17613REF_PCBD2_AS004	0.000677	0.00712	2.25	up	chr5:134259034-134259191
ASK05085REF_TJP2_AS002	0.000484	0.0056	2.24	up	chr9:71869318-71870081
TI16517REF_TBC1D8_AS002	0.000931	0.00887	2.23	up	chr2:101649872-101649993
TI17698REF_SLFNL1_AS005	0.0011	0.01	2.2	up	chr1:41484975-41485114
TI17138REF_CSDA_AS007	5.72E-05	0.00128	2.18	up	chr12:10864796-10865241
TI16559REF_CPT1A_AS009	0.001547	0.0128	2.18	up	chr11:68524134-68524253
TI21517REF_RPS24_AS001	0.010599	0.0466	2.18	up	chr10:79794060-79794144

ASK05085REF_TJP2_AS001	0.000734	0.0074	2.15	up	chr9:71864154-71869263
TI20047REF_IRF9_AS001	0.003951	0.0243	2.14	up	chr14:24633344-24633572
TI17686REF_PRDX1_AS004	0.000816	0.008	2.13	up	chr1:45982922-45983045
TI16389REF_SPTBN1_AS002	0.000101	0.00203	2.12	up	chr2:54891445-54891577
ASK01038NTNR	0.006637	0.0345	2.12	up	XR_132983
ASK02328REF	2.71E-05	0.0008	2.09	up	ENST00000537607
TI19255REF_ODC1_AS003	0.000737	0.0074	2.09	up	chr2:10587563-10587667
TI16517REF_TBC1D8_AS007	7.36E-05	0.00155	2.08	up	chr2:101647532-101647713
TI16360REF_NFIA_AS003	0.000214	0.00316	2.05	up	chr1:61926827-61926930
TI16913REF_ATG16L2_AS007	0.002812	0.0191	2.05	up	chr11:72534354-72534608
TI16327REF_KIF22_AS013	0.000221	0.0032	2.04	up	chr16:29815678-29815846
NC00438FR	0.000272	0.0037	2.04	up	FR302959
TI17686REF_PRDX1_AS001	0.012564	0.0514	2.03	up	chr1:45982234-45982371
TI16517REF_TBC1D8_AS010	0.000225	0.00321	2.02	up	chr2:101706122-101706221
ASK04880REF_OPN1SW_AS002	0.000154	0.00258	2	up	chr7:128412519-128413088
TI16218REF_SEC31A_AS001	0.00093	0.00887	2	up	chr4:83818062-83818254
TI16559REF_CPT1A_AS005	0.001376	0.0118	2	up	chr11:68522463-68522618
ASK05274REF_RNASET2_AS00[illegible]	0.000858	0.00829	1.99	up	chr6:167369673-167370041
ASK04913REF_ADAMTS15_AS[illegible]	0.000136	0.00236	1.97	up	chr11:130317889-130319793
NC00599FR	0.00069	0.00718	1.97	up	FR405612
ASK00817NTNR	0.000324	0.00421	1.96	up	NR_027621
TI17807REF_LPCAT4_AS005	0.000175	0.00275	1.95	up	chr15:34653486-34653599
TI18282REF_OSGEP_AS002	0.010563	0.0466	1.95	up	chr14:20920701-20920839
ASK05822REF_MAPKAPK3_AS[illegible]	6.56E-05	0.00142	1.94	up	chr3:50686286-50686683
ASK06516REF_ZFPM2_AS001	0.00037	0.00458	1.94	up	chr8:106810784-106811176
TI16640REF_EIF4G2_AS001	0.000669	0.00706	1.94	up	chr11:10828588-10828687
ASK01164NTNR	0.001581	0.013	1.94	up	XR_108961
ASK01032NTNR	0.000134	0.00235	1.93	up	NR_033519
TI19599REF_RPS4X_AS002	0.000964	0.00911	1.93	up	chrX:71494100-71494321
TI16099REF_KAZN_AS016	0.005952	0.0323	1.93	up	chr1:15393432-15393541
ASK04913REF_ADAMTS15_AS[illegible]	0.000199	0.00301	1.91	up	chr11:130318587-130318953
TI16146REF_FHOD3_AS016	0.000521	0.00596	1.91	up	chr18:34356032-34356139
TI20863REF_DEPDC1B_AS001	0.003045	0.0202	1.91	up	chr5:59896235-59896331
TI19599REF_RPS4X_AS001	0.007977	0.0388	1.91	up	chrX:71493891-71494045
TI02113NTNR	0.005469	0.0308	1.9	up	NR_003026
TI20047REF_IRF9_AS002	0.000155	0.00258	1.89	up	chr14:24634421-24634624
ASK00705NTNR	0.000271	0.0037	1.88	up	NR_040248
TI20125REF_RPL4_AS001	0.000828	0.0081	1.88	up	chr15:66793465-66793683
ASK06477REF_DGUOK_AS001	0.006365	0.0337	1.88	up	chr2:74185251-74208448
TI09189REF	0.000169	0.00269	1.87	up	ENST00000563240
ASK07329REF_PRKCE_AS001	0.000189	0.00292	1.87	up	chr2:46414063-46414472
ASK00927NTNR	0.001124	0.0101	1.87	up	NR_002612
NC00236FR	0.001874	0.0146	1.87	up	FR161330
TI17138REF_CSDA_AS003	0.002086	0.0156	1.87	up	chr12:10865708-10865806
ASK05545REF_DHCR24_AS001	7.97E-05	0.00166	1.86	up	chr1:55352550-55352777
TI11541REF	0.000106	0.00203	1.85	up	ENST00000559863
TI19693REF_STMN1_AS001	0.000122	0.00222	1.85	up	chr1:26229659-26229760
TI19372REF_LSG1_AS001	0.000326	0.00422	1.85	up	chr3:194371514-194371602
TI20214REF_NQO1_AS001	0.007139	0.0362	1.85	up	chr16:69751402-69751482
TI15124REF	0.009272	0.0427	1.85	up	ENST00000482261
TI19861REF_IFITM2_AS002	0.000159	0.00258	1.84	up	chr11:314409-314506
ASK06414REF_SLITRK2_AS001	0.00218	0.0159	1.84	up	chrX:144903929-144963163
TI16829REF_C20orf111_AS008	0.002262	0.0162	1.84	up	chr20:42837344-42837545
TI13444REF	0.003	0.02	1.84	up	ENST00000552548
TI20585REF_ASIP_AS001	0.005092	0.0293	1.84	up	chr20:32803699-32803793

TI16936REF_ZNF219_AS004	0.016262	0.0616	1.84	up	chr14:21565242-21565327
TI16120REF_DGKD_AS024	0.021632	0.0752	1.84	up	chr2:234371411-234371530
TI18602REF_TNIP1_AS003	0.000103	0.00203	1.83	up	chr5:150413890-150414042
TI17696REF_SLC30A1_AS005	0.000442	0.00526	1.83	up	chr1:211746039-211746133
ASK05012REF_TFRC_AS002	0.000624	0.00672	1.83	up	chr3:195776363-195776662
TI16435REF_ZFHX3_AS005	0.006046	0.0325	1.83	up	chr16:73080301-73080426
TI17527REF_LTBP1_AS005	0.008931	0.0417	1.83	up	chr2:33468124-33468259
TI17774REF_SMARCC2_AS001	0.000385	0.00475	1.82	up	chr12:56572488-56572589
TI17622REF_SHROOM1_AS005	0.000695	0.00718	1.82	up	chr5:132163839-132163966
TI16445REF_THADA_AS012	0.027233	0.0884	1.82	up	chr2:43449704-43449798
ASK04743REF_THADA_AS003	0.000224	0.00321	1.81	up	chr2:43451447-43451663
TI19441REF_DUSP1_AS003	0.00111	0.01	1.81	up	chr5:172196994-172197149
TI17025REF_EIF4A2_AS002	0.002214	0.016	1.81	up	chr3:186502579-186502744
ASK00777NTNR	0.009109	0.0422	1.81	up	NR_003255
TI16880REF_PTBP2_AS008	0.014883	0.0589	1.81	up	chr1:97262663-97262770
TI13458REF	0.026846	0.0878	1.81	up	ENST00000474791
TI16389REF_SPTBN1_AS005	0.001559	0.0129	1.8	up	chr2:54891296-54891418
TI21406REF_DUSP10_AS001	0.002351	0.0167	1.8	up	chr1:221914262-221914425
ASK04606REF	0.000346	0.00442	1.79	up	ENST00000470972
TI16389REF_SPTBN1_AS007	0.000537	0.00605	1.79	up	chr2:54889553-54889671
TI17374REF_VCL_AS002	0.000636	0.00683	1.79	up	chr10:75760815-75760941
TI20632REF_ETS2_AS001	0.000955	0.00907	1.79	up	chr21:40181502-40181743
TI16131REF_EEF1D_AS018	0.001772	0.0141	1.79	up	chr8:144678373-144678643
TI17836REF_RP11-1035H13.3_A	0.003192	0.0209	1.79	up	chr16:18807571-18807710
TI16687REF_TGIF1_AS001	0.004903	0.0286	1.79	up	chr18:3451196-3451315
TI16389REF_SPTBN1_AS013	0.047046	0.131	1.79	up	chr2:54890259-54890410
TI16218REF_SEC31A_AS015	0.006321	0.0335	1.78	up	chr4:83818496-83818601
ASK07289REF_NDST1_AS001	0.008315	0.0396	1.78	up	chr5:149919122-149919271
ASK04945REF_CDKN2C_AS002	0.013673	0.0551	1.78	up	chr1:51439961-51440281
TI19441REF_DUSP1_AS002	0.000689	0.00718	1.77	up	chr5:172196150-172196259
TI19051REF_TAF1C_AS003	0.001628	0.0134	1.77	up	chr16:84215677-84215803
TI17492REF_TRIM25_AS002	0.00192	0.0149	1.77	up	chr17:54974989-54975093
TI18223REF_NCAPD3_AS004	0.023488	0.0795	1.77	up	chr11:134024802-134024909
TI17362REF_CSF1_AS002	0.000197	0.00301	1.76	up	chr1:110472988-110473216
TI16920REF_TNKS1BP1_AS004	0.001655	0.0136	1.76	up	chr11:57074597-57074720
TI16559REF_CPT1A_AS004	0.001817	0.0144	1.76	up	chr11:68524334-68524453
TI01378NTNR	0.001943	0.0149	1.76	up	NR_036634
TI17505REF_PTBP1_AS004	0.002169	0.0159	1.76	up	chr19:807903-808083
TI18660REF_FAM120A_AS001	0.003537	0.0224	1.76	up	chr9:96308812-96308961
TI11628REF	0.005566	0.0309	1.76	up	ENST00000563473
TI17532REF_EPAS1_AS005	0.012495	0.0513	1.76	up	chr2:46608094-46608293
TI17324REF_ZFP41_AS007	0.013553	0.0547	1.76	up	chr8:144355848-144356005
TI19721REF_AHCTF1_AS001	0.000401	0.00487	1.75	up	chr1:247087242-247087390
TI20920REF_FAM46A_AS002	0.001191	0.0106	1.75	up	chr6:82460499-82460632
TI19855REF_BANF1_AS002	0.002039	0.0153	1.75	up	chr11:65770390-65770497
ASK04974REF_QPCT_AS002	0.007004	0.0357	1.75	up	chr2:37599831-37599977
TI16437REF_ADCY9_AS013	0.011905	0.0502	1.75	up	chr16:4155957-4156056
TI16334REF_RP11-159D12.5_AS	0.012328	0.0513	1.75	up	chr17:56067505-56067640
TI17841REF_RPAIN_AS005	0.015236	0.0593	1.75	up	chr17:5335415-5335506
TI16362REF_PABPC4_AS007	0.001116	0.0101	1.74	up	chr1:40034945-40035125
TI16729REF_RP11-514O12.4_AS	0.003717	0.0233	1.74	up	chr6:167368983-167369110
TI17383REF_TM9SF3_AS006	0.006865	0.0353	1.74	up	chr10:98278213-98278311
TI16178REF_ASPSCR1_AS017	0.021163	0.0741	1.74	up	chr17:79971816-79971909
TI16287REF_FAM53B_AS007	0.027713	0.0892	1.74	up	chr10:126353820-126353967
TI18602REF_TNIP1_AS004	0.000323	0.00421	1.73	up	chr5:150413595-150413829

TI21125REF_SMC1A_AS001	0.001239	0.0109	1.73	up	chrX:53426775-53426880
ASK04880REF_OPN1SW_AS001	0.00142	0.0121	1.73	up	chr7:128412857-128413017
TI17525REF_CFLAR_AS002	0.001664	0.0136	1.73	up	chr2:202028225-202028335
TI22106REF_FAM63B_AS001	0.00194	0.0149	1.73	up	chr15:59152108-59152233
TI18554REF_UBP1_AS002	0.003801	0.0237	1.73	up	chr3:33444752-33444865
TI17649REF_RALGPS1_AS004	0.013119	0.0534	1.73	up	chr9:129942507-129942603
TI16389REF_SPTBN1_AS014	0.016049	0.0611	1.73	up	chr2:54890072-54890202
TI19903REF_BDNF_AS001	0.000434	0.0052	1.72	up	chr11:27720487-27720580
TI17627REF_EEF1A1_AS001	0.000563	0.00622	1.72	up	chr6:74229279-74229381
ASK04849REF_S100A4_AS001	0.000744	0.00742	1.72	up	chr1:153518289-153521228
TI17277REF_PVRL3_AS002	0.000856	0.00829	1.72	up	chr3:110916506-110916598
ASK06744REF_TOR1B_AS001	0.000985	0.00916	1.72	up	chr9:132572829-132573279
TI22199REF_PDPR_AS001	0.001968	0.015	1.72	up	chr16:70152834-70152991
TI17531REF_IL1R1_AS002	0.002183	0.0159	1.72	up	chr2:102764137-102764231
TI01740NTNR	0.002372	0.0168	1.72	up	NR_003713
TI20465REF_BZW1_AS001	0.002538	0.0176	1.72	up	chr2:201677547-201677683
ASK05012REF_TFRC_AS001	0.002544	0.0176	1.72	up	chr3:195776191-195776357
ASK05416REF_THBS4_AS001	0.004206	0.0256	1.72	up	chr5:79378208-79378353
ASK07102REF_LARGE_AS001	0.004344	0.0262	1.72	up	chr22:33669257-33669740
TI16954REF_SLC12A4_AS003	0.005289	0.0302	1.72	up	chr16:67995922-67996084
ASK03079REF	0.006551	0.0342	1.72	up	ENST00000469305
TI19214REF_PROC_AS002	0.015969	0.0611	1.72	up	chr2:128184566-128184666
TI16218REF_SEC31A_AS010	0.021053	0.074	1.72	up	chr4:83818286-83818416
TI16933REF_PCID2_AS002	0.046832	0.131	1.72	up	chr13:113837482-113837640
TI21821REF_PFDN5_AS001	0.000424	0.00511	1.71	up	chr12:53689801-53689967
TI16902REF_PPRC1_AS001	0.000702	0.00718	1.71	up	chr10:103907407-103907504
NC00572FR	0.00491	0.0286	1.71	up	FR387370
TI17391REF_SYT12_AS004	0.008468	0.0401	1.71	up	chr11:66798201-66798332
TI17816REF_PHKG2_AS003	0.020947	0.0738	1.71	up	chr16:30763742-30763900
TI16706REF_PCMTD2_AS005	0.024743	0.0825	1.71	up	chr20:62888856-62889034
ASK04743REF_THADA_AS004	0.001002	0.00928	1.7	up	chr2:43451148-43451414
TI19772REF_DUSP5_AS001	0.001303	0.0113	1.7	up	chr10:112266485-112266582
TI18461REF_NIF3L1_AS003	0.006645	0.0345	1.7	up	chr2:201755321-201755447
TI16379REF_SRRM2_AS011	0.006758	0.035	1.7	up	chr16:2804062-2804178
ASK03908REF	0.00927	0.0427	1.7	up	ENST00000491456
TI21965REF_FRY_AS001	0.01729	0.0641	1.7	up	chr13:32743214-32743319
TI19772REF_DUSP5_AS002	0.001248	0.011	1.69	up	chr10:112265372-112265500
TI15207REF	0.002359	0.0167	1.69	up	ENST00000505745
NC00388FR	0.010489	0.0464	1.69	up	FR268176
TI16357REF_BCAN_AS010	0.000781	0.00775	1.68	up	chr1:156613165-156613355
NC00277FR	0.00118	0.0105	1.68	up	FR191603
ASK05283REF_ERCC6_AS001	0.001273	0.0112	1.68	up	chr10:50723366-50723653
ASK01019NTNR	0.001703	0.0138	1.68	up	NR_024516
TI16389REF_SPTBN1_AS008	0.002729	0.0187	1.68	up	chr2:54891140-54891250
TI16531REF_ANXA5_AS011	0.000927	0.00887	1.67	up	chr4:122602048-122602181
ASK05718REF_LUM_AS001	0.0022	0.016	1.67	up	chr12:91497423-91497789
TI16560REF_OPCML_AS003	0.005493	0.0308	1.67	up	chr11:133018475-133018589
TI19201REF_C19orf48_AS003	0.006452	0.0339	1.67	up	chr19:51307191-51307301
TI16099REF_KAZN_AS008	0.008196	0.0393	1.67	up	chr1:15393995-15394202
TI17076REF_SCML1_AS007	0.008796	0.0412	1.67	up	chrX:17761438-17761572
TI18691REF_CDK16_AS002	0.001351	0.0117	1.66	up	chrX:47080062-47080164
TI16445REF_THADA_AS006	0.001862	0.0145	1.66	up	chr2:43450178-43450311
TI19543REF_ODF2_AS002	0.003684	0.0232	1.66	up	chr9:131257827-131258088
TI16146REF_FHOD3_AS018	0.004826	0.0283	1.66	up	chr18:34355733-34355834
TI16088REF_OBSCN_AS017	0.005847	0.0318	1.66	up	chr1:228499414-228499523

TI19225REF_CDCA7_AS002	0.023401	0.0793	1.66	up	chr2:174228776-174229095
TI21336REF_LRRC40_AS001	0.046999	0.131	1.66	up	chr1:70613399-70613502
TI23048REF_UBE2C_AS001	0.002506	0.0175	1.65	up	chr20:44442192-44442303
TI16881REF_RNPEP_AS002	0.003284	0.0212	1.65	up	chr1:201962307-201962406
TI16031REF_MAD1L1_AS061	0.004006	0.0246	1.65	up	chr7:2099997-2100116
TI18776REF_MCL1_AS001	0.004495	0.0267	1.65	up	chr1:150550280-150550713
TI16784REF_PDE8A_AS008	0.00655	0.0342	1.65	up	chr15:85637579-85637715
ASK05232REF_SYNGR2_AS001	0.00984	0.0445	1.65	up	chr17:76168131-76168731
TI20920REF_FAM46A_AS001	0.010257	0.0457	1.65	up	chr6:82461001-82461138
TI16343REF_FBLN1_AS012	0.01702	0.0636	1.65	up	chr22:45975588-45975691
TI16464REF_PILRB_AS006	0.002194	0.016	1.64	up	chr7:99947681-99947819
TI22184REF_SNAPC5_AS001	0.002303	0.0164	1.64	up	chr15:66786278-66786689
TI16711REF_SUN2_AS008	0.005486	0.0308	1.64	up	chr22:39136616-39136766
TI16488REF_PHLDB1_AS001	0.007052	0.0358	1.64	up	chr11:118513350-118513467
TI16099REF_KAZN_AS015	0.008133	0.0392	1.64	up	chr1:15392693-15392802
TI17076REF_SCML1_AS004	0.010162	0.0456	1.64	up	chrX:17763859-17763982
NC00526FR	0.010501	0.0464	1.64	up	FR355542
TI16231REF_GPR133_AS005	0.019439	0.0703	1.64	up	chr12:131616029-131616134
TI18083REF_DFNB31_AS001	0.027484	0.089	1.64	up	chr9:117222930-117223071
TI17949REF_SUCLG1_AS002	0.02757	0.0891	1.64	up	chr2:84652024-84652156
TI16976REF_NDUFV2_AS002	0.03592	0.108	1.64	up	chr18:9116134-9116326
TI21657REF_ACAD8_AS001	0.043639	0.124	1.64	up	chr11:134130506-134130616
TI16307REF_SNX5_AS015	0.001786	0.0142	1.63	up	chr20:17925559-17925754
TI16541REF_PRDX6_AS001	0.002038	0.0153	1.63	up	chr1:173452741-173452938
TI17277REF_PVRL3_AS007	0.005329	0.0303	1.63	up	chr3:110918257-110918403
TI17577REF_RPL3_AS003	0.006587	0.0344	1.63	up	chr22:39714078-39714234
TI17760REF_TUBA1C_AS003	0.0272	0.0884	1.63	up	chr12:49665440-49665554
TI16426REF_AKT1_AS006	0.037598	0.111	1.63	up	chr14:105238226-105238393
ASK04417REF	0.001804	0.0143	1.62	up	ENST00000426590
ASK02378REF	0.001986	0.0151	1.62	up	ENST00000555759
TI16091REF_GAK_AS014	0.002478	0.0174	1.62	up	chr4:904051-904192
TI21044REF_TOX_AS002	0.006294	0.0334	1.62	up	chr8:59837546-59837656
TI18751REF_C1orf100_AS002	0.007927	0.0386	1.62	up	chr1:244537464-244537561
TI17393REF_CCDC84_AS003	0.008026	0.0389	1.62	up	chr11:118881056-118881208
TI17362REF_CSF1_AS005	0.015983	0.0611	1.62	up	chr1:110472374-110472620
TI16053REF_SEPT9_AS022	0.02328	0.0791	1.62	up	chr17:75490729-75490881
TI18721REF_B4GALT2_AS003	0.036105	0.108	1.62	up	chr1:44446046-44446170
TI17633REF_MAFK_AS001	0.036514	0.109	1.62	up	chr7:1571943-1572056
TI22781REF_TTL_AS001	0.002386	0.0168	1.61	up	chr2:113280432-113280685
ASK05360REF_ABCC1_AS001	0.00335	0.0216	1.61	up	chr16:16200336-16200840
ASK03267REF	0.003936	0.0243	1.61	up	ENST00000527688
TI18224REF_TAF1D_AS003	0.004495	0.0267	1.61	up	chr11:93468854-93468985
TI16036REF_EIF2B5_AS033	0.008325	0.0396	1.61	up	chr3:184102483-184102601
TI17377REF_MAP3K8_AS001	0.015979	0.0611	1.61	up	chr10:30724167-30724285
ASK07151REF_PRDM1_AS001	0.003286	0.0212	1.6	up	chr6:106556513-106556950
TI18485REF_C2orf89_AS004	0.004355	0.0262	1.6	up	chr2:85097571-85097652
TI17530REF_STEAP3_AS003	0.004842	0.0283	1.6	up	chr2:119987569-119987700
TI18241REF_TXNRD1_AS001	0.005611	0.031	1.6	up	chr12:104683383-104683472
TI21532REF_ATP5C1_AS001	0.006448	0.0339	1.6	up	chr10:7841543-7841669
TI16899REF_KCND3_AS008	0.01071	0.0468	1.6	up	chr1:112375155-112375249
TI23838REF_OPN1SW_AS001	0.010887	0.0473	1.6	up	chr7:128412883-128413009
TI18929REF_CAPRIN2_AS003	0.011575	0.0492	1.6	up	chr12:30868641-30868899
TI16624REF_LGR6_AS003	0.03227	0.0995	1.6	up	chr1:202188378-202188515
TI18341REF_RABEP1_AS004	0.00327	0.0212	1.59	up	chr17:5281956-5282062
TI21297REF_S100A7A_AS001	0.005006	0.0289	1.59	up	chr1:153390171-153390305

TI17532REF_EPAS1_AS006	0.007587	0.0374	1.59	up	chr2:46598694-46598805
TI16640REF_EIF4G2_AS009	0.008848	0.0414	1.59	up	chr11:10828198-10828345
TI17481REF_ACBD4_AS003	0.009672	0.0439	1.59	up	chr17:43217184-43217339
TI17770REF_ATP5B_AS004	0.011602	0.0492	1.59	up	chr12:57037437-57037536
TI17696REF_SLC30A1_AS002	0.01475	0.0585	1.59	up	chr1:211746177-211746275
TI16360REF_NFIA_AS009	0.014907	0.0589	1.59	up	chr1:61588078-61588193
TI16474REF_SRGAP2_AS002	0.021419	0.0747	1.59	up	chr1:206630781-206630925
TI23378REF_CRYGS_AS001	0.006495	0.0341	1.58	up	chr3:186258810-186258930
NC00249FR	0.007374	0.0369	1.58	up	FR173272
TI19863REF_NCR3LG1_AS002	0.007633	0.0375	1.58	up	chr11:17388640-17388874
TI12885REF	0.007871	0.0386	1.58	up	ENST00000464888
TI18961REF_SCFD1_AS003	0.008112	0.0392	1.58	up	chr14:31184212-31184318
TI16064REF_INPP5A_AS028	0.018049	0.0665	1.58	up	chr10:134352412-134352558
TI18650REF_CPSF1_AS001	0.025251	0.0837	1.58	up	chr8:145621030-145621177
TI19525REF_RPL8_AS002	0.002817	0.0191	1.57	up	chr8:146015940-146016045
TI18209REF_RIC8A_AS002	0.002909	0.0195	1.57	up	chr11:213121-213272
TI17045REF_CTNNA1_AS003	0.004172	0.0255	1.57	up	chr5:138266811-138267029
TI17841REF_RPAIN_AS003	0.004619	0.0273	1.57	up	chr17:5331022-5331309
TI18257REF_NAP1L1_AS003	0.007551	0.0374	1.57	up	chr12:76443192-76443293
TI17215REF_RPS9_AS006	0.007895	0.0386	1.57	up	chr19:54706510-54706695
TI16267REF_PABPC1L_AS009	0.011033	0.0478	1.57	up	chr20:43567798-43567934
TI20915REF_BCLAF1_AS001	0.017936	0.0661	1.57	up	chr6:136592513-136592594
TI18766REF_USP48_AS001	0.026161	0.0858	1.57	up	chr1:22052645-22052726
TI16933REF_PCID2_AS005	0.040256	0.118	1.57	up	chr13:113837258-113837428
ASK06571REF_B2M_AS001	0.002754	0.0188	1.56	up	chr15:45009873-45010302
TI19004REF_TLN2_AS003	0.00447	0.0267	1.56	up	chr15:63136601-63136724
NC00382FR	0.006843	0.0353	1.56	up	FR265193
ASK03447REF	0.007485	0.0372	1.56	up	ENST00000316848
TI17271REF_RBM5_AS003	0.008749	0.0411	1.56	up	chr3:50132919-50133069
TI17633REF_MAFK_AS005	0.040785	0.119	1.56	up	chr7:1576286-1576437
TI17224REF_EEF2_AS001	0.041855	0.121	1.56	up	chr19:3981626-3981728
TI17528REF_NOP58_AS001	0.045815	0.129	1.56	up	chr2:203149467-203149590
TI16636REF_DDB2_AS003	0.003193	0.0209	1.55	up	chr11:47240191-47240283
TI17346REF_GNPAT_AS003	0.005999	0.0323	1.55	up	chr1:231405718-231405806
TI19357REF_PLCL2_AS002	0.009	0.0419	1.55	up	chr3:16927844-16927947
NC00516FR	0.010724	0.0468	1.55	up	FR352059
TI16307REF_SNX5_AS001	0.0124	0.0513	1.55	up	chr20:17940249-17940383
TI19751REF_FHL3_AS001	0.014584	0.058	1.55	up	chr1:38470586-38470739
TI18072REF_ARPC5L_AS004	0.01642	0.062	1.55	up	chr9:127624251-127624413
TI16929REF_SBNO1_AS008	0.02379	0.0803	1.55	up	chr12:123774303-123774402
TI22811REF_HAT1_AS001	0.024178	0.0812	1.55	up	chr2:172779403-172779517
TI18092REF_PPP1R3F_AS004	0.025217	0.0837	1.55	up	chrX:49131571-49131677
TI18494REF_RBCK1_AS001	0.041595	0.121	1.55	up	chr20:400943-401122
TI21240REF_MIA3_AS001	0.005005	0.0289	1.54	up	chr1:222835828-222835989
TI17411REF_HNRNPA1_AS002	0.005153	0.0296	1.54	up	chr12:54674751-54674841
TI17507REF_SNRNP70_AS005	0.005444	0.0308	1.54	up	chr19:49609062-49609332
TI16343REF_FBLN1_AS001	0.006131	0.0328	1.54	up	chr22:45958680-45958790
TI16483REF_DAB1_AS012	0.010323	0.046	1.54	up	chr1:57735713-57735856
TI20744REF_C3orf56_AS002	0.011578	0.0492	1.54	up	chr3:126912261-126912424
TI16687REF_TGIF1_AS005	0.016403	0.062	1.54	up	chr18:3452538-3452624
TI16109REF_MICAL3_AS003	0.018325	0.0673	1.54	up	chr22:18371849-18371969
TI16531REF_ANXA5_AS002	0.038924	0.115	1.54	up	chr4:122600163-122600257
TI16578REF_DDX5_AS005	0.04867	0.134	1.54	up	chr17:62501066-62501438
TI13456REF	0.005447	0.0308	1.53	up	ENST00000549154
TI16636REF_DDB2_AS001	0.006899	0.0354	1.53	up	chr11:47256524-47256699

TI01454NTNR	0.007199	0.0363	1.53	up	NR_040718
TI19795REF_EIF3A_AS001	0.007922	0.0386	1.53	up	chr10:120810227-120810403
TI18603REF_PCDH1_AS001	0.012473	0.0513	1.53	up	chr5:141245672-141245772
TI16541REF_PRDX6_AS002	0.01404	0.0564	1.53	up	chr1:173449359-173449479
TI16968REF_VPS53_AS007	0.015102	0.0592	1.53	up	chr17:558104-558199
TI16933REF_PCID2_AS006	0.017661	0.0652	1.53	up	chr13:113838202-113838326
TI17675REF_ROR1_AS005	0.019851	0.0714	1.53	up	chr1:64503964-64504065
TI16035REF_MSI2_AS009	0.047489	0.132	1.53	up	chr17:55750490-55750620
TI08300REF	0.005472	0.0308	1.52	up	ENST00000493650
TI20515REF_AGFG1_AS002	0.006985	0.0357	1.52	up	chr2:228393463-228393551
TI17615REF_BRD9_AS006	0.008754	0.0411	1.52	up	chr5:865860-865975
TI20702REF_DNAJB11_AS001	0.009079	0.0421	1.52	up	chr3:186297543-186297655
TI20130REF_PRC1_AS002	0.011485	0.0491	1.52	up	chr15:91512537-91512664
TI17262REF_hsa-mir-4763_AS00	0.013009	0.053	1.52	up	chr22:46503634-46503740
TI16779REF_RILPL1_AS009	0.015795	0.061	1.52	up	chr12:124013345-124013502
TI16933REF_PCID2_AS008	0.016857	0.0631	1.52	up	chr13:113836159-113836719
TI18242REF_M6PR_AS003	0.023878	0.0805	1.52	up	chr12:9094569-9094687
TI17913REF_ATP6V1B1_AS002	0.029237	0.0925	1.52	up	chr2:71187604-71187723
TI17231REF_SLC20A1_AS006	0.044578	0.126	1.52	up	chr2:113418613-113418709
TI19595REF_THOC2_AS002	0.048209	0.133	1.52	up	chrX:122754052-122754232
ASK06228REF_SH3D19_AS001	0.008991	0.0419	1.51	up	chr4:152041843-152041962
TI16652REF_PPP2R5C_AS005	0.010627	0.0467	1.51	up	chr14:102325129-102325222
TI16530REF_HNRNPD_AS010	0.013011	0.053	1.51	up	chr4:83280835-83281010
TI17005REF_SS18L1_AS002	0.015399	0.0597	1.51	up	chr20:60731117-60731259
TI19031REF_TNRC6A_AS002	0.016239	0.0616	1.51	up	chr16:24831760-24831860
TI17622REF_SHROOM1_AS004	0.024324	0.0815	1.51	up	chr5:132165186-132165334
TI16922REF_CCDC82_AS006	0.030706	0.0963	1.51	up	chr11:96118316-96118496
TI17430REF_EVL_AS002	0.033886	0.103	1.51	up	chr14:100563997-100564120
TI17534REF_PTCD3_AS005	0.041844	0.121	1.51	up	chr2:86362491-86362639
TI16334REF_RP11-159D12.5_AS	0.048556	0.134	1.51	up	chr17:56076958-56077097
TI19381REF_ATP13A3_AS002	0.004234	0.0257	1.5	up	chr3:194131713-194131857
ASK02951REF	0.009486	0.0435	1.5	up	ENST00000481346
TI16042REF_ANKRD11_AS028	0.012385	0.0513	1.5	up	chr16:89547367-89547528
TI17277REF_PVRL3_AS004	0.01265	0.0517	1.5	up	chr3:110919634-110919781
TI20787REF_MAN2B2_AS001	0.017151	0.064	1.5	up	chr4:6592244-6592389
TI17686REF_PRDX1_AS002	0.027476	0.089	1.5	up	chr1:45982738-45982879
TI19412REF_RPL9_AS003	0.0336	0.103	1.5	up	chr4:39457430-39457564
TI01382NTNR	0.004553	0.027	1.49	up	NR_026878
ASK06052REF_PCDHGA2_AS00	0.005499	0.0308	1.49	up	chr5:140891676-140892379
TI18271REF_KHNYN_AS001	0.031027	0.0971	1.49	up	chr14:24904097-24904246
TI18321REF_IST1_AS002	0.034668	0.105	1.49	up	chr16:71958508-71958665
TI16959REF_AKAP1_AS002	0.043447	0.124	1.49	up	chr17:55184566-55184733
ASK07015REF_BARD1_AS001	0.045321	0.128	1.49	up	chr2:215657141-215657683
TI17277REF_PVRL3_AS006	0.006166	0.0329	1.48	up	chr3:110919476-110919578
TI18995REF_CAPN3_AS002	0.010368	0.0461	1.48	up	chr15:42645380-42645462
ASK02612REF	0.011599	0.0492	1.48	up	ENST00000482327
TI10663REF	0.018423	0.0675	1.48	up	ENST00000483622
TI18058REF_MYC_AS005	0.019174	0.0695	1.48	up	chr8:128748939-128749049
TI17625REF_GNB2L1_AS002	0.019922	0.0716	1.48	up	chr5:180665886-180666044
NC00444FR	0.020434	0.0729	1.48	up	FR309907
TI22178REF_KIAA0101_AS001	0.02085	0.0736	1.48	up	chr15:64665922-64666030
TI17164REF_MAN2C1_AS002	0.02101	0.074	1.48	up	chr15:75651835-75651940
TI19928REF_COQ10A_AS002	0.021111	0.074	1.48	up	chr12:56661835-56661926
TI16448REF_GALNT14_AS010	0.02412	0.0811	1.48	up	chr2:31306721-31306806
TI16908REF_MGEA5_AS001	0.029826	0.0941	1.48	up	chr10:103553933-103554045

TI17750REF_CKAP5_AS001	0.031534	0.0981	1.48	up	chr11:46776279-46776377
TI16307REF_SNX5_AS013	0.038459	0.114	1.48	up	chr20:17940095-17940207
TI16864REF_GATS_AS004	0.047218	0.131	1.48	up	chr7:99812179-99812259
TI17940REF_NCL_AS002	0.049662	0.135	1.48	up	chr2:232328141-232328243
TI19745REF_SLC26A9_AS001	0.007304	0.0367	1.47	up	chr1:205907248-205907355
TI17864REF_SNF8_AS004	0.011144	0.048	1.47	up	chr17:47009097-47009195
TI16540REF_CD46_AS010	0.015816	0.061	1.47	up	chr1:207942634-207943035
TI20853REF_TNFAIP8_AS001	0.017337	0.0641	1.47	up	chr5:118686515-118686622
TI21405REF_SUSD4_AS001	0.022466	0.0771	1.47	up	chr1:223467796-223467901
TI20605REF_UQCC_AS002	0.022535	0.0772	1.47	up	chr20:33893337-33893458
ASK05967REF_KLF13_AS001	0.022645	0.0773	1.47	up	chr15:31669408-31669947
TI16673REF_TOM1L1_AS004	0.027126	0.0884	1.47	up	chr17:53033030-53033185
TI17860REF_GGA3_AS001	0.045957	0.129	1.47	up	chr17:73238022-73238140
TI19684REF_WARS2_AS002	0.008574	0.0404	1.46	up	chr1:119627736-119627867
TI24189REF_ZDHHC9_AS001	0.011268	0.0484	1.46	up	chrX:128974720-128974873
TI16758REF_RCC2_AS009	0.015407	0.0597	1.46	up	chr1:17736332-17736449
TI19255REF_ODC1_AS002	0.016571	0.0624	1.46	up	chr2:10586825-10586917
TI18613REF_SOD2_AS002	0.017208	0.064	1.46	up	chr6:160102047-160102150
TI16327REF_KIF22_AS010	0.019358	0.0701	1.46	up	chr16:29813784-29813978
TI16309REF_TRA2B_AS002	0.023202	0.0789	1.46	up	chr3:185650945-185651068
TI17940REF_NCL_AS004	0.049055	0.134	1.46	up	chr2:232320544-232320700
TI16514REF_PKP4_AS004	0.02175	0.0756	1.45	up	chr2:159518180-159518283
TI16049REF_DSCAML1_AS033	0.024419	0.0816	1.45	up	chr11:117381508-117381593
TI12111REF	0.032235	0.0995	1.45	up	ENST00000471023
TI19698REF_NGF_AS001	0.034427	0.104	1.45	up	chr1:115847395-115847486
TI13101REF	0.034477	0.105	1.45	up	ENST00000475468
TI16049REF_DSCAML1_AS006	0.040507	0.118	1.45	up	chr11:117463912-117464032
TI17610REF_SLC7A11_AS003	0.043433	0.124	1.45	up	chr4:139126718-139126858
TI16307REF_SNX5_AS016	0.008548	0.0404	1.44	up	chr20:17942662-17942811
TI16640REF_EIF4G2_AS010	0.018472	0.0675	1.44	up	chr11:10825209-10825310
ASK06729REF_HEATR5A_AS00	0.026125	0.0858	1.44	up	chr14:31771500-31771739
TI18685REF_LCN6_AS004	0.033266	0.102	1.44	up	chr9:139640932-139641034
TI17214REF_CALM3_AS002	0.033415	0.102	1.44	up	chr19:47109131-47109393
TI16309REF_TRA2B_AS006	0.038031	0.113	1.44	up	chr3:185648929-185649174
ASK04136REF	0.04487	0.127	1.44	up	ENST00000489987
TI17947REF_TMEM18_AS003	0.010069	0.0453	1.43	up	chr2:673144-673325
TI09786REF	0.015037	0.0591	1.43	up	ENST00000584906
ASK05332REF_SERPINF1_AS00	0.028133	0.0899	1.43	up	chr17:1680741-1680865
TI17986REF_ITPR1_AS004	0.029961	0.0943	1.43	up	chr3:4815263-4815382
ASK06028REF_FOXQ1_AS001	0.0389	0.115	1.43	up	chr6:1309640-1313530
TI17146REF_GRTP1_AS007	0.040542	0.118	1.43	up	chr13:113987624-113987747
TI20994REF_KLRG2_AS002	0.045241	0.128	1.43	up	chr7:139139101-139139235
TI16937REF_MEF2A_AS004	0.024397	0.0816	1.42	up	chr15:100254256-100254419
TI20676REF_CENPM_AS002	0.027668	0.0892	1.42	up	chr22:42338060-42338165
TI16053REF_SEPT9_AS036	0.028042	0.0898	1.42	up	chr17:75402329-75402445
TI16488REF_PHLDB1_AS008	0.040289	0.118	1.42	up	chr11:118484430-118484523
TI23740REF_TRIM26_AS001	0.048922	0.134	1.42	up	chr6:30163490-30163587
TI21880REF_LMO3_AS001	0.031774	0.0986	1.41	up	chr12:16729230-16729320
ASK02165REF	0.041961	0.121	1.41	up	ENST00000485153
TI16482REF_IFFO2_AS005	0.042933	0.123	1.41	up	chr1:19274295-19274424
TI19101REF_DHX33_AS003	0.020172	0.0722	1.4	up	chr17:5345046-5345181
TI18044REF_CCM2_AS003	0.02234	0.0768	1.4	up	chr7:45112734-45112840
TI17836REF_RP11-1035H13.3_A:	0.031834	0.0986	1.4	up	chr16:18807273-18807369
TI16324REF_SF1_AS006	0.041817	0.121	1.4	up	chr11:64540300-64540635
TI16047REF_SORCS2_AS036	0.044516	0.126	1.4	up	chr4:7346482-7346594

ASK05075REF_LRRC8A_AS001	0.015982	0.0611	1.39	up	chr9:131669864-131670208
ASK04490REF	0.032754	0.101	1.39	up	ENST00000467706
TI16657REF_RORA_AS003	0.043757	0.124	1.39	up	chr15:61477933-61478060
TI16247REF_HNRNPA2B1_AS01	0.044907	0.127	1.39	up	chr7:26233088-26233189
TI22342REF_PSMB3_AS001	0.028375	0.0904	1.38	up	chr17:36916933-36917032
TI16459REF_ZXDC_AS006	0.039918	0.117	1.38	up	chr3:126161148-126161246
ASK05144REF_NEURL1B_AS00	0.022294	0.0767	1.37	up	chr5:172117470-172124726
TI19104REF_P2RX1_AS003	0.028774	0.0913	1.37	up	chr17:3808739-3808837
TI07767REF	0.029965	0.0943	1.37	up	ENST00000496055
TI21742REF_HYOU1_AS001	0.043503	0.124	1.37	up	chr11:118927025-118927186
TI17276REF_ZBTB38_AS003	0.044005	0.125	1.37	up	chr3:141052132-141052257
ASK04955REF_SGOL1_AS001	0.027066	0.0883	1.36	up	chr3:20215753-20227736
TI17137REF_PTMS_AS004	0.030751	0.0963	1.36	up	chr12:6876917-6877096
TI21877REF_C12orf75_AS001	0.02574	0.085	1.35	up	chr12:105764406-105764690
TI20878REF_LMAN2_AS002	0.028761	0.0913	1.35	up	chr5:176759568-176759658
TI16155REF_CTTN_AS005	0.042982	0.123	1.35	up	chr11:70280415-70280551
TI15313REF	0.046308	0.13	1.35	up	ENST00000513162
TI16552REF_UBE2J2_AS011	0.044599	0.126	1.34	up	chr1:1197883-1198018
TI16040REF_VAV2_AS006	0.048589	0.134	1.34	up	chr9:136803974-136804105
TI16537REF_DOCK5_AS007	0.048791	0.134	1.32	up	chr8:25272549-25272693
TI19042REF_C16orf52_AS001	0.049806	0.136	0.75	down	chr16:22089346-22089441
TI17015REF_RUNX1_AS002	0.044906	0.127	0.74	down	chr21:36237869-36237968
TI17708REF_KIAA1217_AS003	0.022062	0.0761	0.73	down	chr10:24833622-24833738
TI18046REF_ABCA13_AS005	0.027696	0.0892	0.73	down	chr7:48350394-48350485
TI18885REF_PSMA1_AS003	0.033288	0.102	0.73	down	chr11:14535434-14535704
TI16135REF_TBCD_AS021	0.035452	0.107	0.73	down	chr17:80764510-80764664
TI17194REF_HDAC5_AS005	0.035887	0.108	0.73	down	chr17:42187028-42187146
TI16057REF_ZMIZ1_AS007	0.036277	0.108	0.73	down	chr10:81029305-81029467
TI19435REF_ADAMTS6_AS001	0.040911	0.119	0.73	down	chr5:64509884-64510033
TI19056REF_CDR2_AS003	0.04699	0.131	0.73	down	chr16:22432411-22432520
TI17299REF_SREK1_AS006	0.014289	0.0571	0.72	down	chr5:65461128-65461245
TI16877REF_RASAL2_AS003	0.015016	0.0591	0.72	down	chr1:178445947-178446129
TI16099REF_KAZN_AS026	0.026806	0.0878	0.72	down	chr1:15394827-15394937
TI20716REF_MED12L_AS001	0.028154	0.0899	0.72	down	chr3:150902474-150902568
TI21404REF_INTS7_AS001	0.030292	0.0951	0.72	down	chr1:212140900-212140988
TI16590REF_NINL_AS008	0.031546	0.0981	0.72	down	chr20:25524747-25524883
TI16985REF_ZNF638_AS003	0.034728	0.105	0.72	down	chr2:71574189-71574308
TI16048REF_CUX1_AS034	0.037332	0.111	0.72	down	chr7:101470078-101470177
TI02008NTNR	0.047628	0.132	0.72	down	NR_024628
TI02009NTNR	0.047628	0.132	0.72	down	NR_024629
TI02010NTNR	0.047628	0.132	0.72	down	NR_027708
TI02011NTNR	0.047628	0.132	0.72	down	NR_027709
TI23678REF_SERPINB1_AS001	0.048878	0.134	0.72	down	chr6:2839793-2839907
TI16169REF_ELL2_AS012	0.01353	0.0547	0.71	down	chr5:95296832-95296987
TI16666REF_BCAR1_AS003	0.020681	0.0734	0.71	down	chr16:75273016-75273127
TI17183REF_GOSR2_AS006	0.023788	0.0803	0.71	down	chr17:45047189-45047305
TI19062REF_DEXI_AS003	0.026902	0.0879	0.71	down	chr16:11034659-11034799
TI18155REF_RAB3B_AS004	0.034976	0.106	0.71	down	chr1:52456366-52456477
TI01985NTNR	0.036759	0.11	0.71	down	NR_023359
TI17338REF_PHKA2_AS005	0.042257	0.121	0.71	down	chrX:18916472-18916612
TI16638REF_MYO7A_AS004	0.011568	0.0492	0.7	down	chr11:76900635-76900752
TI20742REF_KBTBD12_AS001	0.011958	0.0502	0.7	down	chr3:127647437-127647549
TI21134REF_NLGN4X_AS001	0.014247	0.0571	0.7	down	chrX:6145192-6145286
TI16043REF_CTIF_AS045	0.021941	0.076	0.7	down	chr18:46383712-46383818
TI16062REF_ADARB2_AS025	0.028601	0.091	0.7	down	chr10:1334052-1334188

TI17173REF_PPL_AS005	0.03364	0.103	0.7	down	chr16:4979197-4979321
TI02002NTNR	0.040192	0.118	0.7	down	NR_038366
TI02003NTNR	0.040192	0.118	0.7	down	NR_038367
TI19238REF_KCNK3_AS002	0.046905	0.131	0.7	down	chr2:26932771-26932868
ASK01003NTNR	0.049873	0.136	0.7	down	NR_037705
TI17331REF_DENND1A_AS007	0.007782	0.0382	0.69	down	chr9:126218550-126218661
TI22400REF_SHISA6_AS001	0.009575	0.0437	0.69	down	chr17:11146400-11146513
TI16635REF_FRMD4A_AS002	0.011336	0.0486	0.69	down	chr10:13721864-13721994
TI02082NTNR	0.012161	0.0507	0.69	down	XR_109464
TI17427REF_WDR20_AS002	0.014134	0.0567	0.69	down	chr14:102684592-102684715
TI16196REF_SPG7_AS019	0.01593	0.0611	0.69	down	chr16:89588742-89589093
TI17814REF_PMM2_AS001	0.019053	0.0692	0.69	down	chr16:8926616-8926719
TI16112REF_BCAR3_AS003	0.020462	0.0729	0.69	down	chr1:94119491-94119643
TI16481REF_SSU72_AS007	0.026038	0.0856	0.69	down	chr1:1496780-1496939
TI23721REF_FAXC_AS001	0.027146	0.0884	0.69	down	chr6:99763928-99764008
ASK06292REF_NDUFV3_AS001	0.028321	0.0903	0.69	down	chr21:44322448-44322831
TI17570REF_ZDHHC8_AS003	0.029459	0.093	0.69	down	chr22:20129867-20129976
TI16637REF_FADS2_AS004	0.032847	0.101	0.69	down	chr11:61606234-61606376
TI17229REF_GLS_AS005	0.035204	0.106	0.69	down	chr2:191790756-191790890
TI16357REF_BCAN_AS009	0.036982	0.11	0.69	down	chr1:156615684-156615835
TI16121REF_NPAS2_AS014	0.039251	0.116	0.69	down	chr2:101605479-101605575
TI17011REF_TRPM2_AS003	0.040475	0.118	0.69	down	chr21:45853286-45853383
ASK01037NTNR	0.041159	0.119	0.69	down	NR_038246
TI17473REF_THRA_AS004	0.04283	0.123	0.69	down	chr17:38223441-38223569
TI19398REF_ANK2_AS002	0.006611	0.0344	0.68	down	chr4:113937611-113937715
TI21211REF_FNBP1L_AS001	0.009342	0.043	0.68	down	chr1:93913716-93913833
TI16445REF_THADA_AS004	0.011034	0.0478	0.68	down	chr2:43420190-43420354
TI23224REF_NKTR_AS001	0.012515	0.0513	0.68	down	chr3:42686286-42686417
TI16367REF_TCF7L2_AS007	0.014925	0.0589	0.68	down	chr10:114805286-114805383
ASK05798REF_HNRNPF_AS001	0.018213	0.067	0.68	down	chr10:43881139-43881486
TI16781REF_ACIN1_AS001	0.020066	0.072	0.68	down	chr14:23534231-23534333
TI16357REF_BCAN_AS004	0.025908	0.0853	0.68	down	chr1:156613514-156613833
TI18665REF_SNAPC3_AS001	0.031635	0.0983	0.68	down	chr9:15462331-15462599
ASK05401REF_NAA30_AS001	0.035965	0.108	0.68	down	chr14:57857556-57857939
TI16246REF_GTF2IRD1_AS010	0.040648	0.118	0.68	down	chr7:74012982-74013084
TI19694REF_RAB3GAP2_AS001	0.049603	0.135	0.68	down	chr1:220323209-220323351
TI19584REF_SAT1_AS001	0.008239	0.0395	0.67	down	chrX:23802735-23803405
ASK05690REF_CHSY1_AS001	0.013875	0.0558	0.67	down	chr15:101716152-101716603
TI16772REF_IFITM10_AS005	0.015186	0.0593	0.67	down	chr11:1771549-1771650
ASK04968REF_MEOX2_AS001	0.015206	0.0593	0.67	down	chr7:15718652-15721463
TI16525REF_CCDC50_AS011	0.016208	0.0616	0.67	down	chr3:191112665-191112758
TI16507REF_BRCA1_AS006	0.025799	0.0851	0.67	down	chr17:41295801-41295903
TI16452REF_SHANK3_AS012	0.025924	0.0853	0.67	down	chr22:51162740-51162837
ASK06309REF_BACE2_AS001	0.027759	0.0893	0.67	down	chr21:42537369-42540491
TI17171REF_VAC14_AS002	0.028042	0.0898	0.67	down	chr16:70752548-70752697
TI18328REF_TMEM8A_AS001	0.031289	0.0976	0.67	down	chr16:428585-428738
TI16893REF_ENSA_AS006	0.036324	0.108	0.67	down	chr1:150592545-150592661
TI16087REF_HDLBP_AS015	0.038516	0.114	0.67	down	chr2:242201224-242201318
TI16638REF_MYO7A_AS010	0.04039	0.118	0.67	down	chr11:76901927-76902058
TI22258REF_ACD_AS001	0.009691	0.0439	0.66	down	chr16:67693168-67693261
TI16702REF_SESTD1_AS001	0.010402	0.0461	0.66	down	chr2:179967836-179968070
TI17829REF_DYNC1LI2_AS004	0.011444	0.049	0.66	down	chr16:66764324-66765250
NC00039FR	0.011941	0.0502	0.66	down	FR020479
TI16750REF_DDI2_AS002	0.012045	0.0505	0.66	down	chr1:15991912-15992022
TI16545REF_MAPKAPK2_AS00	0.01214	0.0507	0.66	down	chr1:206877102-206877204

TI16362REF_PABPC4_AS012	0.015044	0.0591	0.66	down	chr1:40034127-40034265
TI18825REF_RBM20_AS003	0.015592	0.0604	0.66	down	chr10:112455174-112455308
TI16216REF_TIA1_AS015	0.016201	0.0616	0.66	down	chr2:70455378-70455469
TI17623REF_TMEM167A_AS006	0.020419	0.0729	0.66	down	chr5:82350697-82350944
ASK00784NTNR	0.022004	0.0761	0.66	down	NR_038289
TI22936REF_LYG1_AS001	0.022478	0.0771	0.66	down	chr2:99919922-99920018
TI16361REF_SLC35F3_AS011	0.02907	0.0921	0.66	down	chr1:234359024-234359107
TI17457REF_ZC3H18_AS001	0.032748	0.101	0.66	down	chr16:88646975-88647116
TI24057REF_MED22_AS001	0.034152	0.104	0.66	down	chr9:136213839-136213986
TI16864REF_GATS_AS005	0.037367	0.111	0.66	down	chr7:99813141-99813261
TI13722REF	0.041805	0.121	0.66	down	ENST00000558649
TI16190REF_LRP5_AS007	0.045887	0.129	0.66	down	chr11:68156396-68156557
TI22696REF_SLC8A2_AS001	0.049262	0.135	0.66	down	chr19:47963116-47963207
TI16703REF_GFPT1_AS006	0.003875	0.024	0.65	down	chr2:69548052-69548145
TI17476REF_MINK1_AS004	0.004676	0.0276	0.65	down	chr17:4754768-4754931
TI16751REF_TARBP1_AS001	0.009081	0.0421	0.65	down	chr1:234543747-234543929
TI18883REF_PITPNM1_AS002	0.009637	0.0439	0.65	down	chr11:67268088-67268191
TI16393REF_PXDN_AS014	0.01244	0.0513	0.65	down	chr2:1666387-1666518
TI20368REF_PRMT1_AS001	0.014552	0.0579	0.65	down	chr19:50188436-50188573
TI16116REF_ANKRD10_AS007	0.014619	0.058	0.65	down	chr13:111547972-111548108
ASK06267REF_CELF2_AS001	0.015211	0.0593	0.65	down	chr10:11375343-11375872
TI18583REF_ZNF827_AS001	0.015871	0.0611	0.65	down	chr4:146681458-146681556
TI18505REF_CDH22_AS002	0.016751	0.0628	0.65	down	chr20:44926556-44926659
TI16038REF_CELF4_AS035	0.017156	0.064	0.65	down	chr18:35125498-35125612
TI16215REF_SPEG_AS008	0.017189	0.064	0.65	down	chr2:220356925-220357033
TI20338REF_RNF152_AS002	0.020581	0.0732	0.65	down	chr18:59538022-59538124
TI18859REF_SIDT2_AS003	0.021362	0.0746	0.65	down	chr11:117062743-117062831
TI17068REF_RC3H2_AS007	0.022152	0.0763	0.65	down	chr9:125607848-125608105
TI17790REF_C14orf166B_AS005	0.027564	0.0891	0.65	down	chr14:77317462-77317580
ASK03582REF	0.031975	0.0988	0.65	down	ENST00000573505
TI16865REF_ST3GAL1_AS002	0.001963	0.015	0.64	down	chr8:134469071-134469269
TI16137REF_LPP_AS011	0.002524	0.0175	0.64	down	chr3:188603087-188603263
TI17175REF_EMP2_AS004	0.004192	0.0256	0.64	down	chr16:10623914-10624024
ASK03493REF	0.00436	0.0262	0.64	down	ENST00000568073
TI16994REF_LIMS1_AS003	0.005683	0.0312	0.64	down	chr2:109301309-109301511
TI16924REF_ETV6_AS006	0.007547	0.0374	0.64	down	chr12:11867154-11867292
TI16046REF_KCNQ1_AS007	0.010412	0.0461	0.64	down	chr11:2752952-2753064
ASK02877REF	0.011859	0.0501	0.64	down	ENST00000486089
TI17355REF_PPFIA4_AS005	0.012475	0.0513	0.64	down	chr1:203020183-203020288
TI16781REF_ACIN1_AS009	0.018628	0.0679	0.64	down	chr14:23534838-23535040
TI16186REF_PRRC2B_AS009	0.0218	0.0756	0.64	down	chr9:134366492-134366777
TI17369REF_TNNI1_AS006	0.024545	0.0819	0.64	down	chr1:201388755-201388867
TI16531REF_ANXA5_AS009	0.031926	0.0988	0.64	down	chr4:122600458-122600613
TI16682REF_P4HB_AS001	0.047624	0.132	0.64	down	chr17:79817676-79817833
TI16153REF_ELK4_AS011	0.048089	0.133	0.64	down	chr1:205577737-205577872
TI12849REF	0.048236	0.133	0.64	down	ENST00000469401
TI20126REF_WDR73_AS002	0.001361	0.0117	0.63	down	chr15:85189076-85189191
TI17736REF_SPON1_AS005	0.001518	0.0127	0.63	down	chr11:14253990-14254085
TI17056REF_SUN1_AS003	0.002627	0.018	0.63	down	chr7:892309-892447
TI10260REF	0.003233	0.0211	0.63	down	ENST00000515753
TI17127REF_PIK3C2A_AS006	0.004419	0.0265	0.63	down	chr11:17110253-17110371
ASK03897REF	0.004973	0.0289	0.63	down	ENST00000483795
TI17567REF_PTTG1IP_AS005	0.005708	0.0313	0.63	down	chr21:46284837-46284991
TI23982REF_DNAJA1_AS001	0.005753	0.0314	0.63	down	chr9:33039313-33039439
TI19505REF_DDHD2_AS003	0.005983	0.0323	0.63	down	chr8:38119161-38119409

TI17086REF_CHD1L_AS007	0.00621	0.0331	0.63	down	chr1:146741932-146742109
TI17070REF_SH3GLB2_AS002	0.007425	0.037	0.63	down	chr9:131775443-131775635
TI18508REF_BCL2L1_AS004	0.008157	0.0392	0.63	down	chr20:30273771-30273900
TI16124REF_ANKRD52_AS011	0.009552	0.0436	0.63	down	chr12:56632840-56632980
TI22849REF_CCDC104_AS001	0.010075	0.0453	0.63	down	chr2:55757626-55757813
TI20704REF_XYLB_AS001	0.014275	0.0571	0.63	down	chr3:38413941-38414117
TI19312REF_PKNOX1_AS002	0.017302	0.0641	0.63	down	chr21:44413316-44413409
TI16178REF_ASPSCR1_AS006	0.020791	0.0735	0.63	down	chr17:79972207-79972306
TI17948REF_XDH_AS003	0.020797	0.0735	0.63	down	chr2:31584315-31584418
TI20062REF_CDH24_AS002	0.021084	0.074	0.63	down	chr14:23520223-23520344
TI17096REF_CDK11A_AS004	0.022725	0.0775	0.63	down	chr1:1646727-1646893
TI16574REF_UNKL_AS011	0.023938	0.0806	0.63	down	chr16:1428264-1428441
TI17670REF_SMYD2_AS001	0.048789	0.134	0.63	down	chr1:214500648-214500814
TI16074REF_GNAS_AS035	0.000699	0.00718	0.62	down	chr20:57469892-57470071
TI17356REF_RNF207_AS004	0.002089	0.0156	0.62	down	chr1:6272600-6272733
TI19481REF_ZNF789_AS001	0.003182	0.0209	0.62	down	chr7:99078146-99078449
TI16349REF_GSK3B_AS001	0.003712	0.0233	0.62	down	chr3:119541316-119541418
TI16750REF_DDI2_AS008	0.003866	0.024	0.62	down	chr1:15994255-15994364
TI18759REF_RP4-758J18.2_AS00	0.005564	0.0309	0.62	down	chr1:1335859-1336248
TI17316REF_KIAA1549_AS006	0.005955	0.0323	0.62	down	chr7:138579126-138579217
TI17539REF_ANKZF1_AS002	0.005971	0.0323	0.62	down	chr2:220097411-220097561
TI16353REF_NHSL1_AS013	0.006863	0.0353	0.62	down	chr6:138744611-138744745
TI16854REF_PHF10_AS002	0.007395	0.0369	0.62	down	chr6:170108789-170109020
TI17948REF_XDH_AS005	0.008316	0.0396	0.62	down	chr2:31584444-31584554
ASK07206REF_LTBP2_AS001	0.008504	0.0402	0.62	down	chr14:75018884-75026726
TI16212REF_ABCC3_AS002	0.009145	0.0423	0.62	down	chr17:48740138-48740481
TI18308REF_EDC4_AS002	0.009526	0.0436	0.62	down	chr16:67909085-67909178
ASK04637REF	0.009898	0.0447	0.62	down	ENST00000489062
TI23211REF_CRYBG3_AS001	0.010701	0.0468	0.62	down	chr3:97654621-97654731
TI16087REF_HDLBP_AS003	0.011796	0.0499	0.62	down	chr2:242173394-242173474
TI16045REF_GRID1_AS020	0.015314	0.0595	0.62	down	chr10:87675548-87675719
TI21477REF_TAF13_AS001	0.016671	0.0626	0.62	down	chr1:109606885-109606989
TI16993REF_ANTXR1_AS005	0.020143	0.0722	0.62	down	chr2:69442063-69442197
TI16214REF_ENOSF1_AS016	0.022613	0.0773	0.62	down	chr18:693167-693297
TI16754REF_NPHP4_AS001	0.02519	0.0837	0.62	down	chr1:6050069-6050211
TI16190REF_LRP5_AS013	0.034635	0.105	0.62	down	chr11:68156843-68157017
TI16778REF_RSRC2_AS005	0.041916	0.121	0.62	down	chr12:123005223-123005428
TI18856REF_YAP1_AS003	0.04213	0.121	0.62	down	chr11:102056065-102056184
TI16224REF_NUCKS1_AS016	0.001459	0.0123	0.61	down	chr1:205684278-205684442
TI17612REF_ZSWIM6_AS001	0.002173	0.0159	0.61	down	chr5:60835429-60835551
ASK02798REF	0.002401	0.0169	0.61	down	ENST00000530448
TI13060REF	0.002938	0.0196	0.61	down	ENST00000473643
TI16059REF_RBM39_AS033	0.003783	0.0236	0.61	down	chr20:34317970-34318143
TI13698REF	0.007345	0.0368	0.61	down	ENST00000556524
TI16556REF_SORL1_AS002	0.008256	0.0395	0.61	down	chr11:121501260-121501814
TI16040REF_VAV2_AS024	0.012153	0.0507	0.61	down	chr9:136669707-136669804
TI21971REF_ZMYM2_AS001	0.016014	0.0611	0.61	down	chr13:20533689-20533785
TI19515REF_EPB49_AS001	0.018885	0.0688	0.61	down	chr8:21913143-21913272
TI01682NTNR	0.019691	0.0709	0.61	down	XR_108725
TI01683NTNR	0.019691	0.0709	0.61	down	XR_110624
TI01684NTNR	0.019691	0.0709	0.61	down	XR_112964
TI18518REF_MCM3AP_AS002	0.021217	0.0742	0.61	down	chr21:47663186-47663292
TI16482REF_IFFO2_AS006	0.022953	0.0782	0.61	down	chr1:19251756-19251837
TI16330REF_RP11-31I10.4_AS00	0.027909	0.0896	0.61	down	chr16:1831236-1831341
TI16034REF_FNDC3B_AS005	0.028108	0.0899	0.61	down	chr3:171768591-171768724

TI16216REF_TIA1_AS001	0.031398	0.0979	0.61	down	chr2:70455081-70455177
TI13945REF	0.033534	0.103	0.61	down	ENST00000561853
TI16038REF_CELF4_AS015	0.033696	0.103	0.61	down	chr18:34943775-34943895
TI17205REF_DSG2_AS005	0.041718	0.121	0.61	down	chr18:29128405-29128668
TI18662REF_UGCG_AS004	0.000966	0.00911	0.6	down	chr9:114696664-114696830
TI17921REF_SSFA2_AS001	0.00173	0.0139	0.6	down	chr2:182782269-182782464
ASK05115REF_CDCP1_AS001	0.001761	0.0141	0.6	down	chr3:45123770-45124005
ASK06289REF_CDON_AS001	0.00186	0.0145	0.6	down	chr11:125827797-125828423
TI19498REF_TMED4_AS003	0.002581	0.0177	0.6	down	chr7:44617632-44617838
TI16073REF_NFE2L2_AS013	0.003051	0.0202	0.6	down	chr2:178099036-178099160
TI16621REF_GABRE_AS003	0.003425	0.0218	0.6	down	chrX:151124502-151125414
TI16769REF_RPL27A_AS003	0.003725	0.0233	0.6	down	chr11:8709643-8709788
TI18783REF_ENAH_AS002	0.0064	0.0338	0.6	down	chr1:225832698-225832788
ASK04747REF_COPS3_AS001	0.007352	0.0368	0.6	down	chr17:17146620-17150455
ASK06756REF_LRBA_AS001	0.007618	0.0375	0.6	down	chr4:151500778-151502419
ASK05891REF_USP25_AS001	0.007995	0.0388	0.6	down	chr21:17250920-17251178
TI16305REF_NTSR1_AS009	0.01114	0.048	0.6	down	chr20:61373062-61373171
TI16795REF_IFT140_AS003	0.012492	0.0513	0.6	down	chr16:1561623-1561749
TI16096REF_CTBP1_AS019	0.018459	0.0675	0.6	down	chr4:1236619-1237077
TI17291REF_FAM160A1_AS006	0.020617	0.0733	0.6	down	chr4:152584220-152584381
TI17057REF_PON2_AS002	0.020768	0.0735	0.6	down	chr7:95043755-95043863
ASK05773REF_DTWD2_AS001	0.023339	0.0792	0.6	down	chr5:118173046-118173366
TI18049REF_KCTD7_AS003	0.029405	0.093	0.6	down	chr7:66106848-66107023
TI16247REF_HNRNPA2B1_AS01	0.031067	0.0971	0.6	down	chr7:26229812-26229929
TI16112REF_BCAR3_AS005	0.045971	0.129	0.6	down	chr1:94230048-94230135
TI18595REF_RP11-428C6.1_AS0	0.000367	0.00457	0.59	down	chr5:73116394-73116501
TI16308REF_SON_AS014	0.000654	0.00696	0.59	down	chr21:34943878-34943992
ASK06490REF_HEXIM1_AS001	0.001104	0.01	0.59	down	chr17:43227786-43228268
TI16418REF_NAV2_AS005	0.001754	0.0141	0.59	down	chr11:19719523-19719689
TI01691NTNR	0.001939	0.0149	0.59	down	XR_110494
TI16395REF_SIK1_AS012	0.002891	0.0195	0.59	down	chr21:44845152-44845285
TI19449REF_SERINC5_AS002	0.00324	0.0211	0.59	down	chr5:79438327-79438419
TI17533REF_INSIG2_AS003	0.00424	0.0257	0.59	down	chr2:118863265-118863362
TI16134REF_FARP1_AS007	0.004989	0.0289	0.59	down	chr13:99058521-99058804
TI20642REF_DONSON_AS002	0.009706	0.0439	0.59	down	chr21:34954366-34954454
TI19071REF_TUBG2_AS003	0.010235	0.0457	0.59	down	chr17:40815245-40815376
TI17587REF_PXK_AS003	0.011056	0.0478	0.59	down	chr3:58409580-58409690
TI17709REF_REEP3_AS001	0.011911	0.0502	0.59	down	chr10:65383580-65383667
TI19067REF_ERI2_AS002	0.013386	0.0544	0.59	down	chr16:20878524-20878639
TI16354REF_PLXNA4_AS001	0.014352	0.0572	0.59	down	chr7:131850556-131850646
TI19848REF_ANKK1_AS002	0.025315	0.0837	0.59	down	chr11:113266313-113266442
TI16273REF_HMGXB3_AS009	0.037489	0.111	0.59	down	chr5:149432293-149432451
TI17606REF_RBPJ_AS002	0.000251	0.00348	0.58	down	chr4:26434258-26434969
TI16606REF_STC2_AS008	0.000265	0.00366	0.58	down	chr5:172746377-172746475
TI16134REF_FARP1_AS014	0.000479	0.00557	0.58	down	chr13:99058259-99058464
NC00496FR	0.000522	0.00596	0.58	down	FR340981
TI16968REF_VPS53_AS003	0.000621	0.00672	0.58	down	chr17:421611-421768
TI16308REF_SON_AS004	0.001033	0.00951	0.58	down	chr21:34941474-34941588
TI19642REF_PRKAA2_AS002	0.001537	0.0128	0.58	down	chr1:57177030-57177143
TI18610REF_TFB1M_AS001	0.001699	0.0138	0.58	down	chr6:155583434-155583652
TI16137REF_LPP_AS016	0.002175	0.0159	0.58	down	chr3:188598489-188598633
TI18662REF_UGCG_AS001	0.002269	0.0162	0.58	down	chr9:114695598-114695818
TI16994REF_LIMS1_AS002	0.002496	0.0174	0.58	down	chr2:109302131-109302220
TI16989REF_NAB1_AS002	0.002574	0.0177	0.58	down	chr2:191522408-191522524
TI16315REF_XKR6_AS014	0.002934	0.0196	0.58	down	chr8:10876413-10876515

TI16035REF_MSI2_AS014	0.003877	0.024	0.58	down	chr17:55758469-55758566
TI16308REF_SON_AS005	0.004011	0.0246	0.58	down	chr21:34943022-34943194
TI16071REF_GALNT2_AS010	0.00551	0.0308	0.58	down	chr1:230399934-230400109
TI16854REF_PHF10_AS001	0.005558	0.0309	0.58	down	chr6:170109303-170109581
TI17018REF_DESI1_AS001	0.005682	0.0312	0.58	down	chr22:41997480-41997685
TI16196REF_SPG7_AS004	0.012497	0.0513	0.58	down	chr16:89584441-89584926
NC00364FR	0.016599	0.0624	0.58	down	FR251069
TI16160REF_TBC1D16_AS017	0.000319	0.00419	0.57	down	chr17:77910764-77910901
TI17921REF_SSFA2_AS004	0.000362	0.00457	0.57	down	chr2:182781959-182782235
TI17478REF_TANC2_AS001	0.000547	0.0061	0.57	down	chr17:61501768-61501887
TI01632NTNR	0.001283	0.0112	0.57	down	NR_040093
TI12031REF	0.001297	0.0113	0.57	down	ENST00000462712
TI17329REF_C9orf3_AS003	0.001466	0.0123	0.57	down	chr9:97770111-97770227
TI18051REF_LAMB1_AS004	0.001475	0.0124	0.57	down	chr7:107585861-107585977
TI23882REF_SGK3_AS001	0.002802	0.0191	0.57	down	chr8:67589578-67589706
TI16111REF_TMEM181_AS003	0.003401	0.0217	0.57	down	chr6:159055243-159055398
ASK04922REF_JAM2_AS001	0.005234	0.0299	0.57	down	chr21:27086986-27087172
ASK06939REF_CDH2_AS001	0.005656	0.0312	0.57	down	chr18:25530941-25531105
TI16134REF_FARP1_AS010	0.005745	0.0314	0.57	down	chr13:99050012-99050128
TI21992REF_MIPEP_AS001	0.007271	0.0366	0.57	down	chr13:24425079-24425187
TI19072REF_VMP1_AS003	0.010195	0.0456	0.57	down	chr17:57916099-57916215
TI16709REF_U2AF1_AS007	0.011255	0.0484	0.57	down	chr21:44513765-44513912
TI18499REF_FAM65C_AS002	0.012448	0.0513	0.57	down	chr20:49242557-49242689
TI16121REF_NPAS2_AS013	0.013458	0.0546	0.57	down	chr2:101502951-101503042
TI21542REF_PPP2R2D_AS001	0.016562	0.0624	0.57	down	chr10:133770164-133770284
TI23209REF_EXOSC7_AS001	0.019004	0.0691	0.57	down	chr3:45043604-45043727
TI16473REF_MSTO1_AS005	0.024951	0.0831	0.57	down	chr1:155585766-155586074
TI22048REF_TNFAIP2_AS001	0.02527	0.0837	0.57	down	chr14:103596945-103597064
ASK05227REF_NRIP1_AS002	0.000234	0.00326	0.56	down	chr21:16336570-16337036
TI16080REF_MPRIP_AS011	0.000459	0.00541	0.56	down	chr17:17089522-17089622
TI16861REF_HIPK2_AS005	0.00056	0.00621	0.56	down	chr7:139248969-139249140
TI22002REF_EFNB2_AS001	0.000606	0.00659	0.56	down	chr13:107171013-107171124
TI16261REF_KIAA0182_AS007	0.00064	0.00684	0.56	down	chr16:85650716-85650842
TI18833REF_HERC4_AS002	0.000697	0.00718	0.56	down	chr10:69833498-69833592
TI16134REF_FARP1_AS005	0.000728	0.00737	0.56	down	chr13:99059096-99059450
ASK05238REF_DMAP1_AS002	0.000921	0.00887	0.56	down	chr1:44683967-44684296
TI16525REF_CCDC50_AS010	0.001364	0.0117	0.56	down	chr3:191112880-191113031
TI16169REF_ELL2_AS017	0.001828	0.0144	0.56	down	chr5:95221060-95221164
TI20841REF_RAI14_AS002	0.001935	0.0149	0.56	down	chr5:34771370-34771481
ASK03574REF	0.004535	0.0269	0.56	down	ENST00000532936
TI17624REF_EFNA5_AS002	0.009426	0.0433	0.56	down	chr5:106881942-106882044
TI01927NTNR	0.010884	0.0473	0.56	down	NR_027696
TI01928NTNR	0.010884	0.0473	0.56	down	NR_027697
ASK04750REF_TP53INP2_AS001	0.030068	0.0945	0.56	down	chr20:33298065-33298214
TI16126REF_HNRNPC_AS014	0.000106	0.00203	0.55	down	chr14:21717074-21717212
TI16160REF_TBC1D16_AS018	0.000155	0.00258	0.55	down	chr17:77907962-77908081
ASK05571REF_SERPINE2_AS00	0.000158	0.00258	0.55	down	chr2:224839928-224840556
TI16606REF_STC2_AS001	0.00022	0.0032	0.55	down	chr5:172749016-172749250
TI16344REF_KIAA1671_AS006	0.000306	0.00408	0.55	down	chr22:25592028-25592195
ASK04952REF_HOXB7_AS001	0.000526	0.00596	0.55	down	chr17:46687882-46688247
TI16715REF_FLNB_AS009	0.000545	0.0061	0.55	down	chr3:58118093-58118484
ASK04447REF	0.000657	0.00696	0.55	down	ENST00000466473
TI20645REF_BRWD1_AS001	0.000696	0.00718	0.55	down	chr21:40559911-40560058
ASK06591REF_ANTXR1_AS001	0.000742	0.00742	0.55	down	chr2:69476101-69476245
TI16621REF_GABRE_AS011	0.000805	0.00796	0.55	down	chrX:151126094-151126273

TI16254REF_CARS2_AS005	0.000841	0.00819	0.55	down	chr13:111319176-111319269
TI17186REF_IGF2BP1_AS004	0.001854	0.0145	0.55	down	chr17:47124902-47125047
TI17384REF_FBXW4_AS002	0.002018	0.0153	0.55	down	chr10:103447527-103447631
TI16169REF_ELL2_AS004	0.00222	0.016	0.55	down	chr5:95243182-95243299
ASK00822NTNR	0.002903	0.0195	0.55	down	XR_109900
ASK00989NTNR	0.003258	0.0212	0.55	down	NR_033680
TI17533REF_INSIG2_AS005	0.003576	0.0226	0.55	down	chr2:118857547-118857667
TI16134REF_FARP1_AS018	0.003685	0.0232	0.55	down	chr13:99058019-99058196
TI01849NTNR	0.006997	0.0357	0.55	down	XR_110300
TI01850NTNR	0.006997	0.0357	0.55	down	XR_133066
ASK06202REF_REP15_AS001	0.008463	0.0401	0.55	down	chr12:27849534-27849904
TI16126REF_HNRNPC_AS025	0.010196	0.0456	0.55	down	chr14:21727306-21727421
TI17911REF_MLPH_AS004	0.015988	0.0611	0.55	down	chr2:238398308-238398418
TI20835REF_ELOVL6_AS002	4.99E-05	0.00117	0.54	down	chr4:110968064-110968194
TI16621REF_GABRE_AS009	7.68E-05	0.00161	0.54	down	chrX:151140745-151141096
TI17606REF_RBPJ_AS004	0.000196	0.00301	0.54	down	chr4:26433690-26433839
ASK01217NTNR	0.000312	0.00412	0.54	down	NR_004858
TI18662REF_UGCG_AS003	0.000395	0.00483	0.54	down	chr9:114695892-114696075
TI19205REF_ZNF814_AS001	0.000512	0.00591	0.54	down	chr19:58382771-58382890
TI18937REF_C1RL_AS003	0.00216	0.0159	0.54	down	chr12:7251943-7252161
TI20152REF_QPRT_AS002	0.003047	0.0202	0.54	down	chr16:29707283-29707398
TI16134REF_FARP1_AS015	0.004773	0.0281	0.54	down	chr13:99052250-99052360
TI18544REF_ESYT3_AS004	0.004886	0.0285	0.54	down	chr3:138156346-138156498
TI16045REF_GRID1_AS032	0.024198	0.0812	0.54	down	chr10:87906283-87906379
TI16137REF_LPP_AS019	0.036202	0.108	0.54	down	chr3:188506184-188506287
TI16160REF_TBC1D16_AS015	6.41E-05	0.00141	0.53	down	chr17:77913536-77913721
TI19923REF_SPATS2_AS001	0.000105	0.00203	0.53	down	chr12:49890265-49890459
TI16080REF_MPRIP_AS023	0.000112	0.00212	0.53	down	chr17:17094551-17094867
TI16080REF_MPRIP_AS007	0.000206	0.00311	0.53	down	chr17:17091988-17092083
ASK00729NTNR	0.000395	0.00483	0.53	down	XR_111321
TI18416REF_KCTD15_AS003	0.000464	0.00542	0.53	down	chr19:34305806-34305966
TI16169REF_ELL2_AS015	0.000525	0.00596	0.53	down	chr5:95247812-95247977
TI16390REF_MGAT5_AS004	0.001426	0.0121	0.53	down	chr2:135207321-135207877
TI16134REF_FARP1_AS024	0.002106	0.0157	0.53	down	chr13:99055377-99055528
TI16968REF_VPS53_AS008	0.002125	0.0158	0.53	down	chr17:420009-420104
TI16121REF_NPAS2_AS020	0.018529	0.0677	0.53	down	chr2:101488115-101488247
TI17412REF_ISCU_AS001	0.033918	0.103	0.53	down	chr12:108956603-108956698
TI17288REF_UBXN7_AS006	4.07E-05	0.00101	0.52	down	chr3:196077819-196077984
ASK06877REF_SNAP25_AS001	9.93E-05	0.00202	0.52	down	chr20:10287139-10287888
ASK00625NTNR	0.000117	0.00218	0.52	down	NR_003716
TI17533REF_INSIG2_AS002	0.000119	0.00219	0.52	down	chr2:118861850-118861971
TI16621REF_GABRE_AS002	0.000148	0.00256	0.52	down	chrX:151140592-151140700
TI16452REF_SHANK3_AS013	0.000154	0.00258	0.52	down	chr22:51164405-51164547
ASK06320REF_CITED2_AS001	0.000154	0.00258	0.52	down	chr6:139693597-139693893
TI16080REF_MPRIP_AS029	0.000187	0.00291	0.52	down	chr17:17092197-17092430
TI19205REF_ZNF814_AS003	0.000216	0.00316	0.52	down	chr19:58381738-58381931
TI16080REF_MPRIP_AS004	0.000364	0.00457	0.52	down	chr17:17090770-17090955
TI17544REF_RTN4_AS006	0.000367	0.00457	0.52	down	chr2:55204292-55204435
ASK07035REF_MAP9_AS001	0.000811	0.00799	0.52	down	chr4:156267456-156267708
TI17921REF_SSFA2_AS005	0.001391	0.0118	0.52	down	chr2:182782841-182782957
TI16715REF_FLNB_AS005	0.001955	0.015	0.52	down	chr3:58117119-58117339
TI16080REF_MPRIP_AS008	0.003405	0.0217	0.52	down	chr17:17093304-17093561
TI16060REF_SKI_AS006	0.00561	0.031	0.52	down	chr1:2190852-2190957
TI21045REF_EPPK1_AS001	0.007021	0.0357	0.52	down	chr8:144947922-144948059
TI16606REF_STC2_AS003	3.70E-05	0.00097	0.51	down	chr5:172745553-172745701

TI16080REF_MPRIP_AS016	4.55E-05	0.00107	0.51		down	chr17:17093151-17093269
TI16035REF_MSI2_AS065	0.000157	0.00258	0.51		down	chr17:55757384-55757640
TI01353NTNR	0.000171	0.0027	0.51		down	NR_022008
TI16308REF_SON_AS013	0.000718	0.00732	0.51		down	chr21:34943556-34943759
TI16504REF_RAI1_AS008	0.001374	0.0118	0.51		down	chr17:17691789-17691894
TI17093REF_PBX1_AS007	0.001692	0.0137	0.51		down	chr1:164818509-164818620
TI16854REF_PHF10_AS005	0.005397	0.0306	0.51		down	chr6:170109676-170109781
TI16895REF_MYSM1_AS005	0.021598	0.0752	0.51		down	chr1:59125200-59125332
TI18051REF_LAMB1_AS003	0.000334	0.00431	0.5		down	chr7:107587910-107588011
ASK01124NTNR	0.000367	0.00457	0.5		down	NR_036488
TI23250REF_PPP4R2_AS001	0.002058	0.0154	0.5		down	chr3:73115490-73116038
TI16169REF_ELL2_AS020	0.003132	0.0207	0.5		down	chr5:95221960-95222327
TI17694REF_FUBP1_AS004	0.008071	0.039	0.5		down	chr1:78417464-78417658
TI16160REF_TBC1D16_AS002	1.90E-05	0.00063	0.49		down	chr17:77911055-77911246
ASK00955NTNR	2.73E-05	0.0008	0.49		down	NR_026570
ASK01047NTNR	3.11E-05	0.00086	0.49		down	XR_109114
TI17606REF_RBPJ_AS006	0.00012	0.00221	0.49		down	chr4:26435239-26435372
TI20513REF_NABP1_AS001	0.00021	0.00315	0.49		down	chr2:192547334-192547429
TI17205REF_DSG2_AS001	0.000213	0.00316	0.49		down	chr18:29127994-29128143
TI23096REF_MRPS6_AS001	0.000234	0.00326	0.49		down	chr21:35495996-35496121
TI18572REF_SPCS3_AS003	0.000446	0.00528	0.49		down	chr4:177251266-177251352
TI16134REF_FARP1_AS002	0.001006	0.00929	0.49		down	chr13:99056344-99056469
TI16169REF_ELL2_AS002	0.006144	0.0328	0.49		down	chr5:95221486-95221797
TI01707NTNR	0.013554	0.0547	0.49		down	NR_026757
TI18272REF_HIF1A_AS001	0.017316	0.0641	0.49		down	chr14:62207106-62207204
TI19272REF_COL6A3_AS001	0.022064	0.0761	0.49		down	chr2:238238929-238239046
TI19584REF_SAT1_AS003	1.79E-05	0.00061	0.48		down	chrX:23802024-23802141
TI11655REF	2.93E-05	0.00083	0.48		down	ENST00000564967
TI19584REF_SAT1_AS002	3.83E-05	0.00099	0.48		down	chrX:23802164-23802337
TI16080REF_MPRIP_AS021	4.04E-05	0.00101	0.48		down	chr17:17094320-17094460
TI16769REF_RPL27A_AS002	7.23E-05	0.00154	0.48		down	chr11:8709483-8709599
TI01924NTNR	0.000165	0.00265	0.48		down	XR_108329
TI01925NTNR	0.000165	0.00265	0.48		down	XR_108330
TI01487NTNR	0.000234	0.00326	0.48		down	XR_110657
ASK05870REF_PPP1R2_AS001	0.000268	0.00368	0.48		down	chr3:195241243-195241676
ASK05026REF_TRPV2_AS002	0.000276	0.00373	0.48		down	chr17:16328761-16331247
TI01668NTNR	0.000346	0.00442	0.48		down	XR_111050
ASK03300REF	0.000462	0.00542	0.48		down	ENST00000552877
TI16160REF_TBC1D16_AS010	0.000571	0.00628	0.48		down	chr17:77909671-77909849
ASK04645REF	0.00338	0.0217	0.48		down	ENST00000498479
TI02072NTNR	0.010653	0.0467	0.48		down	NR_037772
ASK06546REF_KLHL5_AS001	1.10E-05	0.00044	0.47		down	chr4:39114572-39128422
TI16080REF_MPRIP_AS017	1.55E-05	0.00056	0.47		down	chr17:17089167-17089262
TI16137REF_LPP_AS020	5.44E-05	0.00123	0.47		down	chr3:188606805-188607366
TI16606REF_STC2_AS006	0.000132	0.00234	0.47		down	chr5:172746236-172746325
TI20388REF_SAMD4B_AS002	0.006074	0.0326	0.47		down	chr19:39835101-39835211
ASK02854REF	1.46E-05	0.00055	0.46		down	ENST00000495451
TI19518REF_UBXN2B_AS002	4.43E-05	0.00107	0.46		down	chr8:59333740-59333882
TI17205REF_DSG2_AS004	6.15E-05	0.00136	0.46		down	chr18:29126843-29127338
ASK05026REF_TRPV2_AS001	0.000108	0.00207	0.46		down	chr17:16331582-16331927
ASK02853REF	0.000115	0.00216	0.46		down	ENST00000492361
ASK03153REF	0.000158	0.00258	0.46		down	ENST00000531102
TI18416REF_KCTD15_AS002	0.000723	0.00734	0.46		down	chr19:34305426-34305533
TI16330REF_RP11-31I10.4_AS00	0.001073	0.0098	0.46		down	chr16:1830621-1830714
TI16897REF_TOR1AIP2_AS004	0.004298	0.026	0.46		down	chr1:179832468-179832599

TI16169REF_ELL2_AS010	0.015232	0.0593	0.46	down	chr5:95225907-95226195
TI02024NTNR	8.10E-06	0.00038	0.45	down	NR_026656
TI16080REF_MPRIP_AS009	3.23E-05	0.00088	0.45	down	chr17:17065743-17065860
ASK07159REF_WNT5A_AS001	9.34E-05	0.00191	0.45	down	chr3:55501439-55501787
TI16885REF_SIKE1_AS005	0.000105	0.00203	0.45	down	chr1:115313063-115313155
TI16458REF_MYLK_AS011	0.000976	0.00916	0.45	down	chr3:123431211-123431306
TI16169REF_ELL2_AS011	0.000985	0.00916	0.45	down	chr5:95222812-95222918
TI16406REF_KCNAB2_AS007	0.001215	0.0108	0.45	down	chr1:6161130-6161253
TI16775REF_SCARB1_AS002	0.007574	0.0374	0.45	down	chr12:125266937-125267063
ASK05684REF_CLDN14_AS001	2.42E-05	0.00075	0.44	down	chr21:37832588-37832922
TI19382REF_PLOD2_AS002	4.23E-05	0.00103	0.44	down	chr3:145796801-145796893
TI18051REF_LAMB1_AS001	0.000123	0.00222	0.44	down	chr7:107580893-107581002
TI16087REF_HDLBP_AS018	0.000135	0.00236	0.44	down	chr2:242173029-242173197
ASK03026REF	0.000211	0.00315	0.44	down	ENST00000461647
TI17205REF_DSG2_AS006	0.001038	0.00951	0.44	down	chr18:29128176-29128319
ASK04411REF	9.30E-06	0.0004	0.43	down	ENST00000488355
TI17014REF_TIAM1_AS005	2.63E-05	0.00079	0.43	down	chr21:32494285-32494414
TI16080REF_MPRIP_AS018	0.000132	0.00234	0.43	down	chr17:17093792-17094092
TI16121REF_NPAS2_AS021	0.000232	0.00326	0.43	down	chr2:101474741-101474869
ASK07121REF_PCDH7_AS001	3.30E-06	0.00019	0.42	down	chr4:30723190-30723536
ASK04763REF_EDNRA_AS001	7.20E-06	0.00035	0.42	down	chr4:148406896-148407324
TI14844REF	1.10E-05	0.00044	0.42	down	ENST00000483977
TI16160REF_TBC1D16_AS022	3.00E-05	0.00083	0.42	down	chr17:77908856-77908988
TI16606REF_STC2_AS010	0.000585	0.00638	0.42	down	chr5:172749899-172750086
TI16715REF_FLNB_AS004	0.002497	0.0174	0.42	down	chr3:58117591-58117957
ASK01180NTNR	1.69E-05	0.00059	0.41	down	XR_110210
TI16160REF_TBC1D16_AS007	2.77E-05	0.0008	0.41	down	chr17:77907184-77907300
ASK02296REF	9.25E-05	0.00191	0.41	down	ENST00000531322
TI16508REF_NEDD4L_AS003	0.002208	0.016	0.41	down	chr18:56067367-56067503
TI16035REF_MSI2_AS001	0.003356	0.0216	0.41	down	chr17:55759829-55759954
TI16169REF_ELL2_AS018	0.005215	0.0299	0.41	down	chr5:95248025-95248150
ASK05703REF_DCC_AS001	3.00E-06	0.00018	0.4	down	chr18:50432403-50432673
ASK07153REF_EFEMP1_AS001	9.00E-06	0.0004	0.4	down	chr2:56093210-56093654
ASK00641NTNR	1.85E-05	0.00062	0.4	down	NR_037182
ASK02651REF	2.99E-05	0.00083	0.4	down	ENST00000446749
TI16621REF_GABRE_AS007	4.21E-05	0.00103	0.4	down	chrX:151125676-151126002
TI16196REF_SPG7_AS014	0.000441	0.00526	0.4	down	chr16:89589886-89589982
TI16080REF_MPRIP_AS026	4.10E-06	0.00023	0.39	down	chr17:17095201-17095471
TI16344REF_KIAA1671_AS012	9.20E-06	0.0004	0.39	down	chr22:25591568-25591750
TI16080REF_MPRIP_AS024	1.70E-05	0.00059	0.39	down	chr17:17095543-17095635
TI19382REF_PLOD2_AS003	3.94E-05	0.001	0.39	down	chr3:145795418-145795515
TI16169REF_ELL2_AS022	0.000409	0.00496	0.39	down	chr5:95222989-95223085
TI16769REF_RPL27A_AS005	0.000978	0.00916	0.39	down	chr11:8709837-8709938
TI22794REF_ID2_AS001	9.70E-06	0.0004	0.38	down	chr2:8823377-8823484
TI16169REF_ELL2_AS013	5.43E-05	0.00123	0.38	down	chr5:95222562-95222695
TI19295REF_RP5-977B1.10_AS0(	5.05E-05	0.00117	0.37	down	chr20:35240250-35240344
ASK06740REF_KANK1_AS001	0.000548	0.0061	0.37	down	chr9:745609-746075
ASK03656REF	3.10E-06	0.00018	0.36	down	ENST00000535681
TI16772REF_IFITM10_AS009	4.47E-05	0.00107	0.36	down	chr11:1769024-1769111
TI16809REF_TYMS_AS006	0.000105	0.00203	0.36	down	chr18:666201-666327
ASK07183REF_RP1-170O19.20_/	1.10E-06	9.68E-05	0.35	down	chr7:27208749-27209356
TI10837REF	1.78E-05	0.00061	0.35	down	ENST00000478436
ASK05491REF_HHIP_AS001	2.48E-05	0.00076	0.35	down	chr4:145573877-145582494
TI17538REF_FMNL2_AS004	2.74E-05	0.0008	0.35	down	chr2:153475906-153476005
ASK05904REF_KDM5D_AS001	4.54E-05	0.00107	0.35	down	chrY:21906676-21906823

TI16508REF_NEDD4L_AS008	0.000127	0.00228	0.34	down	chr18:56067061-56067165
TI19382REF_PLOD2_AS001	0.000585	0.00638	0.34	down	chr3:145794877-145795091
TI16344REF_KIAA1671_AS005	7.70E-06	0.00036	0.33	down	chr22:25592261-25592370
ASK03240REF	1.21E-05	0.00047	0.33	down	ENST00000546199
TI16508REF_NEDD4L_AS004	0.000168	0.00268	0.33	down	chr18:56066419-56066554
ASK04127REF	4.00E-07	4.80E-05	0.32	down	ENST00000465659
ASK06442REF_PRUNE2_AS001	1.30E-06	0.00011	0.32	down	chr9:79319764-79320231
ASK03993REF	2.20E-06	0.00016	0.32	down	ENST00000464589
ASK03363REF	2.10E-05	0.00068	0.32	down	ENST00000556245
ASK07010REF_SSPN_AS001	2.50E-06	0.00016	0.31	down	chr12:26384373-26384682
TI16395REF_SIK1_AS011	2.70E-06	0.00017	0.31	down	chr21:44844404-44844578
TI16344REF_KIAA1671_AS004	4.00E-07	4.80E-05	0.3	down	chr22:25589738-25589920
TI23098REF_ADAMTS1_AS001	1.00E-06	9.10E-05	0.3	down	chr21:28215535-28215651
ASK06078REF_GALNT3_AS001	2.90E-06	0.00018	0.3	down	chr2:166603381-166606460
TI22142REF_NR2F2_AS001	1.38E-05	0.00053	0.29	down	chr15:96875882-96876047
TI16344REF_KIAA1671_AS014	2.50E-06	0.00016	0.28	down	chr22:25590367-25590565
ASK00919NTNR	1.00E-07	1.65E-05	0.27	down	NR_033660
ASK05666REF_CNR1_AS001	4.00E-07	4.80E-05	0.27	down	chr6:88852118-88852664
TI16344REF_KIAA1671_AS010	2.24E-05	0.00071	0.27	down	chr22:25590928-25591095
TI20152REF_QPRT_AS001	5.00E-06	0.00026	0.26	down	chr16:29709405-29709540
ASK03812REF	2.40E-06	0.00016	0.25	down	ENST00000472142
ASK05048REF_SLC37A2_AS002	5.40E-06	0.00028	0.25	down	chr11:124958026-124958272
ASK06050REF_HOXA11_AS001	7.00E-07	6.60E-05	0.24	down	chr7:27224179-27224600
ASK00799NTNR	7.00E-07	6.60E-05	0.23	down	NR_037595
TI16134REF_FARP1_AS017	0.000293	0.00393	0.23	down	chr13:99053706-99053877
ASK05689REF_RAB27B_AS001	1.00E-07	1.65E-05	0.22	down	chr18:52555299-52565289
ASK02878REF	1.30E-06	0.00011	0.22	down	ENST00000491263
TI16395REF_SIK1_AS003	7.50E-06	0.00036	0.22	down	chr21:44843900-44844078
TI16930REF_KRT8_AS002	9.20E-06	0.0004	0.21	down	chr12:53341932-53342217
TI19679REF_TBX15_AS001	6.56E-05	0.00142	0.2	down	chr1:119526285-119526380
ASK02729REF	1.00E-07	1.00E-07	0.19	down	ENST00000514040
ASK07356REF_HSD17B2_AS001	5.00E-07	5.50E-05	0.19	down	chr16:82104411-82104787
ASK06946REF_ST18_AS001	1.00E-07	1.65E-05	0.16	down	chr8:53038283-53038696
ASK06043REF_TSPAN13_AS001	1.00E-07	1.00E-07	0.12	down	chr7:16823650-16824073
ASK02952REF	1.00E-07	1.65E-05	0.12	down	ENST00000470170
ASK05042REF_EFNB2_AS002	1.00E-07	1.00E-07	0.11	down	chr13:107144703-107145374
ASK00892NTNR	1.00E-07	1.00E-07	0.086	down	NR_037939
NC00354FR	1.00E-07	1.00E-07	0.041	down	FR240648

Supplementary table 2.differentially expressed mRNA in BMSCs and PDLSCs

ProbeSet	p-value	FDR	PDLSCs/BMSCs	style	gene_symbol
TC1700360	0.0000001	0.0000001	14.17	up	CCL2
TC0501120	0.0000001	0.0000001	12.43	up	HAPLN1
TC0X00009	0.0000001	0.0000001	9.45	up	XG
TC0501080	0.0000001	0.0000001	9.37	up	F2RL2
TC0X00740	0.0000001	0.0000001	7.93	up	MXRA5
TC1400180	0.0000001	0.0000001	7.88	up	PAX9
TC1200714	0.0000001	0.0000001	7.84	up	OAS2
TC0400318	0.0000001	0.00000202	7.55	up	EPGN
TC1600650	0.0000012	0.0000129	7.24	up	FOXF1
TC1000844	0.0000009	0.0000102	6.79	up	MKX
TC0600008	0.0000004	0.00000586	6.71	up	FOXF2
TC0601604	0.0000001	0.0000001	5.86	up	MOXD1
TC0202302	0.0000001	0.0000001	5.81	up	IGFBP5
TC0500310	0.0000001	0.0000001	5.77	up	RASGRF2
TC0901145	0.0000001	0.0000001	5.72	up	BARX1
TC2000071	0.0000001	0.0000001	5.37	up	PLCB4
TC2200354	0.0000001	0.0000001	5.31	up	FBLN1
TC0400315	0.0000001	0.00000202	5.1	up	CXCL1
TC0501259	0.0000001	0.0000001	4.97	up	FBN2
TC0801026	0.0000007	0.00000852	4.88	up	TRPA1
TC0800962	0.0000001	0.0000001	4.64	up	TOX
TC0700158	0.0000001	0.0000001	4.54	up	AQP1
TC0300800	0.0000001	0.0000001	4.49	up	NLGN1
TC1200075	0.0000001	0.0000001	4.47	up	C1S
TC1200712	0.0000001	0.0000001	4.33	up	OAS1
TC0500174	0.0000001	0.0000001	4.24	up	FST
TC0900435	0.0000021	0.0000188	4.19	up	NR4A3
TC0X00989	0.0000001	0.0000001	4.15	up	EDA2R
TC1800290	0.0000001	0.0000001	4.14	up	COLEC12
TC0401288	0.0000001	0.00000202	4.1	up	RNF150
TC2000628	0.0000019	0.0000175	4.05	up	CST4
TC0901186	0.0000001	0.00000202	4.02	up	GABBR2
TC1901095	0.0000001	0.0000001	4.01	up	C3
TC0900529	0.0000001	0.0000001	4	up	PTGS1
TC1200713	0.0000001	0.00000202	3.99	up	OAS3
TC1600899	0.0000001	0.0000001	3.88	up	XYLT1
TC1200047	0.0000001	0.0000001	3.87	up	NTF3
TC0202309	0.0000001	0.0000001	3.74	up	TNS1
TC1000629	0.0000001	0.0000001	3.68	up	GRK5
TC2000398	0.0000014	0.0000143	3.58	up	TFAP2C
TC0500282	0.0000004	0.00000586	3.56	up	F2R

TC1200919	0.0000001	0.00000202	3.47	up	C1R
TC0801212	0.0000001	0.0000001	3.46	up	ENPP2
TC1500323	0.0000007	0.00000852	3.42	up	FGF7
TC0900624	0.0000002	0.00000349	3.42	up	PRRX2
TC0500638	0.0000001	0.00000202	3.39	up	SGCD
TC0400312	0.0000004	0.00000586	3.35	up	IL8
TC0701417	0.0000006	0.00000769	3.35	up	SAMD9
TC1001179	0.0000001	0.0000001	3.33	up	PDLIM1
TC0600502	0.0000026	0.0000217	3.31	up	KCNQ5
TC0400231	0.0000001	0.0000001	3.29	up	PDGFRA
TC0100626	0.0000003	0.00000475	3.27	up	LEPROT
TC0401221	0.0000023	0.0000199	3.21	up	PDE5A
TC0100679	0.000009	0.0000516	3.16	up	IFI44
TC0100656	0.0000019	0.0000175	3.15	up	LHX8
TC0X00545	0.0000023	0.0000199	3.14	up	GRIA3
TC2200020	0.0000005	0.00000688	3.14	up	USP18
TC1100240	0.0000066	0.0000417	3.1	up	ANO3
TC1701235	0.0000002	0.00000349	3.1	up	SLFN11
TC0800311	0.0000002	0.00000349	3.08	up	LYN
TC0300664	0.0000001	0.0000001	3.07	up	CLSTN2
TC0100808	0.0000028	0.000023	3.06	up	GSTM1
TC0901096	0.0000011	0.0000121	3.03	up	GAS1
TC1101830	0.0000004	0.00000586	2.93	up	CRYAB
TC1500435	0.0000003	0.00000475	2.93	up	PAQR5
TC0202202	0.0000007	0.00000852	2.93	up	RFTN2
TC1201537	0.0000006	0.00000769	2.93	up	TMEM119
TC0901277	0.0000004	0.00000586	2.92	up	TNFSF15
TC1200492	0.0000004	0.00000586	2.9	up	IRAK3
TC0400051	0.0000872	0.00031	2.86	up	MSX1
TC1800511	0.0000001	0.0000001	2.81	up	CCBE1
TC0200349	0.0000001	0.00000202	2.8	up	ACTG2
TC1400899	0.0000001	0.0000001	2.79	up	SIX1
TC0901020	0.0002737	0.000822	2.78	up	Q59F31_HUMAN
TC2200523	0.0000003	0.00000475	2.77	up	GSTT1
TC0600743	0.0000002	0.00000349	2.77	up	PDE7B
TC1700076	0.0000001	0.0000001	2.77	up	XAF1
TC1700140	0.0000066	0.0000417	2.75	up	MYOCD
TC1101566	0.0000001	0.0000001	2.73	up	CD248
TC0800779	0.0000008	0.00000948	2.73	up	CSGALNACT1
TC0X01163	0.0000004	0.00000586	2.68	up	AMOT
TC0500723	0.0000001	0.00000202	2.65	up	MSX2
TC1700661	0.0000001	0.0000001	2.65	up	TBX2
TC0200019	0.0000001	0.0000001	2.62	up	RSAD2
TC0800567	0.0000087	0.0000503	2.62	up	WISP1

TC1200015	0.0000001	0.0000001	2.61	up	CACNA1C
TC0600731	0.0000026	0.0000217	2.59	up	EYA4
TC0601557	0.0000001	0.00000202	2.59	up	FAM162B
TC0201785	0.0000022	0.0000192	2.57	up	TBC1D8
TC1101777	0.0000084	0.0000494	2.53	up	MMP12
TC0700616	0.0000001	0.00000202	2.52	up	LRRN3
TC0500681	0.0000003	0.00000475	2.52	up	WWC1
TC1200048	0.0000001	0.0000001	2.49	up	CD9
TC1201535	0.0000002	0.00000349	2.49	up	CMKLR1
TC1300555	0.0000004	0.00000586	2.48	up	KCTD12
TC1401102	0.0000001	0.00000202	2.47	up	LINC00341
TC2200553	0.0000006	0.00000769	2.46	up	MN1
TC0800107	0.0000001	0.00000202	2.45	up	SLC7A2
TC0102731	0.0000002	0.00000349	2.44	up	CTSK
TC0202191	0.0000116	0.0000625	2.42	up	HECW2
TC0102037	0.0000009	0.0000102	2.42	up	IFI6
TC1201525	0.0000056	0.000037	2.37	up	APPL2
TC0700481	0.0000001	0.00000202	2.37	up	GNG11
TC2100142	0.0000001	0.0000001	2.37	up	MX1
TC1100144	0.0002055	0.000644	2.34	up	OLFML1
TC1800144	0.0000031	0.0000246	2.33	up	MOCOS
TC0800897	0.0000015	0.0000148	2.32	up	SFRP1
TC0700089	0.0000016	0.0000155	2.31	up	AHR
TC0701680	0.0000076	0.0000458	2.31	up	PARP12
TC1001328	0.0000001	0.00000202	2.3	up	CPXM2
TC0500141	0.000001	0.0000112	2.3	up	PTGER4
TC1200945	0.0000007	0.00000852	2.29	up	MFAP5
TC1501189	0.0000003	0.00000475	2.27	up	HOMER2
TC0800459	0.0000939	0.00033	2.27	up	OSR2
TC0100275	0.0000005	0.00000688	2.26	up	MAN1C1
TC0600186	0.0000006	0.00000769	2.25	up	HIST1H2BM
TC1000554	0.0000128	0.0000677	2.25	up	INA
TC0101513	0.0000076	0.0000458	2.25	up	KCNK2
TC0701053	0.0000074	0.000045	2.25	up	LOC541472
TC1600627	0.0000002	0.00000349	2.24	up	CDH13
TC0700220	0.0000001	0.00000202	2.23	up	AEBP1
TC0501071	0.0000138	0.0000714	2.23	up	GCNT4
TC0401264	0.0000007	0.00000852	2.23	up	PCDH18
TC1201615	0.0000008	0.00000948	2.22	up	CIT
TC1100010	0.000001	0.0000112	2.22	up	IFITM1
TC0200169	0.0000003	0.00000475	2.21	up	LTBP1
TC0500643	0.0000009	0.0000102	2.2	up	CYFIP2
TC0800222	0.0000002	0.00000349	2.2	up	GPR124
TC1101775	0.0000001	0.00000202	2.2	up	MMP1

TC1700605	0.0000035	0.0000268	2.2	up	TOM1L1
TC1701647	0.0000109	0.0000593	2.19	up	ABCA6
TC0X00307	0.0000006	0.00000769	2.19	up	HEPH
TC0900283	0.0000005	0.00000688	2.19	up	TJP2
TC0801210	0.0000004	0.00000586	2.19	up	TNFRSF11B
TC1400465	0.0000494	0.000198	2.18	up	PPP4R4
TC0701144	0.0000034	0.0000265	2.17	up	AMPH
TC0201409	0.0000005	0.00000688	2.17	up	GALNT14
TC1200362	0.0000001	0.00000202	2.17	up	IGFBP6
TC0X00164	0.0000031	0.0000246	2.17	up	MAOA
TC0101660	0.0000136	0.0000706	2.16	up	FMN2
TC1201442	0.000001	0.0000112	2.14	up	DCN
TC0202184	0.0000065	0.0000414	2.14	up	SDPR
TC1800051	0.0000037	0.0000278	2.12	up	APCDD1
TC0700804	0.0000004	0.00000586	2.11	up	EPHB6
TC0201998	0.0000004	0.00000586	2.11	up	RND3
TC0600734	0.000049	0.000197	2.11	up	TCF21
TC0601023	0.0000091	0.0000521	2.1	up	HIST2H4A
TC0401141	0.0000015	0.0000148	2.08	up	ADH1C
TC1701244	0.0000365	0.000158	2.08	up	CCL5
TC0901131	0.0000001	0.00000202	2.08	up	OMD
TC2200448	0.0000837	0.0003	2.08	up	USP41
TC1100276	0.0000016	0.0000155	2.06	up	FJX1
TC0101393	0.0000018	0.0000169	2.06	up	NR5A2
TC1400762	0.0000003	0.00000475	2.06	up	PRKD1
TC0200918	0.0000389	0.000166	2.06	up	ZNF804A
TC1000789	0.0000053	0.0000355	2.05	up	ITGA8
TC0800108	0.0000022	0.0000192	2.05	up	PDGFRL
TC1201104	0.0001698	0.000548	2.04	up	CPNE8
TC0102283	0.0000003	0.00000475	2.03	up	DHCR24
TC0800172	0.0000112	0.0000604	2.03	up	ESCO2
TC1000856	0.0000072	0.0000442	2.03	up	KIAA1462
TC1500808	0.000005	0.0000341	2.03	up	LPCAT4
TC1100273	0.0000002	0.00000349	2.02	up	EHF
TC1300369	0.0000277	0.000128	2.02	up	FLT1
TC0600134	0.0000099	0.0000555	2.02	up	LRRC16A
TC0101906	0.0000153	0.0000774	2.02	up	TMEM16M
TC1000918	0.0000087	0.0000503	2.01	up	CXCL12
TC0501405	0.0000002	0.00000349	2	up	PDGFRB
TC0X00073	0.0000528	0.000209	2	up	SCML1
TC0700156	0.0000004	0.00000586	1.99	up	INMT
TC0400949	0.0000009	0.0000102	1.99	up	SCFD2
TC0500106	0.0000016	0.0000155	1.98	up	C5orf23
TC0202052	0.0000052	0.0000351	1.97	up	IFIH1

TC1600639	0.000008	0.0000475	1.96	up	CRISPLD2
TC0X00744	0.0000212	0.000102	1.96	up	LOC389906
TC0201067	0.0000248	0.000117	1.95	up	CPS1
TC1200558	0.0000476	0.000192	1.94	up	ALX1
TC1100793	0.0000053	0.0000355	1.94	up	BIRC3
TC0700107	0.0000057	0.0000373	1.94	up	IL6
TC0801025	0.0000231	0.00011	1.94	up	MSC
TC1700284	0.0000042	0.0000304	1.94	up	TMEM97
TC0800581	0.0000079	0.0000472	1.93	up	DENND3
TC0100812	0.0000112	0.0000604	1.93	up	GSTM5
TC1101776	0.0000022	0.0000192	1.93	up	MMP3
TC1500271	0.0000133	0.0000697	1.93	up	TMEM62
TC1800335	0.0000039	0.0000288	1.92	up	CR030_HUMAN
TC0701658	0.0000005	0.00000688	1.92	up	FAM180A
TC0100678	0.0000087	0.0000503	1.92	up	IFI44L
TC1100196	0.0000006	0.00000769	1.92	up	INSC
TC1101613	0.0000037	0.0000278	1.91	up	CPT1A
TC1400414	0.0000803	0.000292	1.91	up	FLRT2
TC1300029	0.000005	0.0000341	1.9	up	SPATA13
TC0901239	0.0000015	0.0000148	1.9	up	SVEP1
TC2200752	0.0001802	0.000577	1.89	up	Q5H950_HUMAN
TC1500525	0.0000843	0.000302	1.88	up	CHRNA5
TC0401390	0.0001644	0.000535	1.88	up	DDX60
TC1400716	0.0000009	0.0000102	1.88	up	SLC7A8
TC1300290	0.0000003	0.00000475	1.87	up	F10
TC2000720	0.0000017	0.0000162	1.87	up	SAMHD1
TC0200784	0.0000039	0.0000288	1.87	up	TNFAIP6
TC1900313	0.000003	0.0000242	1.86	up	ARRDC2
TC0103401	0.0001112	0.000382	1.86	up	GREM2
TC1300409	0.0001631	0.000532	1.86	up	TRPC4
TC0201856	0.0006612	0.00177	1.85	up	EN1
TC0301838	0.0001537	0.000506	1.85	up	MASP1
TC0101333	0.0000102	0.0000565	1.85	up	MR1
TC0500105	0.0000009	0.0000102	1.85	up	NPR3
TC0901183	0.0000191	0.000093	1.85	up	TRIM14
TC0400522	0.0001881	0.000598	1.84	up	HSPA4L
TC1201283	0.0000072	0.0000442	1.84	up	MMP19
TC0600789	0.000012	0.0000643	1.84	up	STXBP5
TC0103161	0.0003597	0.00104	1.83	up	NUAK2
TC0400334	0.0000011	0.0000121	1.83	up	SHROOM3
TC0201480	0.000005	0.0000341	1.83	up	SIX2
TC0X00308	0.0000089	0.0000513	1.82	up	AR
TC01r00012	0.0000028	0.000023	1.82	up	GCUD2
TC0202407	0.0000058	0.0000378	1.82	up	HJURP

TC1600352	0.0000041	0.0000298	1.82	up	HSD3B7
TC1100163	0.0000144	0.0000742	1.82	up	WEE1
TC0601703	0.0000379	0.000163	1.81	up	CNKSR3
TC0300219	0.0001708	0.000549	1.81	up	KIF15
TC0102787	0.000037	0.00016	1.8	up	S100A4
TC1401103	0.0000027	0.0000225	1.79	up	C14orf49
TC0101641	0.0000015	0.0000148	1.79	up	EDARADD
TC1300438	0.0000145	0.0000744	1.79	up	EPSTI1
TC1400464	0.000012	0.0000643	1.79	up	IFI27
TC0500171	0.0000178	0.0000881	1.79	up	ITGA2
TC0103196	0.0000003	0.00000475	1.79	up	PLXNA2
TC0301327	0.0000009	0.0000102	1.78	up	LRIG1
TC0901054	0.000111	0.000382	1.78	up	RFK
TC0801115	0.0000029	0.0000237	1.78	up	RUNX1T1
TC0102432	0.000004	0.0000293	1.78	up	TGFBR3
TC1101005	0.0000052	0.0000351	1.77	up	APLP2
TC1500935	0.0003651	0.00105	1.77	up	CEP152
TC0500854	0.0000013	0.0000136	1.77	up	CTNND2
TC0301402	0.0000021	0.0000188	1.76	up	ABI3BP
TC0400339	0.0000034	0.0000265	1.76	up	FRAS1
TC2200744	0.000003	0.0000242	1.76	up	KIAA1644
TC0201468	0.0000389	0.000166	1.75	up	ZFP36L2
TC0901163	0.0000015	0.0000148	1.74	up	AAED1
TC0X00062	0.0001456	0.000484	1.74	up	CA5B
TC0400389	0.0000129	0.0000682	1.74	up	HERC6
TC1101031	0.0000048	0.0000334	1.74	up	IFITM3
TC0301349	0.0000006	0.00000769	1.74	up	PDZRN3
TC0800378	0.0000478	0.000193	1.74	up	RDH10
TC1501224	0.0000006	0.00000769	1.73	up	MFGE8
TC1400251	0.0000035	0.0000268	1.73	up	PELI2
TC1800336	0.0000017	0.0000162	1.73	up	PIEZO2
TC0101975	0.0000059	0.0000382	1.73	up	TCEA3
TC0201986	0.0000039	0.0000288	1.73	up	ZEB2
TC2200281	0.0000038	0.0000283	1.72	up	APOBEC3B
TC1500545	0.000001	0.0000112	1.72	up	KIAA1199
TC1800513	0.0004324	0.00123	1.72	up	MC4R
TC1500806	0.0000956	0.000334	1.72	up	SLC12A6
TC0102861	0.0000006	0.00000769	1.71	up	NES
TC0300742	0.0000015	0.0000148	1.71	up	PTX3
TC0X00839	0.0000017	0.0000162	1.71	up	TMEM47
TC0400693	0.0003214	0.000944	1.7	up	NEIL3
TC0401096	0.0000026	0.0000217	1.7	up	NKX6-1
TC0102696	0.0023503	0.0054	1.7	up	Q5TEC7_HUMAN
TC1300566	0.0000465	0.000189	1.7	up	SPRY2

TC0701422	0.0000024	0.0000204	1.7	up	TFPI2
TC0500658	0.000053	0.000209	1.69	up	ADRA1B
TC0102858	0.0000059	0.0000382	1.69	up	IQGAP3
TC0X00657	0.0000019	0.0000175	1.69	up	MAMLD1
TC1101616	0.0000044	0.0000313	1.69	up	MRGPRF
TC1000181	0.0000017	0.0000162	1.69	up	Q96IL8_HUMAN
TC0301054	0.0000279	0.000128	1.69	up	SGOL1
TC0601723	0.0000386	0.000166	1.69	up	SOD2
TC0401419	0.0000669	0.000251	1.68	up	ASB5
TC1400236	0.000058	0.000225	1.68	up	CDKN3
TC0501384	0.0000181	0.0000892	1.68	up	DPYSL3
TC0101017	0.0000006	0.00000769	1.68	up	ECM1
TC1100761	0.0000036	0.0000273	1.68	up	PANX1
TC0101434	0.0004562	0.00128	1.68	up	PRELP
TC1400847	0.0000111	0.0000602	1.68	up	SAV1
TC0701387	0.0000606	0.000233	1.68	up	SEMA3A
TC1200626	0.0000043	0.0000308	1.67	up	ANO4
TC1400867	0.00006	0.000232	1.67	up	BMP4
TC0100467	0.0000073	0.0000446	1.67	up	CDC20
TC1300164	0.0000626	0.000238	1.67	up	CKAP2
TC0700995	0.0000031	0.0000246	1.67	up	CYTH3
TC2000339	0.0000018	0.0000169	1.67	up	EYA2
TC1000778	0.0000048	0.0000334	1.67	up	FAM107B
TC1000064	0.0000056	0.000037	1.67	up	MCM10
TC0401450	0.0001846	0.000589	1.67	up	MLF1IP
TC0800196	0.0000477	0.000193	1.67	up	NRG1
TC0100860	0.0000017	0.0000162	1.67	up	OLFML3
TC0202204	0.0000009	0.0000102	1.67	up	SATB2
TC0102862	0.0000044	0.0000313	1.66	up	CRABP2
TC0701189	0.0000104	0.0000573	1.66	up	IGFBP3
TC0900458	0.0000076	0.0000458	1.66	up	SLC44A1
TC2000043	0.0000012	0.0000129	1.65	up	CDC25B
TC0701056	0.0000845	0.000302	1.65	up	FAM126A
TC0100897	0.0001159	0.000396	1.65	up	FAM72B
TC0201005	0.0000919	0.000324	1.65	up	FZD7
TC0103049	0.00001	0.0000559	1.65	up	GLUL
TC1700037	0.0000008	0.00000948	1.65	up	GSG2
TC0103203	0.0006637	0.00177	1.64	up	C1orf133
TC0600007	0.0000598	0.000231	1.64	up	FOXQ1
TC0900040	0.0000183	0.0000897	1.64	up	PDCD1LG2
TC0701418	0.0000048	0.0000334	1.64	up	SAMD9L
TC1101899	0.0000016	0.0000155	1.64	up	THY1
TC0301218	0.0000029	0.0000237	1.64	up	UBA7
TC1300193	0.0008075	0.00211	1.63	up	BORA

TC0201830	0.0000106	0.0000581	1.63	up	CKAP2L
TC0500936	0.0000069	0.000043	1.63	up	DAB2
TC2200665	0.0000754	0.000276	1.63	up	DMC1
TC1001141	0.0000008	0.00000948	1.62	up	ACTA2
TC0201439	0.0000041	0.0000298	1.62	up	CYP1B1
TC1100415	0.0055053	0.0116	1.62	up	FAM111B
TC1201637	0.0000493	0.000197	1.62	up	OASL
TC0401212	0.0001904	0.000604	1.62	up	PRSS12
TC0801284	0.00004	0.000169	1.62	up	PTK2
TC0200413	0.0000639	0.000242	1.62	up	VAMP5
TC2000265	0.0000564	0.00022	1.61	up	FAM83D
TC1401075	0.0000015	0.0000148	1.61	up	FBLN5
TC0102711	0.0000055	0.0000365	1.61	up	HIST2H2AB
TC1000433	0.0013197	0.00324	1.61	up	KIF20B
TC0201337	0.000013	0.0000684	1.61	up	OSR1
TC1400179	0.0013143	0.00323	1.61	up	Q96HD6_HUMAN
TC0202070	0.0000479	0.000193	1.61	up	SCN9A
TC0500593	0.0000033	0.0000259	1.6	up	ABLIM3
TC0400351	0.0000071	0.0000439	1.6	up	FGF5
TC0X01076	0.000635	0.00171	1.6	up	NAP1L3
TC1001078	0.0000156	0.0000785	1.6	up	NDST2
TC0X00346	0.0001982	0.000625	1.6	up	NHSL2
TC1700880	0.0000024	0.0000204	1.6	up	NXN
TC0400523	0.0005637	0.00153	1.6	up	PLK4
TC1400838	0.0000038	0.0000283	1.59	up	C14orf182
TC0102290	0.0000013	0.0000136	1.59	up	PPAP2B
TC1300328	0.0001379	0.000464	1.59	up	SKA3
TC0400929	0.000568	0.00154	1.59	up	TEC
TC0103126	0.0000153	0.0000774	1.59	up	UBE2T
TC1400476	0.0007376	0.00194	1.58	up	BDKRB2
TC0700721	0.0000124	0.0000661	1.58	up	BPGM
TC0400352	0.0019356	0.00453	1.58	up	C4orf22
TC1000796	0.0000085	0.0000497	1.58	up	CUBN
TC1201214	0.0000186	0.0000909	1.58	up	GALNT6
TC0401052	0.0000254	0.000119	1.58	up	NAAA
TC0300463	0.0000151	0.0000767	1.58	up	NFKBIZ
TC0102695	0.0007057	0.00187	1.58	up	NM_001025303
TC1400531	0.0462578	0.0771	1.58	up	NR_003198.1
TC0300556	0.0000538	0.000211	1.58	up	PARP14
TC0700411	0.0002302	0.000709	1.58	up	SNORA14A
TC1600239	0.0002432	0.000742	1.58	up	TMEM159
TC0X00032	0.0000136	0.0000706	1.58	up	WWC3
TC1901223	0.0000079	0.0000472	1.57	up	CACNA1A
TC0100402	0.0000538	0.000211	1.57	up	CDCA8

TC0200862	0.0000043	0.0000308	1.57	up	DLX1
TC0501493	0.0000156	0.0000785	1.57	up	DUSP1
TC21r00006	0.0000692	0.000257	1.57	up	LOC389833
TC1101310	0.0000094	0.0000535	1.57	up	PAMR1
TC1101263	0.0008696	0.00225	1.57	up	SVIP
TC0500594	0.0000046	0.0000326	1.56	up	AFAP1L1
TC0103096	0.0003188	0.000938	1.56	up	ASPM
TC1201444	0.0000822	0.000297	1.56	up	BTG1
TC0400592	0.0000447	0.000185	1.56	up	DCLK2
TC0301339	0.0000209	0.000101	1.56	up	FOXP1
TC0100486	0.0001703	0.000549	1.56	up	KIF2C
TC0300289	0.0000053	0.0000355	1.56	up	MAPKAPK3
TC0600990	0.0000287	0.000131	1.56	up	MBOAT1
TC1500253	0.0000843	0.000302	1.56	up	NUSAP1
TC0301636	0.0000033	0.0000259	1.56	up	PLSCR4
TC0600523	0.0024146	0.00553	1.56	up	TTK
TC1500238	0.0005042	0.00139	1.55	up	CASC5
TC0200993	0.0001461	0.000484	1.55	up	CASP10
TC0103248	0.0000404	0.00017	1.55	up	DUSP10
TC0X00592	0.0000048	0.0000334	1.55	up	FHL1
TC2200262	0.0009546	0.00244	1.55	up	H1F0
TC1701328	0.0000028	0.000023	1.55	up	KRTAP1-5
TC0701070	0.0007326	0.00193	1.55	up	OSBPL3
TC1500184	0.0007118	0.00188	1.54	up	ARHGAP11A
TC0200868	0.0000232	0.00011	1.54	up	CDCA7
TC1101252	0.0000102	0.0000565	1.54	up	E2F8
TC0102067	0.0000235	0.000111	1.54	up	FABP3
TC1000425	0.0000034	0.0000265	1.54	up	FAS
TC1901638	0.0000155	0.0000782	1.54	up	MEIS3
TC2100141	0.0000344	0.000151	1.54	up	MX2
TC0800523	0.0000099	0.0000555	1.54	up	NOV
TC0800192	0.0000312	0.00014	1.54	up	NRG1
TC0600107	0.0000213	0.000102	1.54	up	RNF144B
TC0100845	0.0000163	0.0000818	1.54	up	WNT2B
TC1500272	0.000033	0.000147	1.53	up	CCNDBP1
TC0401171	0.0000593	0.00023	1.53	up	DKK2
TC2100125	0.0000771	0.000281	1.53	up	ETS2
TC0300068	0.0000572	0.000223	1.53	up	FBLN2
TC0601696	0.0006927	0.00184	1.53	up	FBXO5
TC0301400	0.0000031	0.0000246	1.53	up	FILIP1L
TC0400226	0.000139	0.000466	1.53	up	FIP1L1
TC1200625	0.0004782	0.00133	1.53	up	GAS2L3
TC1101434	0.0030053	0.00673	1.53	up	GLYATL2
TC1101801	0.0003885	0.00111	1.53	up	GUCY1A2

TC1000427	0.0000265	0.000123	1.53	up	IFIT3
TC1500663	0.0000117	0.0000628	1.53	up	IGF1R
TC1600236	0.0004384	0.00124	1.53	up	LOC81691
TC0800594	0.0000021	0.0000188	1.53	up	LY6E
TC1400790	0.0000035	0.0000268	1.53	up	NFKBIA
TC0501480	0.0000022	0.0000192	1.53	up	SLIT3
TC0600754	0.0000021	0.0000188	1.53	up	TNFAIP3
TC2000629	0.0048288	0.0104	1.52	up	CST2
TC1200367	0.0000206	0.0000997	1.52	up	ESPL1
TC1600653	0.000004	0.0000293	1.52	up	FOXL1
TC0103109	0.0011731	0.00293	1.52	up	KIF14
TC1701435	0.0000117	0.0000628	1.52	up	KIF18B
TC17r00009	0.0002167	0.000672	1.52	up	KRTAP1-5
TC1800009	0.0005799	0.00157	1.52	up	NDC80
TC1901256	0.0000031	0.0000246	1.52	up	NOTCH3
TC0X00100	0.001355	0.00332	1.52	up	POLA1
TC1201663	0.0000074	0.000045	1.52	up	RILPL2
TC1601097	0.0001477	0.000489	1.52	up	SALL1
TC0102481	0.0004854	0.00135	1.52	up	SASS6
TC1100448	0.000031	0.00014	1.52	up	TMEM109
TC1100112	0.000031	0.00014	1.52	up	TRIM22
TC0601487	0.0000415	0.000174	1.51	up	BVES
TC1200639	0.0010101	0.00257	1.51	up	DRAM1
TC0X00757	0.000074	0.000272	1.51	up	HDHD1
TC0600189	0.0000132	0.0000692	1.51	up	HIST1H2AG
TC2000554	0.0000053	0.0000355	1.51	up	JAG1
TC0201241	0.0000032	0.0000253	1.51	up	Q8N369_HUMAN
TC0X00021	0.0002225	0.000688	1.51	up	STS
TC1500445	0.0000356	0.000155	1.51	up	THSD4
TC0700724	0.0007262	0.00192	1.51	up	TMEM140
TC0101018	0.0000774	0.000282	1.5	up	ADAMTSL4
TC0101512	0.0000602	0.000232	1.5	up	CENPF
TC0400122	0.0011874	0.00296	1.5	up	NCAPG
TC0101236	0.0004184	0.00119	1.5	up	NUF2
TC0200215	0.0000585	0.000227	1.5	up	PRKCE
TC0102253	0.0000379	0.000163	1.5	up	RAB3B
TC0601755	0.0001495	0.000494	1.5	up	RNASET2
TC0100343	0.0000036	0.0000273	1.5	up	SERINC2
TC1000863	0.0000255	0.000119	1.5	up	ZNF438
TC1400844	0.0002154	0.000669	1.49	up	CDKL1
TC0501006	0.0002959	0.000878	1.49	up	DEPDC1B
TC1600652	0.0000084	0.0000494	1.49	up	FOXC2
TC1201307	0.0009792	0.00249	1.49	up	PRIM1
TC0100937	0.0000049	0.0000338	1.49	up	RNF115

TC1601070	0.0003398	0.000988	1.49	up	SHCBP1
TC0300075	0.000037	0.00016	1.49	up	SLC6A6
TC1200652	0.0000462	0.000189	1.49	up	TXNRD1
TC0101436	0.0000101	0.0000563	1.48	up	ATP2B4
TC1900247	0.0000061	0.0000393	1.48	up	CD97
TC0102705	0.0000426	0.000178	1.48	up	HIST2H3D
TC0901219	0.0000658	0.000247	1.48	up	KLF4
TC1201419	0.0001325	0.000448	1.48	up	LIN7A
TC1200053	0.0000249	0.000117	1.48	up	NCAPD2
TC0800830	0.0000102	0.0000565	1.48	up	PBK
TC0100677	0.0000568	0.000222	1.48	up	PTGFR
TC0600462	0.008258	0.0167	1.48	up	RN7SK
TC0100399	0.0000111	0.0000602	1.48	up	ZC3H12A
TC1500160	0.0001938	0.000613	1.47	up	ARHGAP11B
TC0601549	0.0039871	0.00868	1.47	up	COL10A1
TC0400724	0.0001133	0.000388	1.47	up	CYP4V2
TC1901561	0.0000317	0.000142	1.47	up	ETHE1
TC0200725	0.000162	0.00053	1.47	up	GPR39
TC1400419	0.0001298	0.000439	1.47	up	GPR65
TC0200534	0.0002846	0.000849	1.47	up	IL1R1
TC1101993	0.0001826	0.000583	1.47	up	NCAPD3
TC0600952	0.0000049	0.0000338	1.47	up	NEDD9
TC0301497	0.0000341	0.00015	1.47	up	PARP9
TC0200199	0.0004439	0.00125	1.47	up	PKDCC
TC1300079	0.0004362	0.00123	1.47	up	RFC3
TC1701308	0.0003437	0.000998	1.47	up	TOP2A
TC1200016	0.0003594	0.00104	1.46	up	CACNA1C
TC1100557	0.000118	0.000402	1.46	up	CCDC85B
TC0901162	0.0000436	0.000181	1.46	up	CDC14B
TC0400630	0.0001675	0.000541	1.46	up	GUCY1B3
TC0601019	0.0084411	0.0171	1.46	up	HIST1H2BB
TC1000132	0.0002146	0.000667	1.46	up	MASTL
TC1501243	0.0002909	0.000865	1.46	up	PRC1
TC0101275	0.0000316	0.000142	1.46	up	PRRX1
TC1201130	0.0176563	0.033	1.46	up	Q9P1G1_HUMAN
TC2000361	0.0000452	0.000185	1.46	up	RNF114
TC0500179	0.0000569	0.000222	1.46	up	SNX18
TC0700319	0.0006184	0.00167	1.46	up	ZNF138
TC1600210	0.0000066	0.0000417	1.45	up	ABCC1
TC1001079	0.0001126	0.000386	1.45	up	CAMK2G
TC1000271	0.0023588	0.00541	1.45	up	CDK1
TC1000453	0.0000312	0.00014	1.45	up	CEP55
TC0501517	0.0000054	0.0000359	1.45	up	CLTB
TC0500059	0.0004797	0.00134	1.45	up	FBXL7

TC0600151	0.0000511	0.000204	1.45	up	HIST1H2BC
TC0201889	0.000015	0.0000763	1.45	up	HS6ST1
TC04r00004	0.0000066	0.0000417	1.45	up	LOC389834
TC0103208	0.0002757	0.000827	1.45	up	NEK2
TC1200032	0.0014	0.00342	1.45	up	RAD51AP1
TC0601698	0.0000787	0.000286	1.45	up	RGS17
TC1700017	0.0000092	0.0000526	1.45	up	SERPINF1
TC1800007	0.0000054	0.0000359	1.45	up	TYMS
TC1101010	0.0000869	0.000309	1.44	up	ADAMTS15
TC0500591	0.000067	0.000251	1.44	up	ADRB2
TC1500373	0.0002242	0.000692	1.44	up	CCNB2
TC0601006	0.0000657	0.000247	1.44	up	CMAH
TC0102349	0.0045472	0.00979	1.44	up	DEPDC1
TC0201643	0.00001	0.0000559	1.44	up	LOXL3
TC0401223	0.001542	0.00373	1.44	up	MAD2L1
TC1400756	0.0015495	0.00374	1.44	up	NOVA1
TC1000802	0.0004827	0.00134	1.44	up	PTPLA
TC1201246	0.000017	0.0000846	1.44	up	RARG
TC0200567	0.0000096	0.0000544	1.44	up	SH3RF3
TC0400123	0.000514	0.00141	1.44	up	SLIT2
TC1101228	0.0063392	0.0132	1.44	up	SNORD14A
TC1400754	0.0001001	0.000347	1.44	up	STXBP6
TC0202292	0.0018274	0.00431	1.43	up	BARD1
TC1001257	0.0002487	0.000757	1.43	up	CALHM2
TC1100272	0.0001458	0.000484	1.43	up	CAT
TC0800160	0.0003755	0.00108	1.43	up	CDCA2
TC1300347	0.0031336	0.00698	1.43	up	CENPJ
TC0Y00109	0.0091547	0.0184	1.43	up	CRLF2
TC0701733	0.0010959	0.00276	1.43	up	EZH2
TC0601628	0.0022324	0.00517	1.43	up	FAM54A
TC0200235	0.0001986	0.000626	1.43	up	FLJ46838
TC0900415	0.0002355	0.000721	1.43	up	HABP4
TC0500271	0.0003583	0.00104	1.43	up	HEXB
TC1000447	0.0011376	0.00286	1.43	up	KIF11
TC1200780	0.002848	0.00642	1.43	up	KNTC1
TC0101155	0.0000171	0.000085	1.43	up	PEAR1
TC1000968	0.0001896	0.000602	1.43	up	PGBD3
TC1200177	0.0001161	0.000396	1.43	up	PLEKHA5
TC1701048	0.0000086	0.00005	1.43	up	PMP22
TC2000524	0.000318	0.000937	1.43	up	Q5VXX5_HUMAN
TC0600842	0.0000106	0.0000581	1.43	up	ZDHHC14
TC1600613	0.0003174	0.000936	1.42	up	CENPN
TC0101498	0.0007775	0.00203	1.42	up	DTL
TC0601052	0.0011077	0.00279	1.42	up	HIST1H1B

TC0200073	0.0084084	0.017	1.42	up	KCNS3
TC1101279	0.0014982	0.00364	1.42	up	KIF18A
TC17r00008	0.0067056	0.0139	1.42	up	KRTAP1-3
TC1200536	0.0003595	0.00104	1.42	up	NAV3
TC0600481	0.0027737	0.00628	1.42	up	PRIM2
TC0901382	0.0000326	0.000145	1.42	up	PTGES
TC0701662	0.0000157	0.0000789	1.42	up	PTN
TC1500523	0.0064174	0.0134	1.42	up	Q9UD29_HUMAN
TC0102233	0.0002629	0.000795	1.42	up	STIL
TC1200615	0.0007383	0.00194	1.42	up	TMPO
TC0103333	0.0000977	0.00034	1.41	up	ABCB10
TC0700185	0.0001987	0.000626	1.41	up	ANLN
TC1500295	0.0001991	0.000626	1.41	up	B2M
TC1701398	0.002867	0.00645	1.41	up	BRCA1
TC0801128	0.0089905	0.0181	1.41	up	CCNE2
TC0100539	0.0003852	0.0011	1.41	up	CDKN2C
TC0102109	0.0016226	0.00389	1.41	up	CLSPN
TC0800526	0.0000276	0.000128	1.41	up	COL14A1
TC0101458	0.0012289	0.00305	1.41	up	FAM72A
TC0401107	0.0002872	0.000856	1.41	up	HSD17B11
TC1000426	0.0000537	0.000211	1.41	up	IFIT2
TC0X00291	0.0009577	0.00245	1.41	up	LOC550643
TC1700614	0.015676	0.0297	1.41	up	NOG
TC1300144	0.0009573	0.00245	1.41	up	PHF11
TC0700807	0.0000248	0.000117	1.41	up	PIP
TC0301486	0.0029545	0.00663	1.41	up	POLQ
TC0600596	0.0003913	0.00112	1.41	up	PRDM1
TC1400118	0.0000281	0.000129	1.41	up	PSME1
TC1400561	0.0146007	0.0279	1.41	up	Q6ZWF7_HUMAN
TC0101313	0.0006337	0.00171	1.41	up	RALGPS2
TC0102760	0.000024	0.000114	1.41	up	S100A10
TC2200189	0.0000333	0.000147	1.41	up	SEC14L2
TC1700308	0.0151566	0.0288	1.41	up	SNORD42A
TC0201821	0.0002351	0.000721	1.4	up	BUB1
TC0600982	0.0000991	0.000344	1.4	up	DEK
TC1000576	0.0000145	0.0000744	1.4	up	DUSP5
TC1700588	0.0000135	0.0000704	1.4	up	EME1
TC1700073	0.0000687	0.000255	1.4	up	FAM64A
TC1600455	0.0221781	0.0404	1.4	up	MT1M
TC1400295	0.0000444	0.000184	1.4	up	MTHFD1
TC0200033	0.000146	0.000484	1.4	up	RRM2
TC1300143	0.0035215	0.00773	1.4	up	SETDB2
TC1000560	0.0002657	0.000802	1.4	up	SLK
TC0500426	0.0070873	0.0146	1.4	up	ZNF474

TC1500996	0.0007769	0.00203	1.39		up	ADAM10
TC0201287	0.0001393	0.000467	1.39		up	CMPK2
TC1700875	0.0000429	0.000178	1.39		up	FAM101B
TC0600332	0.0000234	0.000111	1.39		up	FANCE
TC0X01255	0.0000765	0.00028	1.39		up	GPC3
TC0301205	0.0002233	0.00069	1.39		up	GPX1
TC0601029	0.0009283	0.00238	1.39		up	HIST1H3F
TC0400977	0.0000626	0.000238	1.39		up	IGFBP7
TC0500505	0.0003694	0.00106	1.39		up	KIF20A
TC0102743	0.0206422	0.0379	1.39		up	LOC100507670
TC0800408	0.0086379	0.0174	1.39		up	LRRCC1
TC0601631	0.0004527	0.00128	1.39		up	MAP3K5
TC0X01287	0.0000656	0.000247	1.39		up	MAP7D3
TC1001350	0.0000633	0.00024	1.39		up	MKI67
TC0200973	0.0025816	0.00589	1.39		up	SGOL2
TC0800399	0.0016621	0.00397	1.39		up	ZBTB10
TC0102899	0.0004639	0.0013	1.38		up	AIM2
TC1200213	0.0128392	0.025	1.38		up	ARNTL2
TC1400477	0.0001723	0.000554	1.38		up	BDKRB1
TC1300142	0.0012267	0.00304	1.38		up	CDADC1
TC2200512	0.0002784	0.000833	1.38		up	CHCHD10
TC1500370	0.012216	0.0238	1.38		up	FAM63B
TC1400709	0.000251	0.000763	1.38		up	HAUS4
TC0102704	0.0011797	0.00294	1.38		up	HIST2H2BF
TC1700483	0.0002091	0.000653	1.38		up	IFI35
TC1500437	0.0017357	0.00413	1.38		up	KIF23
TC0X00766	0.0000388	0.000166	1.38		up	MID1
TC1200641	0.0128688	0.025	1.38		up	PARPBP
TC0100058	0.0003337	0.000974	1.38		up	PRDM16
TC1900637	0.0002068	0.000647	1.38		up	Q6P662_HUMAN
TC2000721	0.0051032	0.0109	1.38		up	RBL1
TC0400052	0.0005397	0.00148	1.38		up	STK32B
TC1500572	0.000339	0.000987	1.38		up	TM6SF1
TC0500010	0.000169	0.000546	1.38		up	TRIP13
TC0X00069	0.0007004	0.00186	1.38		up	TXLNG
TC1600634	0.0001645	0.000535	1.38		up	WFDC1
TC0X01072	0.0043381	0.00936	1.37		up	hsa-mir-361
TC1401080	0.0000623	0.000238	1.37		up	LGMN
TC0500441	0.0012393	0.00307	1.37		up	LMNB1
TC0601328	0.000461	0.00129	1.37		up	MCM3
TC0X00742	0.0006096	0.00165	1.37		up	PRKX
TC0X00947	0.0004596	0.00129	1.37		up	SMC1A
TC1101493	0.0043024	0.0093	1.37		up	SNORD31
TC0900420	0.0016532	0.00396	1.37		up	TDRD7

TC1901188	0.0002182	0.000676	1.37	up	ZNF823
TC1101668	0.0002066	0.000647	1.36	up	ARRB1
TC0801228	0.0070914	0.0146	1.36	up	ATAD2
TC1600228	0.0012842	0.00317	1.36	up	CCP110
TC1700433	0.0026311	0.00599	1.36	up	CDC6
TC1200634	0.0006841	0.00182	1.36	up	CHPT1
TC0Xr00009	0.0035314	0.00774	1.36	up	CRLF2
TC0400313	0.0016923	0.00404	1.36	up	CXCL6
TC0202359	0.0002672	0.000806	1.36	up	DOCK10
TC2200616	0.0007993	0.00209	1.36	up	LARGE
TC0100712	0.000613	0.00165	1.36	up	LRRC8C
TC1201441	0.0005671	0.00154	1.36	up	LUM
TC2100034	0.0004218	0.0012	1.36	up	NCAM2
TC0701805	0.0055392	0.0117	1.36	up	NCAPG2
TC0200492	0.0002552	0.000773	1.36	up	NCAPH
TC0300983	0.0081201	0.0165	1.36	up	OXTR
TC0600297	0.0061576	0.0129	1.36	up	PSMB9
TC0103039	0.0222606	0.0405	1.36	up	Q6NWZ2_HUMAN
TC0201668	0.0005401	0.00148	1.36	up	Q86V40_HUMAN
TC1501054	0.0003366	0.00098	1.36	up	SNAPC5
TC1200989	0.0459835	0.0767	1.36	up	TAS2R42
TC1201314	0.0031825	0.00708	1.36	up	TMEM194A
TC0701590	0.0032073	0.00713	1.35	up	AASS
TC0700644	0.0069383	0.0143	1.35	up	C7orf58
TC0401233	0.0002095	0.000653	1.35	up	CCNA2
TC1200416	0.0004825	0.00134	1.35	up	CDK2
TC1500607	0.0027169	0.00617	1.35	up	FANCI
TC0100793	0.0056164	0.0119	1.35	up	GPSM2
TC0601024	0.0038031	0.0083	1.35	up	HIST1H3F
TC0600715	0.0017165	0.00409	1.35	up	LAMA2
TC0600259	0.0097521	0.0195	1.35	up	NM_181717.2
TC1201187	0.0002326	0.000714	1.35	up	RACGAP1
TC0300755	0.0025529	0.00583	1.35	up	SMC4
TC1700309	0.0316336	0.0554	1.35	up	SNORD4B
TC1600183	0.0000988	0.000343	1.35	up	SNX29
TC0201667	0.0003871	0.00111	1.35	up	SUCLG1
TC0600981	0.0079795	0.0162	1.35	up	TPMT
TC0700737	0.0012861	0.00317	1.35	up	TRIM24
TC1101651	0.000608	0.00164	1.35	up	UCP2
TC1700071	0.000161	0.000527	1.35	up	WSCD1
TC0800104	0.0011509	0.00288	1.35	up	ZDHHC2
TC0100735	0.0237765	0.0429	1.34	up	CCDC18
TC0601030	0.0009229	0.00237	1.34	up	HIST1H3F
TC0601055	0.0016138	0.00387	1.34	up	HIST1H3F

TC1101956	0.0002496	0.000759	1.34	up	KIRREL3
TC1800299	0.004907	0.0105	1.34	up	METTL4
TC0800527	0.0191411	0.0355	1.34	up	MTBP
TC0600538	0.0003204	0.000942	1.34	up	NT5E
TC0102257	0.0001434	0.000478	1.34	up	ORC1
TC1300660	0.000825	0.00214	1.34	up	PCID2
TC0300341	0.0001946	0.000615	1.34	up	PTPRG
TC0500663	0.0005473	0.00149	1.34	up	PTTG1
TC0301263	0.0015693	0.00378	1.34	up	SFMBT1
TC1101457	0.0009598	0.00245	1.34	up	SLC15A3
TC0101131	0.0000878	0.000312	1.34	up	SYT11
TC1900520	0.0011393	0.00286	1.34	up	ZFP36
TC0600480	0.0011424	0.00287	1.33	up	BAG2
TC0500232	0.0003296	0.000965	1.33	up	CCNB1
TC0300799	0.006006	0.0126	1.33	up	ECT2
TC1400770	0.0030941	0.0069	1.33	up	HEATR5A
TC1200040	0.0495194	0.082	1.33	up	KCNA1
TC0101057	0.002933	0.00659	1.33	up	LCE2D
TC1700881	0.0038912	0.00848	1.33	up	LOC100130876
TC1500546	0.0298671	0.0526	1.33	up	MESDC1
TC0301639	0.0007979	0.00208	1.33	up	PLSCR1
TC1400831	0.0172715	0.0323	1.33	up	POLE2
TC1901157	0.0123967	0.0242	1.33	up	S1PR2
TC0101488	0.0000903	0.000319	1.33	up	SERTAD4
TC1800181	0.0111482	0.022	1.33	up	SKA1
TC0401267	0.0063934	0.0133	1.33	up	SLC7A11
TC1101138	0.0002314	0.000711	1.33	up	TRIM5
TC2200235	0.0015697	0.00378	1.32	up	APOL6
TC0400585	0.0030629	0.00685	1.32	up	ARHGAP10
TC0X00360	0.0158811	0.03	1.32	up	BMP2KL
TC0600160	0.0005879	0.00159	1.32	up	BTN3A1
TC0800075	0.0183259	0.0342	1.32	up	C8orf49
TC0300461	0.0015146	0.00367	1.32	up	CEP97
TC0500714	0.0065825	0.0137	1.32	up	CREBRF
TC0700455	0.0069089	0.0143	1.32	up	DBF4
TC0400319	0.0170955	0.0321	1.32	up	EREG
TC0200233	0.0139402	0.0269	1.32	up	FOXN2
TC1701711	0.000827	0.00215	1.32	up	RNF157
TC0200244	0.0001908	0.000605	1.32	up	SPTBN1
TC1000423	0.000488	0.00135	1.32	up	STAMBPL1
TC1701737	0.0001511	0.000498	1.32	up	TK1
TC0500318	0.0004481	0.00126	1.32	up	VCAN
TC1101474	0.0002268	0.000699	1.31	up	AHNAK
TC1701373	0.0001435	0.000478	1.31	up	DHX58

TC0102836	0.0007365	0.00194	1.31	up	ERVK-7
TC1400863	0.0054663	0.0116	1.31	up	GNPNAT1
TC0100898	0.0074605	0.0153	1.31	up	HIST2H3D
TC1200598	0.0477276	0.0794	1.31	up	hsa-mir-331
TC0401086	0.0048572	0.0104	1.31	up	LIN54
TC0501098	0.0030086	0.00673	1.31	up	MTX3
TC0301070	0.0050289	0.0107	1.31	up	NEK10
TC1400529	0.0051469	0.011	1.31	up	NR_003197.1
TC0103286	0.0019247	0.00452	1.31	up	PARP1
TC1800327	0.0005258	0.00144	1.31	up	PPP4R1
TC1400910	0.0049517	0.0106	1.31	up	SGPP1
TC0401321	0.0011409	0.00286	1.31	up	SH3D19
TC1000264	0.0350967	0.0607	1.31	up	TFAM
TC0100653	0.0005321	0.00146	1.31	up	TNNI3K
TC1500286	0.0018535	0.00437	1.31	up	WDR76
TC1901357	0.0116257	0.0228	1.31	up	ZNF429
TC0201953	0.002392	0.00548	1.31	up	ZRANB3
TC1800378	0.000255	0.000773	1.3	up	ANKRD29
TC0701537	0.0002359	0.000722	1.3	up	ATXN7L1
TC0600158	0.0017524	0.00416	1.3	up	BTN3A2
TC0X00808	0.0062603	0.0131	1.3	up	EIF1AX
TC0601053	0.0008611	0.00223	1.3	up	HIST1H3F
TC0601016	0.0163779	0.0309	1.3	up	HIST1H4B
TC0301646	0.0078834	0.0161	1.3	up	HLTF
TC0X00328	0.0027293	0.00619	1.3	up	KIF4B
TC1200250	0.0052417	0.0111	1.3	up	LRRK2
TC1300424	0.0006257	0.00169	1.3	up	MRPS31
TC1101179	0.0034106	0.00751	1.3	up	NLRP10
TC0301045	0.0003854	0.0011	1.3	up	RFTN1
TC0102788	0.0048551	0.0104	1.3	up	S100A3
TC1701691	0.0004819	0.00134	1.3	up	SLC25A19
TC0103012	0.0024386	0.00558	1.3	up	SNORD74
TC1701170	0.0009298	0.00238	1.3	up	SPAG5
TC0800543	0.0037946	0.00829	1.3	up	TRMT12
TC0200177	0.000565	0.00154	1.3	up	VIT
TC0500391	0.0069832	0.0144	1.29	up	CAMK4
TC0800828	0.0010017	0.00255	1.29	up	CCDC25
TC0301174	0.0091326	0.0184	1.29	up	CDC25A
TC1300426	0.0012779	0.00316	1.29	up	ELF1
TC1901747	0.0364051	0.0627	1.29	up	FLJ30403
TC1701330	0.0003998	0.00114	1.29	up	KRTAP1-3
TC0301757	0.0021909	0.00509	1.29	up	NCEH1
TC0200584	0.0027523	0.00623	1.29	up	POLR1B
TC0301404	0.0498442	0.0824	1.29	up	SENP7

TC0700500	0.037042	0.0635	1.28	up	ACN9
TC0601657	0.0017108	0.00408	1.28	up	ADAT2
TC1901237	0.0036958	0.00809	1.28	up	ASF1B
TC2000849	0.0022284	0.00516	1.28	up	AURKA
TC0300441	0.002597	0.00592	1.28	up	C3orf26
TC0400714	0.0293294	0.0517	1.28	up	CCDC111
TC0102974	0.0015598	0.00376	1.28	up	DPT
TC0100788	0.0303435	0.0533	1.28	up	FAM102B
TC0600157	0.0040402	0.00879	1.28	up	HIST1H2BO
TC1500169	0.0032302	0.00716	1.28	up	KLF13
TC0400845	0.005722	0.0121	1.28	up	LCORL
TC0800697	0.0178834	0.0334	1.28	up	LOC728731
TC1201413	0.0027533	0.00623	1.28	up	PAWR
TC2000533	0.0022239	0.00516	1.28	up	PCNA
TC0300490	0.0014602	0.00356	1.28	up	PVRL3
TC0900171	0.0012076	0.003	1.28	up	RECK
TC0100775	0.0101784	0.0203	1.28	up	S1PR1
TC0100912	0.0013973	0.00341	1.28	up	SRGAP2P2
TC0100655	0.0020751	0.00484	1.28	up	TYW3
TC0401197	0.0453476	0.0758	1.27	up	C4orf21
TC0401163	0.0131433	0.0255	1.27	up	CENPE
TC0103011	0.0364058	0.0627	1.27	up	CENPL
TC1600924	0.0251799	0.0451	1.27	up	ERI2
TC0501440	0.002831	0.00638	1.27	up	GEMIN5
TC0601472	0.0187243	0.0348	1.27	up	MMS22L
TC1300101	0.021856	0.0398	1.27	up	NAA16
TC1400553	0.0119889	0.0234	1.27	up	NR_003217.1
TC1400976	0.0009079	0.00233	1.27	up	NUMB
TC1200482	0.0116132	0.0228	1.27	up	RASSF3
TC0500425	0.008642	0.0174	1.27	up	SRFBP1
TC1400890	0.0222332	0.0404	1.27	up	TIMM9
TC0102998	0.0053602	0.0114	1.27	up	VAMP4
TC1101665	0.0033152	0.00732	1.27	up	XRRA1
TC1500798	0.007001	0.0144	1.26	up	FMN1
TC0500673	0.0236522	0.0428	1.26	up	HMMR
TC0X00282	0.0116816	0.0229	1.26	up	MAGEH1
TC0900176	0.0140195	0.027	1.26	up	MELK
TC0700597	0.0030847	0.00688	1.26	up	MLL5
TC0800736	0.0054042	0.0114	1.26	up	PINX1
TC1000654	0.0204874	0.0377	1.26	up	Q499Y3_HUMAN
TC1200341	0.0026559	0.00604	1.26	up	SLC4A8
TC1000555	0.0139993	0.027	1.26	up	TAF5
TC1100829	0.0143442	0.0275	1.26	up	ZC3H12C
TC0X00423	0.02607	0.0466	1.25	up	CENPI

TC0600894	0.006484	0.0135	1.25	up	EXOC2
TC0300039	0.0139208	0.0269	1.25	up	FANCD2
TC0200071	0.0193468	0.0358	1.25	up	GEN1
TC0X00121	0.003421	0.00752	1.25	up	GK
TC0401196	0.0096259	0.0193	1.25	up	LOC91431
TC0501442	0.0242936	0.0437	1.25	up	NP10_HUMAN
TC0900789	0.0063706	0.0133	1.25	up	PSIP1
TC0601351	0.0041934	0.00908	1.25	up	RAB23
TC1800284	0.0056423	0.0119	1.25	up	RBFA
TC2200196	0.0051581	0.011	1.25	up	SLC35E4
TC0700759	0.0448764	0.0751	1.25	up	TAS2R4
TC2000559	0.0211331	0.0387	1.24	up	C20orf61
TC0901299	0.0145588	0.0278	1.24	up	C5
TC0200213	0.0276566	0.0492	1.24	up	CAMKMT
TC0301570	0.0046076	0.00991	1.24	up	CPNE4
TC1001219	0.0113309	0.0223	1.24	up	CWF19L1
TC1400874	0.0107944	0.0214	1.24	up	DLGAP5
TC0800248	0.039416	0.0671	1.24	up	GINS4
TC1100770	0.0195666	0.0362	1.24	up	KDM4D
TC0500253	0.0323029	0.0564	1.24	up	MCCC2
TC0800720	0.0400393	0.068	1.24	up	MFHAS1
TC1900975	0.0148702	0.0283	1.24	up	MIER2
TC1500773	0.0245817	0.0442	1.24	up	Q6ZSR3_HUMAN
TC0202078	0.0407669	0.0692	1.24	up	SPC25
TC1000826	0.0066551	0.0138	1.23	up	ARHGAP21
TC0801173	0.0126059	0.0245	1.23	up	AZIN1
TC0501322	0.0227321	0.0412	1.23	up	CDC25C
TC1200266	0.0065527	0.0136	1.23	up	IRAK4
TC1400266	0.0133072	0.0258	1.23	up	KIAA0586
TC17r00007	0.0130256	0.0253	1.23	up	KRTAP1-1
TC0400171	0.0174366	0.0326	1.23	up	LIAS
TC0100673	0.0042205	0.00913	1.23	up	NEXN
TC0801196	0.0370025	0.0635	1.23	up	NUDCD1
TC0100665	0.0191573	0.0355	1.23	up	ST6GALNAC5
TC1500421	0.0141778	0.0273	1.23	up	ZWILCH
TC1701199	0.0049133	0.0105	1.22	up	BLMH
TC0800053	0.0320171	0.056	1.22	up	ERI1
TC1900846	0.0349906	0.0606	1.22	up	hsa-mir-371
TC2000060	0.0166935	0.0314	1.22	up	MCM8
TC0200228	0.049067	0.0813	1.22	up	MSH2
TC1100664	0.0069969	0.0144	1.22	up	POLD3
TC2000734	0.0187632	0.0349	1.22	up	SNORA71A
TC1700397	0.0093668	0.0188	1.22	up	TADA2A
TC0601087	0.0479317	0.0797	1.21	up	HCG4

TC0X00168	0.0367424	0.0632	1.21	up	KDM6A
TC0900528	0.0105858	0.021	1.21	up	MRRF
TC1400103	0.031174	0.0547	1.21	up	NGDN
TC1200537	0.0105859	0.021	1.21	up	SYT1
TC0102446	0.0153143	0.029	1.21	up	TMED5
TC1900601	0.033431	0.0582	1.21	up	ZNF284
TC0400343	0.0203805	0.0375	1.2	up	BMP2K
TC0701122	0.0263038	0.0469	1.2	up	DPY19L1
TC0200802	0.0102548	0.0205	1.2	up	GPD2
TC0700710	0.0288022	0.0509	1.2	up	hsa-mir-335
TC0103133	0.0111861	0.022	1.2	up	KLHL12
TC0102351	0.0268228	0.0478	1.2	up	LRRC40
TC1101773	0.0182075	0.034	1.2	up	MMP8
TC2000671	0.0231677	0.042	1.2	up	PXMP4
TC2000306	0.0236774	0.0428	1.2	up	STK4
TC0200996	0.0116063	0.0228	1.2	up	STRADB
TC0400497	0.0172415	0.0323	1.2	up	USP53
TC0103217	0.0412055	0.0699	1.19	up	BATF3
TC2100076	0.0121438	0.0237	1.19	up	FAM176C
TC0200860	0.042106	0.0712	1.19	up	HAT1
TC0800816	0.0306089	0.0538	1.19	up	KCTD9
TC0100257	0.037821	0.0647	1.19	up	PITHD1
TC0202405	0.0377661	0.0647	1.18	up	DNAJB3
TC0301560	0.0363855	0.0627	1.18	up	PIK3R4
TC0X00590	0.0139676	0.0269	1.18	up	SLC9A6
TC0901094	0.0286086	0.0506	1.18	up	ZCCHC6
TC1201648	0.0442831	0.0743	1.18	up	ZCCHC8
TC0200200	0.0457928	0.0764	1.17	up	EML4
TC1200158	0.0427668	0.0721	1.16	up	EMP1
TC1401095	0.0431171	0.0726	0.86	down	SERPINA1
TC0101814	0.0327383	0.0572	0.85	down	PLEKHG5
TC1101205	0.045273	0.0757	0.84	down	MRVI1
TC0202043	0.0317314	0.0555	0.83	down	RBMS1
TC1100940	0.015758	0.0298	0.82	down	C11orf63
TC0701147	0.0473629	0.0788	0.82	down	FAM183B
TC1600119	0.0189381	0.0352	0.81	down	FLJ39639
TC0401460	0.0279823	0.0496	0.8	down	CCDC110
TC0800409	0.0107673	0.0213	0.8	down	E2F5
TC0200023	0.0038719	0.00844	0.8	down	ID2
TC0301465	0.0204696	0.0377	0.8	down	LSAMP
TC0300725	0.015081	0.0286	0.8	down	RAP2B
TC0700666	0.0296581	0.0522	0.8	down	SND1-IT1
TC0300420	0.0046992	0.0101	0.79	down	ARL6
TC0103140	0.0108289	0.0214	0.79	down	CHI3L1

TC1100678	0.0087947	0.0177	0.79	down	DGAT2
TC0103220	0.0019325	0.00453	0.79	down	FLVCR1-AS1
TC0600154	0.0034153	0.00751	0.79	down	HIST1H3F
TC2200090	0.0339412	0.0591	0.79	down	IGLV11-55
TC1900974	0.0023402	0.00538	0.79	down	PPAP2C
TC0601269	0.0034069	0.0075	0.79	down	PRPH2
TC1700564	0.0417751	0.0707	0.79	down	Q96FJ8_HUMAN
TC1000751	0.0112937	0.0222	0.79	down	SFMBT2
TC1901846	0.0128118	0.0249	0.79	down	ZNF835
TC1400748	0.0350746	0.0607	0.78	down	CBLN3
TC2100251	0.0116627	0.0228	0.78	down	CYYR1
TC1900541	0.0135449	0.0262	0.78	down	ITPKC
TC0X00908	0.001986	0.00465	0.78	down	KCND1
TC0102079	0.0167139	0.0314	0.78	down	MARCKSL1
TC1200462	0.0079037	0.0161	0.78	down	METTL21B
TC0800004	0.0279597	0.0496	0.78	down	Q8NB26_HUMAN
TC1500606	0.0030664	0.00685	0.77	down	ABHD2
TC0500954	0.0081884	0.0166	0.77	down	ANXA2R
TC0400249	0.0109378	0.0216	0.77	down	ARL9
TC1501024	0.0111719	0.022	0.77	down	DAPK2
TC1701483	0.0075904	0.0155	0.77	down	HOXB6
TC0301308	0.0018524	0.00437	0.77	down	ID2B
TC0400520	0.0012008	0.00299	0.77	down	INTU
TC0600756	0.0017554	0.00416	0.77	down	KIAA1244
TC0102756	0.0280248	0.0497	0.77	down	LOC100132111
TC2200046	0.0018043	0.00427	0.77	down	LOC729444
TC0201021	0.0142909	0.0274	0.77	down	NBEAL1
TC0900558	0.0097982	0.0196	0.77	down	OLFML2A
TC1700857	0.0044333	0.00956	0.77	down	Q6ZNS0_HUMAN
TC0700003	0.0293603	0.0517	0.77	down	Q8N814_HUMAN
TC1300653	0.007606	0.0155	0.77	down	RAB20
TC0700029	0.0010718	0.0027	0.77	down	SDK1
TC0Y00002	0.0075546	0.0154	0.77	down	SHOX
TC1500356	0.0030655	0.00685	0.77	down	TEX9
TC1400355	0.0083767	0.017	0.76	down	ACOT4
TC1700115	0.0080136	0.0163	0.76	down	ALOX15B
TC1601311	0.0183724	0.0342	0.76	down	C16orf74
TC0300407	0.0089021	0.0179	0.76	down	EPHA3
TC1700642	0.0003946	0.00112	0.76	down	GDPD1
TC0900205	0.0378038	0.0647	0.76	down	LOC728903
TC0501317	0.0062593	0.0131	0.76	down	NME5
TC1101116	0.039174	0.0667	0.76	down	OR51G2
TC2000021	0.0067152	0.0139	0.76	down	SIRPA
TC0501407	0.0133457	0.0258	0.75	down	ARSI

TC0201018	0.0004325	0.00123	0.75	down	FAM117B
TC0300743	0.0021718	0.00505	0.75	down	NM_001099777.1
TC1400532	0.0061733	0.0129	0.75	down	NR_003199.1
TC2100357	0.0153061	0.029	0.75	down	PLAC4
TC0401130	0.0016518	0.00395	0.75	down	UNC5C
TC0202354	0.0005016	0.00139	0.74	down	AP1S3
TC1101815	0.00066	0.00177	0.74	down	ARHGAP20
TC1000793	0.0070947	0.0146	0.74	down	C1QL3
TC1500247	0.001491	0.00362	0.74	down	CHAC1
TC1200961	0.0003442	0.000999	0.74	down	CLEC2B
TC0300421	0.0012024	0.00299	0.74	down	CRYBG3
TC0900989	0.0354718	0.0613	0.74	down	FAM27A
TC1100997	0.0001486	0.000491	0.74	down	FLI1
TC0700670	0.0004734	0.00132	0.74	down	HILPDA
TC0601022	0.0227302	0.0412	0.74	down	HIST1H2BC
TC0201818	0.0000956	0.000334	0.74	down	LIMS3
TC2100173	0.0152341	0.0289	0.74	down	LRRC3
TC2000244	0.0022777	0.00526	0.74	down	NNAT
TC1900512	0.0007685	0.00201	0.74	down	PAPL
TC0202122	0.0023434	0.00538	0.74	down	Q8NAT4_HUMAN
TC0101376	0.0032945	0.00728	0.74	down	RGS2
TC1700074	0.0027456	0.00622	0.74	down	TXNDC17
TC2200491	0.0002804	0.000839	0.74	down	ZNF280B
TC0200833	0.0000278	0.000128	0.73	down	B3GALT1
TC0800487	0.0003309	0.000967	0.73	down	BAALC
TC0X01111	0.0000618	0.000236	0.73	down	BEX1
TC1400363	0.0003182	0.000937	0.73	down	C14orf45
TC0201673	0.0003832	0.0011	0.73	down	CAPG
TC1200471	0.0076618	0.0156	0.73	down	hsa-let-7i
TC0X00760	0.0005051	0.00139	0.73	down	KAL1
TC0500570	0.0012229	0.00304	0.73	down	KCTD16
TC1101342	0.0001333	0.00045	0.73	down	LRP4
TC0201340	0.0000501	0.0002	0.73	down	MATN3
TC0201158	0.0002931	0.000871	0.73	down	SP140
TC2000421	0.0047569	0.0102	0.72	down	C20orf197
TC0900232	0.0039872	0.00868	0.72	down	FAM27A
TC1001152	0.0000387	0.000166	0.72	down	HTR7
TC1201403	0.0002582	0.000781	0.72	down	PHLDA1
TC2100412	0.0000632	0.00024	0.72	down	POFUT2
TC2000369	0.0000514	0.000204	0.72	down	PTPN1
TC0X00070	0.0001822	0.000582	0.72	down	REPS2
TC0400717	0.000208	0.00065	0.72	down	SLC25A4
TC0100132	0.0002117	0.000658	0.72	down	TNFRSF1B
TC0101229	0.0001216	0.000413	0.72	down	UAP1

TC1900779	0.0007427	0.00195	0.72	down	ZNF528
TC2100138	0.0000332	0.000147	0.71	down	BACE2
TC1200190	0.0001578	0.000519	0.71	down	C12orf39
TC0300687	0.0008959	0.00231	0.71	down	C3orf58
TC1701061	0.0011568	0.0029	0.71	down	CENPV
TC1200079	0.0000415	0.000174	0.71	down	CLSTN3
TC0M00015	0.0145217	0.0278	0.71	down	COX3
TC2100109	0.0005228	0.00144	0.71	down	DOPEY2
TC0500284	0.0093788	0.0188	0.71	down	F2RL1
TC2000543	0.0000806	0.000292	0.71	down	FERMT1
TC0400931	0.0003648	0.00105	0.71	down	FRYL
TC2100220	0.0002678	0.000807	0.71	down	HSPA13
TC1501237	0.0000579	0.000225	0.71	down	IDH2
TC2100089	0.0000292	0.000133	0.71	down	ITSN1
TC1500395	0.0006343	0.00171	0.71	down	LACTB
TC1300215	0.0010273	0.0026	0.71	down	NDFIP2
TC0102557	0.0063033	0.0132	0.71	down	PHTF1
TC0X00492	0.0029906	0.0067	0.71	down	Q9P1I9_HUMAN
TC1501011	0.000045	0.000185	0.71	down	RORA
TC0800169	0.0000135	0.0000704	0.71	down	SCARA3
TC0301086	0.0000105	0.0000577	0.71	down	SUSD5
TC0300707	0.0013688	0.00335	0.71	down	TSC22D2
TC1700587	0.0000492	0.000197	0.71	down	XYLT2
TC0801191	0.000159	0.000522	0.7	down	ANGPT1
TC1300028	0.0002892	0.000861	0.7	down	C1QTNF9B-AS1
TC0X00227	0.0006803	0.00181	0.7	down	CLCN5
TC0300014	0.000474	0.00132	0.7	down	EDEM1
TC0800401	0.0075402	0.0154	0.7	down	FABP5
TC0900226	0.0000353	0.000154	0.7	down	FAM27A
TC0300896	0.0003274	0.000959	0.7	down	IL1RAP
TC0201721	0.0000392	0.000167	0.7	down	LOC654342
TC1000679	0.0040547	0.00881	0.7	down	LOC728158
TC0600340	0.0000356	0.000155	0.7	down	MAPK13
TC1600245	0.0001058	0.000366	0.7	down	METTL9
TC0102357	0.0001627	0.000531	0.7	down	NEGR1
TC1001060	0.0000913	0.000322	0.7	down	P4HA1
TC0400671	0.0000209	0.000101	0.7	down	PALLD
TC0200864	0.0000947	0.000332	0.7	down	PDK1
TC0Y00024	0.0004689	0.00131	0.7	down	PRKY
TC1000891	0.0140422	0.027	0.7	down	Q5SYT8_HUMAN
TC1400105	0.0421516	0.0712	0.7	down	Q6ZVK3_HUMAN
TC2100323	0.0000656	0.000247	0.7	down	RCAN1
TC0900364	0.0000508	0.000203	0.7	down	S1PR3
TC0301744	0.0001932	0.000612	0.7	down	SLC7A14

TC1300402	0.0000288	0.000132	0.7	down	SMAD9
TC0300631	0.0007923	0.00207	0.7	down	TMEM108
TC0401377	0.0000643	0.000243	0.7	down	TRIM61
TC0900485	0.0001273	0.000432	0.7	down	UGCG
TC1701121	0.0008087	0.00211	0.7	down	ULK2
TC0501025	0.0008688	0.00225	0.69	down	ADAMTS6
TC0500905	0.0000155	0.0000782	0.69	down	C1QTNF3
TC0801132	0.0022931	0.00529	0.69	down	C8orf37
TC0500690	0.0000097	0.0000548	0.69	down	CCDC99
TC0100720	0.0000414	0.000174	0.69	down	EPHX4
TC0101361	0.0000606	0.000233	0.69	down	HMCN1
TC1600930	0.0000829	0.000298	0.69	down	IGSF6
TC1600439	0.0012984	0.0032	0.69	down	IRX5
TC1001297	0.0007098	0.00188	0.69	down	KIAA1598
TC0200531	0.0000579	0.000225	0.69	down	MAP4K4
TC1400089	0.0000085	0.0000497	0.69	down	MMP14
TC1700621	0.0000387	0.000166	0.69	down	MSI2
TC0100760	0.0005039	0.00139	0.69	down	PALMD
TC0100893	0.0000072	0.0000442	0.69	down	PHGDH
TC0X01025	0.0002202	0.000682	0.69	down	PHKA1P1
TC0800777	0.0001633	0.000532	0.69	down	PSD3
TC0601453	0.0006388	0.00172	0.69	down	RRAGD
TC1600003	0.0000616	0.000236	0.69	down	SNRNP25
TC0400884	0.0053804	0.0114	0.69	down	TLR6
TC0801235	0.0017356	0.00413	0.69	down	TMEM65
TC0801204	0.0000614	0.000235	0.69	down	TRPS1
TC0X00955	0.0000655	0.000247	0.69	down	WNK3
TC2100128	0.0000405	0.00017	0.69	down	WRB
TC1700589	0.0000655	0.000247	0.68	down	ACSF2
TC0401025	0.0000829	0.000298	0.68	down	ADAMTS3
TC0700841	0.0000234	0.000111	0.68	down	ARHGEF5
TC2000834	0.0000909	0.000321	0.68	down	ATP9A
TC1100264	0.0000038	0.0000283	0.68	down	C11orf41
TC2100363	0.0000065	0.0000414	0.68	down	C2CD2
TC1800250	0.0007334	0.00193	0.68	down	CCDC102B
TC1100299	0.0000043	0.0000308	0.68	down	CD82
TC1101950	0.0000541	0.000212	0.68	down	CDON
TC0103405	0.0000676	0.000252	0.68	down	CHML
TC0401470	0.000047	0.000191	0.68	down	FAT1
TC0300334	0.0000696	0.000258	0.68	down	FLNB
TC1000777	0.0000739	0.000272	0.68	down	FRMD4A
TC0601160	0.0004668	0.00131	0.68	down	HB25_HUMAN
TC1001018	0.0006462	0.00173	0.68	down	HERC4
TC0601655	0.0002465	0.000751	0.68	down	HIVEP2

TC0200745	0.0003501	0.00101	0.68	down	HNMT
TC1701480	0.0023016	0.0053	0.68	down	HOXB4
TC0200570	0.0000619	0.000236	0.68	down	LIMS3
TC0601359	0.0416532	0.0705	0.68	down	LOC727945
TC1201526	0.0000452	0.000185	0.68	down	NUAK1
TC0600499	0.0001623	0.00053	0.68	down	OGFRL1
TC0301181	0.0000042	0.0000304	0.68	down	PFKFB4
TC1100325	0.000334	0.000974	0.68	down	PTPRJ
TC0800049	0.0000952	0.000333	0.68	down	Q9HBS9_HUMAN
TC1001169	0.0015648	0.00377	0.68	down	RBP4
TC0600428	0.0000097	0.0000548	0.68	down	RUNX2
TC1300354	0.0021192	0.00493	0.68	down	SHISA2
TC0X00881	0.0000341	0.00015	0.68	down	SLC9A7
TC2000075	0.000045	0.000185	0.68	down	SNAP25
TC0301697	0.0002829	0.000845	0.68	down	VEPH1
TC0201150	0.0000974	0.000339	0.68	down	WDR69
TC1900488	0.0000613	0.000235	0.68	down	ZNF793
TC1200702	0.0000182	0.0000894	0.67	down	ALDH2
TC0500858	0.0000319	0.000142	0.67	down	ANKH
TC0201582	0.0041786	0.00906	0.67	down	C1D
TC2100262	0.0001964	0.00062	0.67	down	CCT8
TC0201437	0.0000066	0.0000417	0.67	down	CDC42EP3
TC0601648	0.0000067	0.000042	0.67	down	CITED2
TC0300551	0.000443	0.00125	0.67	down	FAM162A
TC0901090	0.0000137	0.000071	0.67	down	GOLM1
TC1300121	0.0005778	0.00156	0.67	down	GTF2F2
TC1201652	0.0009695	0.00247	0.67	down	HCAR1
TC0200885	0.0000352	0.000154	0.67	down	HOXD4
TC0101685	0.0009274	0.00238	0.67	down	KIF26B
TC1400193	0.0001141	0.000391	0.67	down	LRFN5
TC1101672	0.000891	0.0023	0.67	down	MAP6
TC0301258	0.0000065	0.0000414	0.67	down	NT5DC2
TC0800928	0.0011734	0.00293	0.67	down	PCMTD1
TC1800446	0.0035708	0.00782	0.67	down	PIAS2
TC0700132	0.0059543	0.0125	0.67	down	Q96MZ3_HUMAN
TC1001222	0.001299	0.0032	0.67	down	Q9H354_HUMAN
TC0100916	0.0017448	0.00414	0.67	down	SCAF11
TC1201522	0.0001394	0.000467	0.67	down	SLC41A2
TC2100019	0.000294	0.000873	0.67	down	USP25
TC0400202	0.0001452	0.000483	0.66	down	ATP10D
TC1100790	0.0000993	0.000345	0.66	down	C11orf70
TC0401449	0.0000284	0.00013	0.66	down	CASP3
TC0Y00162	0.0017432	0.00414	0.66	down	CD24
TC0201025	0.0032139	0.00713	0.66	down	CYP20A1

TC1200881	0.0000519	0.000206	0.66	down	EFCAB4B
TC0500308	0.0003659	0.00105	0.66	down	FAM151B
TC0X00089	0.000517	0.00142	0.66	down	FAM3C
TC1100766	0.0000183	0.0000897	0.66	down	FUT4
TC1101722	0.0000217	0.000104	0.66	down	FZD4
TC2100046	0.0013501	0.00331	0.66	down	GABPA
TC1300297	0.0000085	0.0000497	0.66	down	GAS6
TC0200656	0.0000025	0.0000212	0.66	down	GYPC
TC0601169	0.0003024	0.000895	0.66	down	HLA-DOA
TC0200908	0.0001568	0.000516	0.66	down	ITGA4
TC1300547	0.0008694	0.00225	0.66	down	KLF12
TC1300341	0.0019278	0.00452	0.66	down	MIPEP
TC0301382	0.0009634	0.00246	0.66	down	PROS1
TC2100346	0.0001668	0.00054	0.66	down	PSMG1
TC0100338	0.0000451	0.000185	0.66	down	PTPRU
TC2100167	0.000009	0.0000516	0.66	down	PWP2
TC0701212	0.0000825	0.000298	0.66	down	SEC61G
TC0500951	0.0012858	0.00317	0.66	down	SEPP1
TC2000082	0.0000944	0.000332	0.66	down	SPTLC3
TC0900077	0.0000149	0.0000759	0.65	down	ADAMTSL1
TC0700470	0.0010202	0.00259	0.65	down	AKAP9
TC1700548	0.0000512	0.000204	0.65	down	C17orf57
TC1000052	0.0000041	0.0000298	0.65	down	CELF2
TC1800137	0.0000464	0.000189	0.65	down	DTNA
TC0901346	0.0000024	0.0000204	0.65	down	ENG
TC1800313	0.0000348	0.000153	0.65	down	EPB41L3
TC0701202	0.000008	0.0000475	0.65	down	GRB10
TC2100332	0.0000609	0.000234	0.65	down	HLCS
TC1500643	0.0095094	0.0191	0.65	down	HMGN1P38
TC2100086	0.0003941	0.00112	0.65	down	IFNAR1
TC0201297	0.0005036	0.00139	0.65	down	MBOAT2
TC1100864	0.0000068	0.0000425	0.65	down	NNMT
TC0301787	0.0000673	0.000252	0.65	down	PEX5L
TC1701803	0.0000532	0.00021	0.65	down	SECTM1
TC0401154	0.000139	0.000466	0.65	down	SLC39A8
TC1100953	0.0001878	0.000598	0.65	down	VWA5A
TC0600181	0.0001621	0.00053	0.65	down	ZNF391
TC1900914	0.0010211	0.00259	0.65	down	ZNF470
TC1001154	0.0000232	0.00011	0.64	down	ANKRD1
TC0400368	0.0000191	0.000093	0.64	down	ARHGAP24
TC0201171	0.0000034	0.0000265	0.64	down	ARMC9
TC0900793	0.0001351	0.000455	0.64	down	BNC2
TC0101291	0.00068	0.00181	0.64	down	C1orf9
TC2100379	0.0000032	0.0000253	0.64	down	CBS

TC1000694	0.0000356	0.000155	0.64	down	DPYSL4
TC0501456	0.0000146	0.0000748	0.64	down	EBF1
TC0501157	0.0000882	0.000313	0.64	down	ELL2
TC0700695	0.0000748	0.000275	0.64	down	FAM40B
TC1200151	0.000068	0.000253	0.64	down	GPRC5A
TC1501058	0.0000247	0.000117	0.64	down	LCTL
TC0100980	0.0001599	0.000525	0.64	down	LOC654342
TC1101778	0.0000056	0.000037	0.64	down	MMP13
TC2100205	0.000003	0.0000242	0.64	down	PRMT2
TC0900314	0.0000394	0.000167	0.64	down	PSAT1
TC1100589	0.0000035	0.0000268	0.64	down	RHOD
TC0200020	0.0000021	0.0000188	0.64	down	RNF144A
TC0500604	0.0000166	0.000083	0.64	down	SLC26A2
TC0300203	0.0000144	0.0000742	0.64	down	SNRK
TC0X00453	0.0000602	0.000232	0.64	down	TCEAL7
TC0900430	0.000031	0.00014	0.64	down	TGFBR1
TC0600648	0.0000022	0.0000192	0.64	down	WISP3
TC0700240	0.0000112	0.0000604	0.63	down	ABCA13
TC0500999	0.0000023	0.0000199	0.63	down	ACTBL2
TC0900080	0.0000827	0.000298	0.63	down	ADAMTSL1
TC2100188	0.0000021	0.0000188	0.63	down	ADARB1
TC0501233	0.000071	0.000263	0.63	down	DTWD2
TC0901075	0.0000077	0.0000463	0.63	down	FRMD3
TC0300089	0.0000023	0.0000199	0.63	down	GALNTL2
TC0102708	0.0009856	0.00251	0.63	down	HIST3H2BB
TC0601107	0.0000035	0.0000268	0.63	down	IER3
TC0101349	0.0000042	0.0000304	0.63	down	LAMC2
TC0102627	0.0000134	0.0000701	0.63	down	LOC728920
TC0301908	0.0000058	0.0000378	0.63	down	MFI2
TC1101874	0.0001065	0.000367	0.63	down	MPZL3
TC0700940	0.000018	0.0000888	0.63	down	PDGFA
TC0X00093	0.0010632	0.00268	0.63	down	SAT1
TC0501182	0.0000107	0.0000585	0.63	down	ST8SIA4
TC0202182	0.0000136	0.0000706	0.63	down	STAT4
TC0101487	0.0000123	0.0000657	0.63	down	SYT14
TC2000729	0.0000016	0.0000155	0.63	down	TGM2
TC0301650	0.000006	0.0000387	0.63	down	TM4SF18
TC0102492	0.0002076	0.000649	0.62	down	COL11A1
TC0501065	0.0000014	0.0000143	0.62	down	ENC1
TC1800141	0.000687	0.00183	0.62	down	GALNT1
TC0400851	0.0001416	0.000473	0.62	down	GPR125
TC1700648	0.0007399	0.00194	0.62	down	hsa-mir-21
TC1501260	0.0019993	0.00467	0.62	down	LOC644192
TC2100259	0.0005699	0.00155	0.62	down	LTN1

TC1601292	0.0004788	0.00133	0.62		down	MPHOSPH6
TC0X00569	0.0000666	0.00025	0.62		down	MST4
TC0700126	0.0016069	0.00386	0.62		down	NFE2L3
TC1300036	0.0013782	0.00337	0.62		down	O43649_HUMAN
TC0100628	0.0000067	0.000042	0.62		down	PDE4B
TC1200215	0.0000332	0.000147	0.62		down	PPFIBP1
TC1400281	0.0000127	0.0000675	0.62		down	PRKCH
TC1601030	0.0023647	0.00542	0.62		down	Q6XYE6_HUMAN
TC0400143	0.0000827	0.000298	0.62		down	RBPJ
TC1001004	0.0000471	0.000191	0.62		down	RHOBTB1
TC0701378	0.0001753	0.000562	0.62		down	SEMA3C
TC0400636	0.000042	0.000176	0.62		down	TMEM144
TC1800384	0.0006919	0.00184	0.62		down	ZNF521
TC0300393	0.0000043	0.0000308	0.61		down	CADM2
TC0201149	0.0002818	0.000842	0.61		down	CCL20
TC1101330	0.0000225	0.000107	0.61		down	CHST1
TC0200220	0.0000948	0.000332	0.61		down	CRIPT
TC0800979	0.0000019	0.0000175	0.61		down	CYP7B1
TC0800382	0.0003349	0.000976	0.61		down	LY96
TC0401339	0.0015889	0.00382	0.61		down	MAP9
TC2000438	0.0000082	0.0000486	0.61		down	NTSR1
TC0100589	0.0002422	0.000739	0.61		down	PRKAA2
TC0900248	0.0000945	0.000332	0.61		down	Q5VV11_HUMAN
TC0301580	0.000018	0.0000888	0.61		down	RAB6B
TC0300386	0.0000457	0.000187	0.61		down	ROBO2
TC0900295	0.0000059	0.0000382	0.61		down	RORB
TC0Y00103	0.0001655	0.000537	0.61		down	SPRY3
TC0Y00160	0.001922	0.00451	0.61		down	TTTY14
TC0101954	0.0000036	0.0000273	0.6		down	CAMK2N1
TC1400637	0.0000844	0.000302	0.6		down	CRIP2
TC0700821	0.0085439	0.0173	0.6		down	CTAGE15P
TC0401035	0.0000173	0.0000858	0.6		down	CXCL5
TC0301396	0.0000505	0.000202	0.6		down	DCBLD2
TC1300230	0.0000109	0.0000593	0.6		down	GPC6
TC0200301	0.0000145	0.0000744	0.6		down	MEIS1
TC1800380	0.0002522	0.000766	0.6		down	OSBPL1A
TC0500268	0.0000425	0.000177	0.6		down	RGNEF
TC1201128	0.0000284	0.00013	0.6		down	SLC38A1
TC1900148	0.0000012	0.0000129	0.59		down	ANGPTL4
TC1001003	0.0000128	0.0000677	0.59		down	ANK3
TC0301281	0.0000811	0.000293	0.59		down	ARHGEF3
TC0X01289	0.0000416	0.000174	0.59		down	ARHGEF6
TC0900638	0.0000021	0.0000188	0.59		down	ASS1
TC1800104	0.000007	0.0000435	0.59		down	CABLES1

TC0301413	0.0000094	0.0000535	0.59	down	CBLB
TC0301448	0.0000429	0.000178	0.59	down	CCDC80
TC0100221	0.0006954	0.00185	0.59	down	CDA
TC0X01201	0.0000771	0.000281	0.59	down	CUL4B
TC0400657	0.0000837	0.0003	0.59	down	FAM218A
TC0800176	0.0000102	0.0000565	0.59	down	FZD3
TC1000160	0.000389	0.00111	0.59	down	GJD4
TC0300508	0.0002896	0.000862	0.59	down	GRAMD1C
TC0601054	0.0001121	0.000385	0.59	down	HIST2H4A
TC0900091	0.0000013	0.0000136	0.59	down	IFNA1
TC0200624	0.0002114	0.000658	0.59	down	INSIG2
TC1101271	0.0000741	0.000272	0.59	down	LGR4
TC1101681	0.0000031	0.0000246	0.59	down	LRRC32
TC2100194	0.0001647	0.000535	0.59	down	PCBP3
TC0100924	0.0000398	0.000168	0.59	down	SEC22B
TC1200305	0.0000296	0.000135	0.59	down	SPATS2
TC1700229	0.0000366	0.000158	0.59	down	SPECC1
TC0X00422	0.0003008	0.000891	0.59	down	TMEM35
TC0200906	0.0000029	0.0000237	0.59	down	UBE2E3
TC0300626	0.0000006	0.00000769	0.58	down	ACPP
TC1001343	0.000003	0.0000242	0.58	down	ADAM12
TC0400471	0.0000302	0.000137	0.58	down	ANK2
TC0601300	0.0000679	0.000253	0.58	down	ENPP5
TC0801022	0.0000015	0.0000148	0.58	down	EYA1
TC0X01348	0.0000068	0.0000425	0.58	down	GABRE
TC0701547	0.0000038	0.0000283	0.58	down	LAMB1
TC0300019	0.0000048	0.0000334	0.58	down	LMCD1
TC1500465	0.000025	0.000117	0.58	down	NEO1
TC0901235	0.0000085	0.0000497	0.58	down	PTPN3
TC1400850	0.0000199	0.0000966	0.58	down	PYGL
TC1600314	0.0000095	0.0000539	0.58	down	QPRT
TC2000116	0.0000083	0.000049	0.58	down	RIN2
TC1700730	0.0000411	0.000173	0.58	down	SOX9
TC0800809	0.0000015	0.0000148	0.58	down	STC1
TC0400146	0.0001928	0.000611	0.58	down	STIM2
TC0100630	0.0033896	0.00747	0.58	down	TCTEX1D1
TC1201141	0.0001241	0.000422	0.58	down	VDR
TC0100667	0.0000026	0.0000217	0.57	down	AK5
TC1600572	0.0000008	0.00000948	0.57	down	CALB2
TC1800395	0.000039	0.000166	0.57	down	CDH2
TC1100265	0.0000522	0.000207	0.57	down	CK069_HUMAN
TC1800407	0.0000121	0.0000647	0.57	down	FAM59A
TC0600688	0.0000251	0.000118	0.57	down	GJA1
TC0100095	0.0000079	0.0000472	0.57	down	H6PD

TC0701089	0.0000853	0.000304	0.57	down	HOXA10
TC0301845	0.0000149	0.0000759	0.57	down	LEPREL1
TC2100224	0.0000089	0.0000513	0.57	down	NRIP1
TC0701370	0.000127	0.000431	0.57	down	PION
TC2000815	0.0000014	0.0000143	0.57	down	PREX1
TC0200433	0.0009075	0.00233	0.57	down	Q6ZW50_HUMAN
TC1800131	0.0000022	0.0000192	0.57	down	RNF125
TC0501249	0.0000191	0.000093	0.57	down	ZNF608
TC1500662	0.0000098	0.0000552	0.56	down	ARRDC4
TC0X00683	0.0000003	0.00000475	0.56	down	BGN
TC0600371	0.0000051	0.0000346	0.56	down	DAAM2
TC2200647	0.0017144	0.00409	0.56	down	ELFN2
TC0101825	0.0000086	0.00005	0.56	down	ERRFI1
TC0200787	0.0000296	0.000135	0.56	down	FMNL2
TC1700571	0.0000131	0.0000688	0.56	down	IGF2BP1
TC1201058	0.0000697	0.000258	0.56	down	ITPR2
TC1400994	0.0000006	0.00000769	0.56	down	LTBP2
TC0X00099	0.0014053	0.00343	0.56	down	PDK3
TC0300785	0.000089	0.000316	0.56	down	SKIL
TC0800365	0.0000259	0.000121	0.56	down	SULF1
TC0800801	0.0000011	0.0000121	0.56	down	TNFRSF10A
TC0300695	0.0000067	0.000042	0.55	down	AGTR1
TC1700713	0.0000024	0.0000204	0.55	down	ARSG
TC2100318	0.0000108	0.000059	0.55	down	ATP5O
TC0401047	0.0000346	0.000152	0.55	down	CDKL2
TC0900824	0.0000258	0.00012	0.55	down	hsa-mir-31
TC0501159	0.0000024	0.0000204	0.55	down	PCSK1
TC1800221	0.0000044	0.0000313	0.55	down	PMAIP1
TC0103421	0.0000012	0.0000129	0.55	down	SMYD3
TC1200206	0.0000168	0.0000839	0.55	down	SSPN
TC0200835	0.0000048	0.0000334	0.54	down	CERS6
TC0801134	0.0003407	0.00099	0.54	down	GDF6
TC0X01253	0.0000402	0.00017	0.54	down	GPC4
TC0601167	0.0000925	0.000326	0.54	down	HLA-DMB
TC0701090	0.000002	0.0000183	0.54	down	HOXA11
TC0700096	0.000003	0.0000242	0.54	down	ITGB8
TC1700723	0.0003876	0.00111	0.54	down	KCNJ2
TC0400555	0.0000275	0.000127	0.54	down	MGST2
TC0202230	0.0000009	0.0000102	0.54	down	MPP4
TC0202245	0.0000166	0.000083	0.54	down	RAPH1
TC1200216	0.0002778	0.000832	0.54	down	REP15
TC0700692	0.0000079	0.0000472	0.54	down	SMO
TC0300736	0.0000013	0.0000136	0.54	down	TIPARP
TC1700357	0.0000019	0.0000175	0.54	down	TMEM98

TC1701645	0.0000593	0.00023	0.53		down	ABCA8
TC0400409	0.0000018	0.0000169	0.53		down	BMPR1B
TC1600172	0.0000472	0.000191	0.53		down	CIITA
TC0801219	0.000009	0.0000516	0.53		down	HAS2
TC0601032	0.0000317	0.000142	0.53		down	HIST2H4A
TC0701082	0.0000182	0.0000894	0.53		down	HOXA2
TC1700689	0.0003121	0.000923	0.53		down	MILR1
TC0301502	0.0000022	0.0000192	0.53		down	MYLK
TC0401225	0.0000014	0.0000143	0.53		down	NDNF
TC0400144	0.0001201	0.000409	0.53		down	TBC1D19
TC2100118	0.0000718	0.000265	0.53		down	TTC3
TC0200412	0.0000005	0.00000688	0.53		down	VAMP8
TC0800392	0.0000298	0.000135	0.53		down	ZFHX4
TC0301711	0.0000017	0.0000162	0.52		down	B3GALNT1
TC0600300	0.0000024	0.0000204	0.52		down	HLA-DPB1
TC0400958	0.0000049	0.0000338	0.52		down	KDR
TC1200359	0.0000007	0.00000852	0.52		down	KRT18
TC0800233	0.000006	0.0000387	0.52		down	PLEKHA2
TC1101412	0.0000005	0.00000688	0.52		down	SLC43A3
TC0301277	0.0000076	0.0000458	0.52		down	WNT5A
TC0600013	0.0000064	0.000041	0.51		down	FOXC1
TC0700914	0.0000048	0.0000334	0.51		down	INSIG1
TC0600087	0.0001412	0.000472	0.51		down	RNF182
TC0601429	0.0000006	0.00000769	0.51		down	TBX18
TC0601768	0.0000002	0.00000349	0.51		down	THBS2
TC2000864	0.0000011	0.0000121	0.5		down	APCDD1L
TC0201530	0.0000018	0.0000169	0.5		down	EFEMP1
TC1100772	0.0000015	0.0000148	0.5		down	ENDOD1
TC2000566	0.0000007	0.00000852	0.5		down	FLRT3
TC0200803	0.0000397	0.000168	0.5		down	GALNT5
TC1800109	0.0000003	0.00000475	0.5		down	LAMA3
TC1201116	0.0000058	0.0000378	0.5		down	PRICKLE1
TC2100421	0.0000806	0.000292	0.5		down	Q9NSH7_HUMAN
TC1100393	0.0000001	0.00000202	0.5		down	SERPING1
TC0501500	0.0000015	0.0000148	0.5		down	STC2
TC0800800	0.0000001	0.00000202	0.5		down	TNFRSF10D
TC1401101	0.0000013	0.0000136	0.49		down	CLMN
TC0701083	0.0000863	0.000307	0.49		down	HOXA3
TC1200384	0.0000071	0.0000439	0.49		down	HOXC6
TC0400161	0.0000098	0.0000552	0.49		down	PGM2
TC2200343	0.0000013	0.0000136	0.49		down	PNPLA3
TC0102529	0.0000462	0.000189	0.49		down	SLC16A4
TC1700472	0.0000529	0.000209	0.49		down	WNK4
TC1201437	0.0000974	0.000339	0.48		down	ATP2B1

TC0700065	0.0000306	0.000139	0.48		down	MIOS
TC0201948	0.0000004	0.00000586	0.48		down	NCKAP5
TC0400539	0.0000149	0.0000759	0.48		down	PCDH10
TC0301635	0.0000074	0.000045	0.48		down	PLOD2
TC0901056	0.000001	0.0000112	0.48		down	PRUNE2
TC2200645	0.0000004	0.00000586	0.48		down	RAC2
TC1800163	0.0000001	0.00000202	0.48		down	SLC14A1
TC0301058	0.0000006	0.00000769	0.48		down	ZNF385D
TC0601601	0.0000687	0.000255	0.47		down	ARHGEF35
TC0500378	0.0000063	0.0000405	0.47		down	C5orf30
TC2000385	0.0000022	0.0000192	0.47		down	DOK5
TC0701081	0.0000083	0.000049	0.47		down	HOXA1
TC0701084	0.0000394	0.000167	0.47		down	HOXA4
TC0501311	0.0000005	0.00000688	0.47		down	KLHL3
TC1300044	0.0000026	0.0000217	0.47		down	WASF3
TC0102208	0.0000008	0.00000948	0.47		down	ZSWIM5
TC1800401	0.0000901	0.000319	0.46		down	B4GALT6
TC0301909	0.0000073	0.0000446	0.46		down	DLG1
TC1800098	0.0000048	0.0000334	0.46		down	GATA6
TC1200379	0.0000083	0.000049	0.46		down	HOXC11
TC0202289	0.000008	0.0000475	0.46		down	IKZF2
TC1000683	0.0000104	0.0000573	0.46		down	PTPRE
TC1100155	0.0000003	0.00000475	0.46		down	Q96C21_HUMAN
TC0X00475	0.0000005	0.00000688	0.46		down	RNF128
TC0500497	0.0000002	0.00000349	0.46		down	TGFBI
TC0400188	0.0000009	0.0000102	0.46		down	UCHL1
TC0900013	0.0000005	0.00000688	0.46		down	VLDLR
TC0800663	0.0000022	0.0000192	0.45		down	ANGPT2
TC1500650	0.0000051	0.0000346	0.45		down	MCTP2
TC1501002	0.000003	0.0000242	0.45		down	MYO1E
TC1101731	0.0000001	0.0000001	0.45		down	NOX4
TC0501298	0.0000196	0.0000953	0.45		down	PITX1
TC0X01066	0.0000429	0.000178	0.45		down	RPS6KA6
TC0200015	0.0000185	0.0000905	0.45		down	SOX11
TC0301750	0.0000008	0.00000948	0.45		down	TNIK
TC0701022	0.0000105	0.0000577	0.44		down	AGMO
TC0900163	0.0000015	0.0000148	0.44		down	CA9
TC1200654	0.0000019	0.0000175	0.44		down	CHST11
TC0202051	0.0000148	0.0000757	0.44		down	FAP
TC2000697	0.0000005	0.00000688	0.44		down	GDF5
TC1901418	0.0000004	0.00000586	0.44		down	HSPB6
TC0301701	0.0003826	0.0011	0.44		down	LXN
TC1701766	0.0000001	0.00000202	0.44		down	NPTX1
TC0600929	0.0001373	0.000462	0.44		down	NRN1

TC1100784	0.0000376	0.000162	0.44	down	Q96M56_HUMAN
TC1100970	0.0000261	0.000122	0.44	down	SLC37A2
TC0400363	0.0000001	0.00000202	0.43	down	AGPAT9
TC0701446	0.0000016	0.0000155	0.43	down	BAIAP2L1
TC1901622	0.0000063	0.0000405	0.43	down	CCDC8
TC0800047	0.0000262	0.000122	0.43	down	ERVK2
TC1200240	0.0000008	0.00000948	0.43	down	FGD4
TC0601170	0.0000039	0.0000288	0.43	down	HLA-DPA1
TC0500149	0.000007	0.0000435	0.43	down	LOC100129186
TC0700629	0.0000028	0.000023	0.43	down	MET
TC0401462	0.0000046	0.0000326	0.43	down	PDLIM3
TC0500918	0.0000001	0.0000001	0.43	down	RANBP3L
TC1300027	0.0000001	0.0000001	0.43	down	TNFRSF19
TC1500676	0.0000049	0.0000338	0.42	down	ALDH1A3
TC0800489	0.0000001	0.00000202	0.42	down	CTHRC1
TC0800903	0.0000002	0.00000349	0.42	down	PLAT
TC0X00424	0.0000018	0.0000169	0.41	down	DRP2
TC1800127	0.0000465	0.000189	0.41	down	DSG2
TC0400582	0.0000012	0.0000129	0.41	down	EDNRA
TC1900320	0.0000009	0.0000102	0.41	down	GDF15
TC0400935	0.0000009	0.0000102	0.41	down	OCIAD2
TC0601746	0.0000001	0.00000202	0.41	down	PDE10A
TC1300551	0.0000007	0.00000852	0.41	down	TBC1D4
TC0501451	0.0000002	0.00000349	0.4	down	ADAM19
TC0400663	0.0000012	0.0000129	0.4	down	CPE
TC0301735	0.0000007	0.00000852	0.4	down	MECOM
TC1401136	0.0000007	0.00000852	0.4	down	MOK
TC0900020	0.0000013	0.0000136	0.4	down	SLC1A1
TC0300436	0.0000031	0.0000246	0.4	down	ST3GAL6
TC0900826	0.0000001	0.0000001	0.39	down	CDKN2B
TC1701716	0.0000002	0.00000349	0.39	down	CYGB
TC0601168	0.0000001	0.00000202	0.39	down	HLA-DMA
TC1701478	0.0000014	0.0000143	0.39	down	HOXB2
TC0901057	0.0000057	0.0000373	0.39	down	PRUNE2
TC0300690	0.001225	0.00304	0.39	down	Q9P1D0_HUMAN
TC0500125	0.0000004	0.00000586	0.39	down	SLC1A3
TC1100785	0.0014723	0.00358	0.39	down	TMEM133
TC0101871	0.0000001	0.00000202	0.38	down	DHRS3
TC0400876	0.0001421	0.000475	0.38	down	GAFA3
TC0500147	0.0000035	0.0000268	0.38	down	GHR
TC1500656	0.0000001	0.00000202	0.38	down	NR2F2
TC1601328	0.0000001	0.00000202	0.38	down	SLC7A5
TC1201381	0.0000018	0.0000169	0.37	down	CPM
TC0500355	0.0000037	0.0000278	0.37	down	ERAP2

TC0500187	0.0000015	0.0000148	0.37	down	IL31RA
TC0300011	0.0000028	0.000023	0.37	down	ITPR1
TC0701645	0.0000013	0.0000136	0.37	down	PLXNA4
TC2100382	0.0000041	0.0000298	0.37	down	SIK1
TC0700585	0.0000007	0.00000852	0.36	down	LRRC17
TC0800393	0.0000099	0.0000555	0.36	down	PKIA
TC0100875	0.0000014	0.0000143	0.36	down	PTGFRN
TC1000292	0.0000002	0.00000349	0.36	down	TET1
TC0401281	0.0000024	0.0000204	0.35	down	CLGN
TC0100738	0.0000002	0.00000349	0.35	down	FNBP1L
TC0700622	0.0000001	0.00000202	0.35	down	FOXP2
TC0601159	0.000005	0.0000341	0.35	down	HLA-DQB1
TC0500930	0.0000095	0.0000539	0.35	down	LIFR
TC0101234	0.0000001	0.0000001	0.35	down	RGS4
TC0401332	0.0000222	0.000106	0.35	down	SFRP2
TC0401327	0.0000001	0.0000001	0.34	down	TMEM154
TC2100252	0.0000001	0.0000001	0.33	down	ADAMTS1
TC0600722	0.0000007	0.00000852	0.33	down	ENPP1
TC2000221	0.0000001	0.0000001	0.33	down	GDF5OS
TC0201216	0.0000001	0.0000001	0.33	down	MLPH
TC0101521	0.0000001	0.00000202	0.33	down	TGFB2
TC0Y00071	0.0000006	0.00000769	0.32	down	EIF1AY
TC1100241	0.0000014	0.0000143	0.32	down	FIBIN
TC1600623	0.0000598	0.000231	0.32	down	HSD17B2
TC1601110	0.0000004	0.00000586	0.32	down	IRX3
TC1701357	0.0000006	0.00000769	0.32	down	KRT19
TC0601537	0.0000002	0.00000349	0.32	down	LAMA4
TC1500733	0.0000002	0.00000349	0.32	down	NDN
TC0900198	0.0000002	0.00000349	0.32	down	Q4VXF0_HUMAN
TC2100293	0.0000001	0.0000001	0.32	down	TIAM1
TC1700158	0.0000001	0.0000001	0.32	down	TRPV2
TC0700087	0.0000001	0.0000001	0.32	down	TSPAN13
TC0Y00165	0.0000007	0.00000852	0.31	down	KDM5D
TC0400166	0.0000021	0.0000188	0.31	down	KLHL5
TC1800208	0.0000003	0.00000475	0.31	down	NEDD4L
TC1201020	0.0000003	0.00000475	0.3	down	ARHGDIB
TC0200207	0.0000005	0.00000688	0.3	down	PLEKHH2
TC1501100	0.0000001	0.0000001	0.3	down	SEMA7A
TC0501310	0.0000001	0.0000001	0.3	down	SPOCK1
TC0800389	0.0000001	0.0000001	0.29	down	CRISPLD1
TC0200883	0.0000003	0.00000475	0.29	down	HOXD8
TC0202040	0.0000019	0.0000175	0.29	down	PLA2R1
TC1000637	0.0000001	0.0000001	0.29	down	PPAPDC1A
TC0Y00070	0.0000001	0.0000001	0.29	down	TXLNG2P

TC0701088	0.0000001	0.00000202	0.28		down	HOXA9
TC0401291	0.0000001	0.00000202	0.28		down	INPP4B
TC1700547	0.0000001	0.0000001	0.28		down	ITGB3
TC0400151	0.0000001	0.0000001	0.28		down	PCDH7
TC0102558	0.0000001	0.0000001	0.28		down	PTPN22
TC0501563	0.0000006	0.00000769	0.27		down	GFPT2
TC0500902	0.0000001	0.0000001	0.26		down	ADAMTS12
TC0500969	0.0000002	0.00000349	0.26		down	EMB
TC1200382	0.0000002	0.00000349	0.26		down	HOXC9
TC0701079	0.0000011	0.0000121	0.25		down	SKAP2
TC2100253	0.0000001	0.00000202	0.24		down	ADAMTS5
TC0201781	0.0000001	0.0000001	0.24		down	AFF3
TC1500431	0.0000001	0.0000001	0.24		down	CORO2B
TC1200882	0.0000044	0.0000313	0.24		down	LOC100128816
TC0900822	0.0000001	0.00000202	0.24		down	MIR31HG
TC0900924	0.0000001	0.00000202	0.23		down	CNTNAP3
TC0102380	0.0000002	0.00000349	0.23		down	ELTD1
TC0202064	0.0000001	0.0000001	0.23		down	GALNT3
TC0601156	0.0000005	0.00000688	0.23		down	HLA-DRB5
TC1300447	0.0000007	0.00000852	0.23		down	KCTD4
TC1000098	0.0000001	0.0000001	0.23		down	PLXDC2
TC1800238	0.0000006	0.00000769	0.23		down	SERPINB7
TC1400328	0.0000001	0.0000001	0.23		down	SMOC1
TC0401016	0.0000001	0.0000001	0.23		down	SULT1B1
TC0202185	0.0000001	0.0000001	0.23		down	TMEFF2
TC0401359	0.0000001	0.0000001	0.22		down	FAM198B
TC1200383	0.0000002	0.00000349	0.22		down	HOXC8
TC0Y00056	0.0000001	0.0000001	0.22		down	NLGN4Y
TC1201423	0.0000001	0.0000001	0.22		down	SLC6A15
TC0301144	0.0000001	0.0000001	0.21		down	CDCP1
TC0X00397	0.0000001	0.0000001	0.21		down	KLHL4
TC0202126	0.0000072	0.0000442	0.21		down	LOC375295
TC1601077	0.0000001	0.00000202	0.21		down	NETO2
TC0Y00012	0.0000001	0.0000001	0.2		down	ZFY
TC1800188	0.0000001	0.00000202	0.19		down	DCC
TC0300182	0.0000001	0.00000202	0.19		down	ENTPD3
TC1300644	0.0000002	0.00000349	0.19		down	FAM155A
TC0500623	0.0000001	0.0000001	0.19		down	GRIA1
TC0801100	0.0000003	0.00000475	0.19		down	MMP16
TC0801101	0.0000001	0.0000001	0.19		down	MMP16
TC2100092	0.0000001	0.0000001	0.19		down	MRPS6
TC1200525	0.0000001	0.0000001	0.19		down	TRHDE
TC0Y00143	0.0000001	0.0000001	0.19		down	UTY
TC0100770	0.0000001	0.0000001	0.19		down	VCAM1

TC0202049	0.0000001	0.0000001	0.18	down	DPP4
TC0800838	0.0000001	0.0000001	0.18	down	DUSP4
TC2100124	0.0000001	0.0000001	0.18	down	KCNJ15
TC0701023	0.0000001	0.00000202	0.18	down	MEOX2
TC0Y00011	0.0000001	0.0000001	0.18	down	RPS4Y1
TC0900218	0.0000001	0.0000001	0.17	down	CNTNAP3B
TC0701087	0.0000005	0.00000688	0.17	down	HOXA7
TC1501066	0.0000001	0.0000001	0.17	down	ITGA11
TC0900510	0.0000001	0.0000001	0.17	down	PAPPA
TC1800193	0.0000002	0.00000349	0.17	down	RAB27B
TC0501153	0.0000001	0.0000001	0.16	down	MCTP1
TC0102459	0.0000001	0.00000202	0.15	down	ARHGAP29
TC0500956	0.0000001	0.0000001	0.15	down	CCL28
TC0501408	0.0000001	0.00000202	0.15	down	CD74
TC0400570	0.0000001	0.0000001	0.15	down	HHIP
TC0900518	0.0000001	0.0000001	0.15	down	TLR4
TC1201056	0.0000004	0.00000586	0.14	down	BHLHE41
TC0102199	0.0000003	0.00000475	0.14	down	NR_002755.2
TC0Y00051	0.0000001	0.0000001	0.14	down	USP9Y
TC1800488	0.0000001	0.0000001	0.13	down	CCDC68
TC0901284	0.0000001	0.0000001	0.12	down	Q5QFB8_HUMAN
TC0901283	0.0000001	0.0000001	0.12	down	Q5QFB9_HUMAN
TC1701642	0.0000001	0.0000001	0.12	down	SLC16A6
TC0700704	0.0000001	0.0000001	0.11	down	CPA4
TC0700709	0.0000001	0.0000001	0.11	down	MEST
TC0600327	0.0000001	0.0000001	0.11	down	SCUBE3
TC1200380	0.0000013	0.0000136	0.1	down	HOXC10
TC0900244	0.0000001	0.0000001	0.095	down	LOC643792
TC1300640	0.0000001	0.0000001	0.094	down	EFNB2
TC0400381	0.0000001	0.0000001	0.092	down	SPP1
TC0600293	0.0000001	0.0000001	0.088	down	HLA-DRA
TC0400885	0.0000001	0.0000001	0.084	down	TMEM156
TC0X00470	0.0000001	0.0000001	0.082	down	NRK
TC0Y00052	0.0000001	0.0000001	0.081	down	DDX3Y
TC0701086	0.0000021	0.0000188	0.08	down	HOXA6
TC1200030	0.0000001	0.0000001	0.07	down	CCND2
TC1400549	0.0000001	0.0000001	0.057	down	NR_003213.1
TC0400595	0.0000001	0.0000001	0.055	down	MAB21L2
TC0701085	0.0000001	0.0000001	0.053	down	HOXA5
TC0800396	0.0000001	0.0000001	0.052	down	STMN2
TC0102595	0.0000001	0.0000001	0.036	down	TBX15

Supplementary table 3.sigal-net

Gene symbol	betweenness centrality	degree	indegree	outdegree	style
PLCB4	0.002534014	7	7	3	up
FLNB	0.001559393	6	6	6	down
CAMK2G	0.001403454	2	1	2	up
GRIA1	0.001091575	3	2	1	down
RAC2	0.000935636	8	4	4	down
MAPK13	0.000935636	8	4	4	down
ITGA4	0.000929139	12	10	4	down
LYN	0.000623757	3	2	1	up
CCND2	0.000389848	5	4	1	down
CDC25B	0.000389848	2	1	1	up
FZD3	0.000363858	5	4	1	down
FZD4	0.000363858	5	4	1	down
FZD7	0.000363858	5	4	1	up
CDK1	0.000350864	5	4	1	up
CASP3	0.000233909	5	3	2	down
RBL1	0.000233909	2	2	1	up
PREX1	0.000194924	2	1	1	down
PLXNA2	0.000194924	2	1	2	up
ITPR1	0.000136447	3	3	1	down
ITPR2	0.000136447	3	3	1	down
TGFB2	0.000116955	3	2	1	down
HLA-DMA	0.000116955	8	7	8	down
HLA-DMB	0.000116955	8	7	8	down
RASGRF2	0.000116955	2	2	2	up
ITGA11	0.000110457	10	9	2	down
ITGB3	0.000110457	10	9	2	down
ITGB8	0.000110457	10	9	2	down
ITGA2	0.000110457	10	9	2	up
ITGA8	0.000110457	10	9	2	up
PTTG1	7.79697E-05	2	1	1	up
ARRB1	7.79697E-05	2	2	2	up
CACNA1C	7.79697E-05	2	2	1	up
ESPL1	7.79697E-05	2	1	1	up
NOTCH3	7.79697E-05	3	2	1	up
LTBP1	7.79697E-05	2	1	1	up
CDKN2B	5.84773E-05	2	1	1	down
STAT4	5.84773E-05	2	1	1	down
MET	4.15838E-05	6	5	1	down
IGF1R	4.15838E-05	6	5	1	up
PDGFRA	4.15838E-05	6	5	1	up
CDK2	3.89848E-05	2	1	1	up
PDGFRB	2.59899E-05	4	3	1	up
KDR	2.20914E-05	4	3	1	down
FLT1	2.20914E-05	4	3	1	up
ADRB2	1.94924E-05	2	2	1	up
F2R	1.94924E-05	2	2	1	up
HLA-DRA	0	7	7	7	down
SPP1	0	6	0	6	down
CD74	0	3	3	0	down
DUSP4	0	1	0	1	down
DCC	0	1	1	0	down
VCAM1	0	2	2	1	down
HLA-DRB5	0	7	7	7	down
NEDD4L	0	6	0	6	down
TIAM1	0	1	0	1	down

LAMA4	0	6	0	6	down
LIFR	0	1	1	0	down
SFRP2	0	5	0	5	down
HLA-DQB1	0	7	7	7	down
GHR	0	1	1	0	down
HLA-DPA1	0	7	7	7	down
GDF5	0	1	1	0	down
WASF3	0	1	1	0	down
LAMA3	0	6	0	6	down
THBS2	0	7	0	7	down
WNT5A	0	5	2	3	down
HLA-DPB1	0	7	7	7	down
MYLK	0	1	1	0	down
GDF6	0	1	1	0	down
GJA1	0	2	2	0	down
LAMB1	0	6	0	6	down
IFNA1	0	2	0	2	down
CBLB	0	10	0	10	down
ARHGEF6	0	1	0	1	down
RBPJ	0	1	1	0	down
COL11A1	0	6	0	6	down
PDGFA	0	6	0	6	down
LAMC2	0	6	0	6	down
TGFBR1	0	3	3	0	down
RHOD	0	1	1	1	down
IFNAR1	0	2	2	0	down
HLA-DOA	0	7	7	7	down
PIAS2	0	2	0	2	down
IL1RAP	0	2	0	2	down
E2F5	0	1	1	1	down
MRVI1	0	2	0	2	down
STK4	0	1	1	0	up
IRAK4	0	2	2	0	up
CDC25C	0	1	0	1	up
NUMB	0	1	0	1	up
PCNA	0	1	0	1	up
CDC25A	0	2	0	2	up
CCNA2	0	1	0	1	up
LAMA2	0	6	0	6	up
CDC6	0	2	2	0	up
CRLF2	0	1	1	0	up
LGMN	0	1	0	1	up
PRKX	0	6	0	6	up
SMC1A	0	1	1	0	up
DUSP5	0	1	0	1	up
NOG	0	3	0	3	up
CDKN2C	0	1	0	1	up
PSME1	0	1	1	0	up
IL1R1	0	3	0	3	up
JAG1	0	1	0	1	up
WNT2B	0	5	2	3	up
DUSP10	0	1	0	1	up
CASP10	0	1	0	1	up
MAPKAPK3	0	1	1	0	up
CACNA1A	0	1	1	1	up
DUSP1	0	1	0	1	up
FGF5	0	4	0	4	up
ABLIM3	0	1	1	0	up

PTK2	0	13	13	0	up
BMP4	0	1	1	0	up
CDC20	0	1	0	1	up
IQGAP3	0	1	1	0	up
WEE1	0	1	0	1	up
BIRC3	0	1	0	1	up
DCN	0	1	0	1	up
CYFIP2	0	1	1	0	up
SFRP1	0	5	0	5	up
GNG11	0	2	0	2	up
IRAK3	0	2	2	0	up
GRIA3	0	1	0	1	up
IL8	0	1	1	0	up
FGF7	0	4	0	4	up
GRK5	0	2	0	2	up
CXCL1	0	1	1	0	up
